# Protein domains and architectural innovation in plant-associated Proteobacteria

**DOI:** 10.1186/1471-2164-6-17

**Published:** 2005-02-16

**Authors:** David J Studholme, J Allan Downie, Gail M Preston

**Affiliations:** 1 The Sainsbury Laboratory, Norwich, NR4 7UH, UK; 2 Department of Molecular Microbiology, John Innes Centre, Norwich, NR4 7UH, UK; 3 Department of Plant Sciences, University of Oxford, Oxford, OX1 3RB, UK

## Abstract

**Background:**

Evolution of new complex biological behaviour tends to arise by novel combinations of existing building blocks. The functional and evolutionary building blocks of the proteome are protein domains, the function of a protein being dependent on its constituent domains. We clustered completely-sequenced proteomes of prokaryotes on the basis of their protein domain content, as defined by Pfam (release 16.0). This revealed that, although there was a correlation between phylogeny and domain content, other factors also have an influence. This observation motivated an investigation of the relationship between an organism's lifestyle and the complement of domains and domain architectures found within its proteome.

**Results:**

We took a census of all protein domains and domain combinations (architectures) encoded in the completely-sequenced proteobacterial genomes. Nine protein domain families were identified that are found in phylogenetically disparate plant-associated bacteria but are absent from non-plant-associated bacteria. Most of these are known to play a role in the plant-associated lifestyle, but they also included domain of unknown function DUF1427, which is found in plant symbionts and pathogens of the alpha-, beta- and gamma-Proteobacteria, but not known in any other organism. Further, several domains were identified as being restricted to phytobacteria and Eukaryotes. One example is the RolB/RolC glucosidase family, which is found only in *Agrobacterium *species and in plants. We identified the 0.5% of Pfam protein domain families that were most significantly over-represented in the plant-associated Proteobacteria with respect to the background frequencies in the whole set of available proteobacterial proteomes. These included guanylate cyclase, domains implicated in aromatic catabolism, cellulase and several domains of unknown function.

We identified 459 unique domain architectures found in phylogenetically diverse plant pathogens and symbionts that were absent from non-pathogenic and non-symbiotic relatives. The vast majority of these were restricted to a single species or several closely related species and so their distributions could be better explained by phylogeny than by lifestyle. However, several architectures were found in two or more very distantly related phytobacteria but absent from non-plant-associated bacteria. Many of the proteins with these unique architectures are predicted to be secreted.

In *Pseudomonas syringae *pathovar *tomato*, those genes encoding genes with novel domain architectures tended to have atypical GC contents and were adjacent to insertion sequence elements and phage-like sequences, suggesting acquisition by horizontal transfer.

**Conclusions:**

By identifying domains and architectures unique to plant pathogens and symbionts, we highlighted candidate proteins for involvement in plant-associated bacterial lifestyles. Given that characterisation of novel gene products *in vivo *and *in vitro *is time-consuming and expensive, this computational approach may be useful for reducing experimental search space. Furthermore we discuss the biological significance of novel proteins highlighted by this study in the context of plant-associated lifestyles.

## Background

The Proteobacteria comprise a phylum of Gram-negative bacteria that includes an extraordinary diversity of lifestyles, ecology and metabolism. At one end of a spectrum are free-living organisms such as *Pseudomonas aeruginosa*, which has a relatively large genome that encodes enormous regulatory and metabolic flexibility, allowing it to colonise diverse niches. At the other extreme are highly specialised intracellular symbionts (*Buchnera *species, *Rickettsia *species), whose small genomes have undergone reductive evolution and which lack many common metabolic and regulatory features. With the availability of complete genome sequences for many model plant-associated bacteria, we are particularly interested in how genome analyses can be used to gain insights into the mechanisms and evolution of associations between bacteria and plants.

There are complete annotated genome sequences available for several phylogenetically diverse proteobacterial plant pathogens and symbionts, along with many of their non-pathogenic and non-symbiotic relatives. For example, among the alpha-Proteobacteria, complete genome sequences are available for the phytopathogen *Agrobacterium tumefaciens *[1-3], the nitrogen-fixing symbionts *Bradyrhizobium japonicum *[4], *Mesorhizobium loti *[5] and *Sinorhizobium meliloti *[6,7], the non-pathogenic free-living *Caulobacter crescentus *[8], and the animal pathogenic *Rickettsia *species [9-11]. *Ralstonia solanacearum *[12] is the sole completely sequenced plant pathogen amongst the beta-Proteobacteria, a division that also includes animal pathogens in the genera *Neisseria *[13,14] and *Bordetella *[15] and the free-living chemolithoautotroph *Nitrosomonas europaea *[16] whose genomes have been sequenced. Among the available complete genome sequences for the gamma-Proteobacteria are those of the plant pathogens *Xylella fastidiosa *[17,18], *Xanthomonas campestris *[19], *Xanthomonas axonopodis *[19] and *Pseudomonas syringae *pathovar *tomato *[20] as well as *P. aeruginosa *[21], which is an occasional pathogen of plants as well as animals.

Each of these three divisions of the Proteobacteria contains a wide variety of different lifestyles, so it is logical to assume that bacteria-plant interactions have evolved independently in multiple separate Proteobacterial lineages. Ultimately the differences between these lifestyles are determined by the organisms' genes acting through their expressed proteins and RNAs. Given the abundance of complete genome sequence data now available, a high priority is to understand which features of an organism's proteome determine its lifestyle, and the evolutionary processes underlying environmental adaptation and evolution of novel traits. Two main sources have been proposed for the evolution and acquisition of novel traits by bacteria: (i) duplication, mutation and recombination of existing genes within a single lineage, and (ii) lateral gene transfer between lineages. A combination of both bioinformatic and experimental studies are needed to determine the relative importance of these two processes in the evolution of plant-associated lifestyles in bacteria.

Evolution of new complex biological behaviours tends to arise (but not exclusively) by novel combinations of existing building blocks. The functional and evolutionary building blocks or units of the proteome are protein domains. Protein domains can be classified into families; examples of widely used classification schemes are those of Pfam [23] and SMART [24]. We hypothesised that systematic identification of proteins having domain architectures that are exclusive to plant-associated bacteria would identify good candidates for proteins with specific involvement in plant-microbial interactions, or in a plant-associated lifestyle, and would also generate insight into the distribution and evolution of novel traits in plant-associated bacteria.

## Results and discussion

### Hierarchical clustering of completely-sequenced prokaryotic proteomes

**Figure 1:**
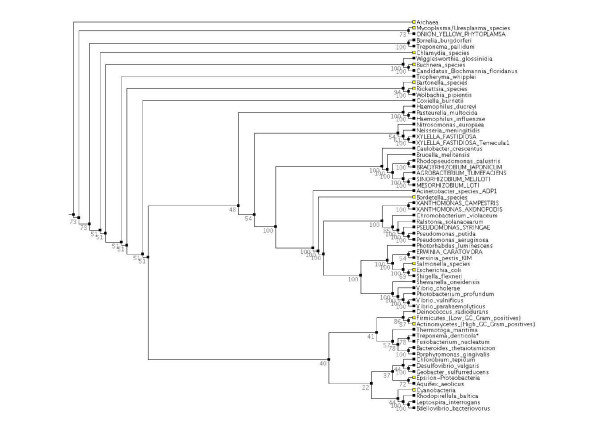
**Clustering of complete prokaryotic proteomes based on their protein domain content**. 100 jacknife trials were performed, each leaving out a random 10% of the data.

To gain an overview of the similarities and differences between their protein domain content, we classified representative prokaryotes into hierarchical clusters based on their complement of protein domain families described. For each proteome we generated a 7,677 binary state element vector where each element represented the presence or absence of one of the 7,677 Pfam protein domain families. Pairwise distances were calculated for each pair of proteomes based on the level of similarity between the pair of vectors, and tree was built by neighbour-joining (see Methods for more details). One hundred trees were built, each time leaving out 10 % of the vector elements, selected at random. The tree shown in Figure 1 represents the consensus of these 100 jacknife trials.

The tree in Figure 1 illustrates the similarities and differences between prokaryotes with respect to their repertoire of recognisable protein domain families. There is clearly a correlation between domain complement and phylogeny; for example, the Archaea form a distinct cluster that is clearly separated from the Bacteria. Furthermore, within the Bacteria, the Cyanobacteria, Gram-positive Bacteria, chlamydias and mycoplasmas each fall into distinct clusters. However, there are some striking discrepancies between the protein domain-based clustering and phylogenetic classification. For example, the oral pathogen *Treponema denticola *(marked with an asterisk in Figure 1) clusters with the dental bacterium *Fusobacterium nucleatum *rather than with its fellow spirochetes *T. pallidum *and *Borrelia burgdorferi*.

It is notable that the Proteobacteria do not form a single distinct cluster in the protein-domain based classification in Figure 1. The cluster that contains the gamma-proteobacterial *Pseudomonas *and *Xanthomonas *species also contains the beta-Proteobacteria *R. solanacearum *and *Chromobacterium violaceum*. This probably reflects that these organisms have relatively large genomes and therefore share in common some common protein domains that are not encoded in smaller more streamlined genomes. Conversely *X*. *fastidiosa*, which has a relatively small genome, falls into a cluster with *Neisseria meningitidis*.

Interestingly, the plant pathogen *E. caratovora *fell into a cluster with *Yersinia pestis*, *Salmonella *species and *E. coli*, which are animal pathogens and commensals. This indicates that despite differing lifestyles, these species have diverged relatively little with respect to loss and gain of protein domain families.

Overall, the results of clustering bacterial proteomes on the basis of their domain content suggested that in addition to phylogeny, an organism's domain repertoire may reflect other factors, possibly including genome size and lifestyle. These preliminary observations led us to investigate whether it is possible to identify any particular domains or domain architectures that may be characteristic of a plant-associated lifestyle.

### Protein domain families restricted to plant-associated bacteria

We queried the Pfam 16.0 database to determine the species distribution of each of the 7,677 domain families. Of these, 85 were found in at least one of the completely sequenced plant associated bacteria but absent from all other completely sequenced bacteria. Most of these domain families are restricted to a single species or group of very closely related organisms. For example, domain of unknown function DUF1484 (Pfam:PF07363) appears to be restricted to *Ralstonia solanacearum*, whilst DUF1520 (Pfam:PF07480) is restricted to *Bradyrhizobium japonicum *and *Sinorhizobium meliloti*. Although it is possible that these species-specific domain families are involved in pathogenesis or symbiosis it is equally likely that they have some unrelated function. However, several domains are potentially interesting from the point of view of plant-microbe interactions either because they are found in phylogenetically disparate species of phytobacteria or because they are also found in eukaryotes. Table 1 lists the domain families that are found in plant-associated members of more than one subdivision of the Proteobacteria, but are not found in any non-plant-associated bacteria. Several of these are already implicated in host-plant interactions. For example, proteins belonging to the NolX family (Pfam:PF05819) include HrpF from the gamma-proteobacterium *X. campestris *and NolX from the alpha-proteobacterium *Rhizobium fredii and Rhizobium *species NGR234. In these rhizobia, NolX (also referred to as NopX) has been shown to play a role in nodulation specificity and is exclusively expressed during the early stages of interactions with plants [25,26]. NolX is thought to facilitate protein secretion into the plant host via a type III secretion system [27], and a similar role has been postulated for *X. campestris *HrpF [28]. The importance of members of the NolX family in microbe-plant interactions is reinforced by our observation that they are also found in several other plant-associated alpha- and gamma-Proteobacteria as well as in the phytopathogenic beta-proteobacterium *R. solanacearum *(see Table 1), but are not found in any other completely sequenced genomes. Similarly, the Avirulence domain (Pfam:PF03377) is restricted to the phytopathogens *R. solanacearum *and *Xanthomonas *species [29].

**Table 1:** Pfam protein domain families found in phylogentically disparate plant-associated bacteria and not found in non-plant associated bacteria.

**Pfam domain family**	**Species distribution**
Avirulence PF03377 X. avirulence protein, Avr/PthA	*R. solanacearum; X. axonopodis (pv. citri); X. campestris (pv. citri); X. campestris (pv. vesicatoria); X. campestris; X. manihotis; X. oryzae (pv. oryzae); X. oryzae;*
DspF PF06704 DspF/AvrF protein	*Erwinia amylovora; E. carotovora subsp. atroseptica SCRI1043; Erwinia pyrifoliae; Erwinia stewartii; Pantoea agglomerans (pv. gypsophilae) (Erwinia herbicola); Pectobacterium atrosepticum; P. syringae (pv. tomato); P. syringae;*
DUF1427 PF07235 Domain of unknown function	*A. tumefaciens (strain C58 / ATCC 33970); B. japonicum; P. aeruginosa; R. solanacearum; Rhizobium leguminosarum (biovar trifolii); Rhizobium meliloti (Sinorhizobium meliloti); X. campestris (pv. campestris);*
DUF811 PF05665 Domain of unknown function	*P. aeruginosa; R. solanacearum;*
HrpE PF06188 HrpE protein	*Erwinia amylovora; E. carotovora subsp. atroseptica SCRI1043; Erwinia chrysanthemi; Erwinia pyrifoliae; Erwinia stewartii; Pectobacterium atrosepticum; Pectobacterium carotovorum (subsp. carotovorum) (E. carotovora (subsp. carotovora)); P. fluorescens; P. syringae (pv. glycinea); P. syringae (pv. phaseolicola); P. syringae (pv. savastanoi); P. syringae (pv. syringae); P. syringae (pv. tabaci); P. syringae (pv. tomato); P. syringae;*
HrpF PF06266 HrpF protein	*Erwinia amylovora; E. carotovora subsp. atroseptica SCRI1043; Erwinia chrysanthemi; Erwinia pyrifoliae; Erwinia stewartii; Pectobacterium atrosepticum;Pectobacterium carotovorum (subsp. carotovorum) (E. carotovora (subsp. carotovora)); P. syringae (pv. glycinea); P. syringae (pv. phaseolicola); P. syringae (pv. savastanoi); P. syringae (pv. syringae); P. syringae (pv. tabaci); P. syringae (pv. tomato);*
Ice_nucleation PF00818 Ice nucleation protein repeat	*Bordetella phage BPP-1; Erwinia herbicola; Pantoea ananas (Erwinia uredovora); P. fluorescens; P. syringae (pv. syringae); P. syringae; X. campestris (pv. campestris); X. campestris (pv. translucens); *
NolX PF05819 NolX protein	*R. solanacearum; Rhizobium fredii (Sinorhizobium fredii); Mesorhizobium loti; Rhizobium sp. (strain NGR234); X. axonopodis (pv. citri); X. axonopodis pv. glycines; X. campestris (pv. campestris); X. campestris (pv. vesicatoria); X. oryzae (pv. oryzae); *
VirK PF06903 VirK protein	*A. tumefaciens (strain C58 / ATCC 33970); A. tumefaciens; B. japonicum; P. syringae (pv. tomato); R. solanacearum; Rhizobium sp. (strain NGR234); X. axonopodis (pv.citri); X. campestris (pv. campestris); X. fastidiosa (strain Temecula1 / ATCC 700964); X. fastidiosa;*

**Figure 2:**
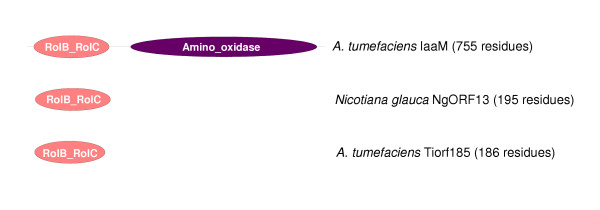
Examples of proteins containing a RolB/RolC domain.

A further protein family limited to plant-associated bacteria is characterised by the ice nucleation repeat (Pfam:PF00818)and is found in proteins that may have a role in frost damage to host plants. It remains to be seen whether the remaining two domain families (DUF811 and DUF1427) are involved in the plant-associated lifestyle. DUF1427 (Pfam:PF07235) is restricted to several plant-associated alpha-Proteobacteria, the beta-proteobacterium *R. solanacearum *and the gamma-Proteobacteria *P. aeruginosa *and *X. campestris *(Table 1). Although their functions are unknown, proteins containing DUF1427 are thus candidates for involvement in interactions with plants or may at least have a role in plant-associated lifestyles. Several of these proteins have predicted signal peptide sequences and / or predicted transmembrane regions, suggesting an extracytoplasmic location. This may be indicative of a role in extracellular interactions with plants or with other components of the environment. Table 2 lists the 13 protein domain families that appear to be restricted to plant-associated bacteria and to eukaryotes and/or Archaea. Interestingly, this highlights at least one example of a protein domain that has probably been recruited into plant-associated bacteria from a plant host. Proteins containing a RolB/RolC-like domain (Pfam:PF02027) are found to be restricted to plant-associated alpha-Proteobacteria and to plants of the genus *Nicotiana *(see Table 2 and Figure 2). The activity of these proteins in plants may lead to an increase in intracellular auxin activity caused by the release of active auxins from inactive beta-glucosides [30,31]. The presence of many *Agrobacterium-*like proteins in *Rhizobium (Agrobacterium) vitis *reflects another key feature of the biology of these plant-associated bacteria, the fact that many of the genes involved directly in *Agrobacterium *and *Rhizobium- *plant interactions are encoded on large plasmids that facilitate lateral gene transfer of complex and novel traits between bacteria. *Rhizobium (Agrobacterium) vitis *is not a symbiont, but rather causes a tumorigenic disease of grapevine through the action of a number of *A. tumefaciens-*like genes [32].

**Table 2:** Pfam protein domain families restricted to plant-associated bacteria and eukaryotes.

**Pfam domain family**	**Species distribution (not exhaustive)**
CBM_14 PF01607 Chitin binding Peritrophin-A domain	*Ralstonia solanacearum; *Metazoa; Fungi; Viruses
CD225 PF04505 Interferon- induced transmembrane protein	*Xanthomonas campestris (pv campestris); *Metazoa;
DUF726 PF05277 Protein of unknown function (DUF726)	*Pseudomonas syringae *(pv *tomato*)*; *Metazoa; Plants;
DUF763 PF05559 Protein of unknown function (DUF763)	*Mesorhizobium loti; Sinorhizobium meliloti; Xanthomonas axonopodis (pv. citri); Xanthomonas campestris (pv. campestris); *Archaea;
GDA1_CD39 PF01150 GDA1/CD39 (nucleoside phosphatase) family	*Pseudomonas syringae (pv. Tomato); *Plants; Fungi; Metazoa;
Het-C PF07217 Heterokaryon incompatibility protein Het-C	*Pseudomonas syringae (pv. tomato); *Fungi;
PAX PF00292 'Paired box' domain	*Rhizobium etli; Mesorhizobium loti; *Metazoa;
PPR PF01535 PPR repeat	*Ralstonia solanacearum; *Plants; Metazoa; Fungi;
Rhamnogal_lyase PF06045 Rhamnogalacturonate lyase family	*Erwinia carotovora subsp. atroseptica SCRI1043; Erwinia chrysanthemi; Plants;*
Ribosomal_60s PF00428 60s Acidic ribosomal protein	*Ralstonia solanacearum (Pseudomonas solanacearum); Plants; Metazoa; Archaea;*
RolB_RolC PF02027 RolB/RolC glucosidase family	*Agrobacterium rhizogenes; Agrobacterium tumefaciens (strain Ach5), and Agrobacterium tumefaciens (strain 15955); Agrobacterium tumefaciens (strain Ach5), and Agrobacterium tumefaciens; Agrobacterium tumefaciens (strain Ach5); Agrobacterium tumefaciens (strain C58 / ATCC 33970); Agrobacterium tumefaciens; Agrobacterium vitis (Rhizobium vitis); Plants;*
SBP56 PF05694 56 kDa selenium binding protein (SBP56)	*Bradyrhizobium japonicum; ; Plants; Metazoa; Archaea;*
ST7 PF04184 ST7 protein	*Rhizobium loti (Mesorhizobium loti); Metazoa;*

### Protein domain families that are over-represented in plant-associated bacteria

Bacterial physiology and behaviour is determined not only by the presence or absence of particular proteins but also by numbers of representatives of protein families. For example, gene duplication events may lead to a lineage-specific expansion that results in novel orthologues that can take on novel functions different from that of the parent gene. Therefore we investigated whether any protein domain families were over-represented in the plant-associated proteobacteria with respect to the background distribution of domains in all Proteobacteria for which complete sequences were available. For each of the 7,677 Pfam domain families, we counted the numbers of proteins in which that domain family occurs in the complete proteomes of *Erwinia carotovora*, *Pseudomonas syringae *pathovar *tomato*, *Ralstonia solanacearum*, *Sinorhizobium meliloti*, *Bradyrhizobium japonicum*, *Mesorhizobium loti*, *Agrobacterium tumefaciens *(Washington strain and Dupont strain), *Xanthomonas campestris *pathovar *campestris*, *Xanthomonas axonopodis *pathovar *citri*, *Xylella fastidiosa *and *Xylella fastidiosa *(strain Temecula1). We then calculated a P value for the probability of observing at least this number of occurrences given the background frequency in the Proteobacteria and assuming a binomial distribution. The smaller the P value, the less likely that the observed frequency occurred by chance. In other words, the smaller the P value, the more over-represented is the domain family. The most over-represented domains are listed in Table 3.

**Table 3:** Protein domain families over-represented in plant-associated proteobacteria.

Domain family		Expected number of proteins	Observed number of proteins	P
			
Pfam accesion	Pfam ID			
PF00211	Guanylate_cyc	33.39	70	2.17E-008
PF00296	Bac_luciferase	46.36	81	2.56E-006
PF04828	DUF636	36.58	65	1.40E-005
PF04679	DNA_ligase_A_C	17.65	38	1.76E-005
PF01068	DNA_ligase_A_M	24.03	47	2.18E-005
PF02738	Ald_Xan_dh_C2	35.72	63	2.33E-005
PF03758	SMP-30	19.35	40	2.63E-005
PF01638	DUF24	37	64	3.51E-005
PF01757	Acyl_transf_3	54.86	87	3.75E-005
PF00067	p450	24.24	46	5.31E-005
PF02746	MR_MLE_N	50.18	80	6.30E-005
PF02894	GFO_IDH_MocA_C	66.35	100	6.97E-005
PF01799	Fer2_2	31.26	55	7.69E-005
PF06169	DUF982	11.06	26	8.88E-005
PF07536	HWE_HK	23.82	44	1.35E-004
PF01022	HTH_5	68.47	101	1.38E-004
PF03573	OprD	14.03	30	1.41E-004
PF00656	Peptidase_C14	14.89	31	1.73E-004
PF03459	TOBE	83.78	139	2.48E-004
PF02627	CMD	51.89	79	2.75E-004
PF01188	MR_MLE	56.78	85	2.79E-004
PF07506	RepB	10.84	24	3.81E-004
PF01261	AP_endonuc_2	85.48	122	4.91E-004
PF00150	Cellulase	11.06	24	4.97E-004
PF01408	GFO_IDH_MocA	85.7	130	5.36E-004
PF00941	FAD_binding_5	21.05	38	5.58E-004
PF01315	Ald_Xan_dh_C	29.35	49	5.60E-004
PF00353	HemolysinCabind	36.15	57	8.12E-004
PF06823	DUF1236	8.93	20	9.64E-004

The domain with the statistically most significant over-representation in the plant-associated bacteria was the guanylate cyclase domain (Pfam:PF00211). This domain was particularly abundant in *B. japonicum *(32 proteins) and S. *meliloti *(24 proteins). No other fully-sequenced proteobacterium encodes more than three, although the spirochaete *Leptospira interrogans *encodes 17 proteins matching PF00211). Cyclic-diGMP, the product of guanylate cyclase, is a secondary messenger that plays a role in cell-cell and cell-surface contact in several bacteria by regulating cellular adhesion genes [33]. Such interactions are very important in initiating bacterial infection of eukaryotic organisms and this may account in part for the high numbers of such domains in these plant-associated bacteria. Of particular interest is the observation that one response regulator from *C. crescentus *has been shown to become sequestered to the cell pole following phosphorylation [35]. This is coupled to the activation of the guanylate cyclase domain, suggesting that localised synthesis of this secondary message could induce local effects within specific regions of the bacterial cell.

Another domain with statistically significant over-representation in the plant-associated bacteria was the bacterial luciferase-like monooxygenase domain (Pfam:PF00296). This domain was particularly abundant in the plant-associated alpha-Proteobacteria with 15 proteins in *Agrobacterium tumefaciens*, 11 proteins in *B. japonicum *and 9 proteins in *M. loti *containing this domain. The related alpha-Proteobacteria *C. crescentus*, *B. melitensis*, *B. suis *and *Rhodopseudomonas palustris *have 3, 2, 2 and 0 luciferase (PF00296) proteins respectively. Other species containing large numbers of luciferase-like proteins include *Mycobacterium bovis *(13 proteins) and *M. tuberculosis *(14 proteins).

Several domains of unknown function are amongst those most over-represented in the phytobacteria. For example, DUF636 is unusually abundant in the rhizobia with 16 representative proteins in *B. japonicum *and 14 and 13 in *M. loti *and *S. meliloti *respectively. Other prokaryotes encode between 0 and 5 DUF636 proteins, whilst *Arabidopsis thaliana *and *Homo sapiens *each encode one.

### Domain architectures

The functionality of the proteome depends not only on the repertoire of protein domains but also on the interactions and cellular context of those domains. One important aspect of this context is the range of combinations of domains within a protein; that is the domain architecture of proteins.

We used the Pfam database to ascertain the domain architecture of every protein sequence from each bacterial species for which a complete annotated genome sequence was available. 3,774 distinct protein domain architectures were found in *R. solanacearum*, *P. aeruginosa*, *E. carotovora *(subspecies *atroseptica*), *P. syringae *(pathovar *tomato*), *B. japonicum*, *S. meliloti*, *M. loti*, *A. tumefaciens*, *X. fastidiosa*, *X. campestris*, *X. axonopodis*. 459 of the 3,774 domain architectures encoded in genomes of plant-associated bacteria were absent in all other bacteria for which complete genome sequences were available. These 459 architectures are listed in the supplementary data. However, many of these architectures were restricted to a single species or several closely related species and so were of limited interest for this study.

We were particularly interested to discover whether any domain architectures are related to plant-associated lifestyle rather than simply resulting from phylogeny. The 15 protein architectures illustrated in Table 4 were each found in plant-associated bacteria from at least two different divisions of the Proteobacteria and were not found in any other non-plant-associated organisms. For example, polypeptide sequences consisting of an N-terminal domain of unknown function DUF442 fused to a metallo-beta-lactamase domain are restricted to *A. tumefaciens*, *M. loti*, *S. meliloti*, *X. fastidiosa and X. fastidiosa*.The metallo-beta-lactamase domain (Pfam:PF00753) is common and widespread, being found in over 2000 different proteins from a wide range of organisms. However, only in these proteins from plant-associated bacteria is the metallo-beta-lactamase domain fused to DUF442. This suggests that the catalytic domain may have been recruited to some new function connected to a plant-associated lifestyle in these bacteria.

**Table 4:** Domain architectures found in phytobacteria of two or more subdivisions of the Proteobacteria and not found in non-plant-associated bacteria.

**Domain architecture**	***Species distribution***	**Proteins**
**DUF763**	*Aeropyrum pernix; Archaeoglobus fulgidus; Bradyrhizobium japonicum; Methanobacterium thermoautotrophicum; Methanopyrus kandleri; Picrophilus torridus; Pyrobaculum aerophilum; Pyrococcus abyssi; Pyrococcus furiosus; Pyrococcus horikoshii; M. loti; S. meliloti; Sulfolobus solfataricus; Sulfolobus tokodaii; Thermoplasma acidophilum; Thermoplasma volcanium; X. axonopodis (pv. citri); X. campestris (pv. campestris);*	Hypothetical protein XCC1094. (Q8PBM5); Hypothetical protein XAC1190. (Q8PN83); Hypothetical protein APE1824. (Q9YAX1); Hypothetical protein ST0586. (Q974S6); Hypothetical protein PF0611. (Q8U361); Hypothetical protein. (Q97VZ2); Hypothetical protein PH0745. (O58515); Hypothetical protein SMb21455. (Q92U57); Hypothetical protein. (Q9UZ46); Mlr6856 protein. (Q987Y3); Bll3834 protein. (Q89NK4); Uncharacterized conserved protein. (Q8TYA4); Hypothetical protein PAE0766. (Q8ZYH9); Hypothetical protein TVG0468151. (Q97BH6); Hypothetical protein Ta1095. (Q9HJ77); Hypothetical protein AF1496. (O28776); Hypothetical protein. (Q6L1J8); Hypothetical protein MTH448. (O26548); Hypothetical protein MTH449. (O26549);
VirK	*A. tumefaciens (strain C58 / ATCC 33970); A. tumefaciens; Bradyrhizobium japonicum; P. syringae (pv. tomato); R. solanacearum; Rhizobium sp. (strain NGR234); X. axonopodis (pv. citri); X. campestris (pv. campestris); X. fastidiosa (strain Temecula1 / ATCC 700964); X. fastidiosa;*	VirK (Tiorf135 protein). (O50246*); VirA/G regulated gene. (Q7CNV8); Hypothetical 15.8 kDa protein in pinF2 3'region (ORF2). (Q44433*); Hypothetical 15.6 kDa protein y4WH. (P55686*); PUTATIVE SIGNAL PEPTIDE PROTEIN. (Q8XX33*); VirK protein. (Q8PDC2*); VirK protein. (Q8PQ93); ID299. (Q9ANE2*); Blr1847 protein. (Q79UP9); VirK protein. (Q87D31); VirK protein. (Q9PC40*); Hypothetical protein. (Q880Z8);
DUF1427	*A. tumefaciens (strain C58 / ATCC 33970); Bradyrhizobium japonicum; P. aeruginosa; R. solanacearum; Rhizobium leguminosarum (biovar trifolii); S. meliloti; X. campestris (pv. campestris);*	Hypothetical protein XCC2052. (Q8P914); Bsl6958 protein. (Q89EW2); Hypothetical protein. (Q93EB2); HYPOTHETICAL TRANSMEMBRANE PROTEIN. (Q8Y2U1*); AGR_L_1747p. (Q8U4X9*); Hypothetical protein. (Q92Y85); Bsr4258 protein. (Q89MD5); Hypothetical protein. (Q9I0E5*);
DUF1486	*A. tumefaciens (strain C58 / ATCC 33970); Neurospora crassa; P. aeruginosa; P. syringae (pv. tomato); R. solanacearum; M. loti; S. meliloti;*	Hypothetical protein. (Q7SFH5); Hypothetical protein Atu3018. (Q8UBJ8); Hypothetical protein. (Q92YL1); Mlr2224 protein. (Q98IW1); Hypothetical protein. (Q9I3U3); Hypothetical protein. (Q9JP27); AGR_L_3571p. (Q7CRD4); Hypothetical protein RSc0819. (Q8Y171);
RepB	*A. tumefaciens (strain C58 / ATCC 33970); P. syringae (pv. tomato); M. loti; S. meliloti;*	Msr9757 protein. (Q98P91); Mll8115 protein. (Q983Y2); Hypothetical protein. (Q88BH6); Hypothetical protein Atu5040. (Q8UKR0); AGR_pAT_52p. (Q7D423); Hypothetical protein. (Q92XS2); Hypothetical protein. (Q930E6); Hypothetical protein. (Q930E5);
DUF442~Lactamase_B	*A. tumefaciens (strain C58 / ATCC 33970); M. loti; S. meliloti; X.fastidiosa (strain Temecula1 / ATCC 700964); X. fastidiosa;*	Metallo-beta-lactamase superfamily protein. (Q8UAA9); Hypothetical protein. (Q92ZB8); AGR_L_2726p. (Q7CSJ2); Hypothetical protein. (Q87AD6); Mlr2158 protein. (Q98J12); Hypothetical protein. (Q9PFB0);
GAF~Phytochrome	*Bradyrhizobium sp. ORS278; X. axonopodis (pv. citri);*	Phytochrome-like protein. (Q8PEQ2); Bacteriophytochrome. (Q8VUB6);
Glyco_hydro_6~CBM_2	*Microbispora bispora; Micromonospora cellulolyticum; R. solanacearum; Thermomonospora fusca; X. fastidiosa (strain Temecula1 / ATCC 700964); X. fastidiosa;*	Cellulose 1,4-beta-cellobiosidase. (Q87E00); 1,4-beta-cellobiosidase. (Q9PDW2); PROBABLE EXOGLUCANASE A (1,4-BETA-CELLOBIOSIDASE) PROTEIN (EC3.2.1.91). (Q8XS97); Endoglucanase A precursor (EC 3.2.1.4) (Endo-1,4-beta-glucanase) (Cellulase). (P26414*); Endoglucanase E-2 precursor (EC 3.2.1.4) (Endo-1,4-beta-glucanase E-2)(Cellulase E-2) (Cellulase E2). (P26222*); Endo-beta-1,4-glucanase. (Q53488);
DUF811	*P. aeruginosa; R. solanacearum;*	Hypothetical protein. (Q9I6E4*); Hypothetical protein. (Q9I6E5*); Hypothetical protein RSc3082. (Q8XUV1);
Condensation~Condensation~AMP-binding~PP-binding~Condensation~AMP-binding~PP- binding~Condensation~AMP-binding~PP- binding~Condensation~AMP-binding~PP- binding~Condensation~AMP-binding~PP- binding~Thioesterase~Thioesterase	*P. syringae (pv. tomato); R. solanacearum;*	Probable peptide synthesis protein. (Q8XS39); Non-ribosomal peptide synthetase, terminal component. (Q881Q3);
NolX	*R. solanacearum; Rhizobium fredii (Sinorhizobium fredii); M. loti; Rhizobium sp. (strain NGR234); X. axonopodis (pv. citri); X. axonopodis pv. glycines; X. campestris (pv. campestris); X. campestris (pv. vesicatoria); X. oryzae (pv. oryzae);*	HrpF protein. (Q8PBA6); HrpF protein. (Q8PQD2); HrpF. (Q83XD5); HrpF. (O33967); HrpF. (Q6F5A9); HrpF. (Q9KW22); Type III secretion system component. (Q6QJ83); SECRETED PROTEIN POPF2. (Q8XRF4); SECRETED PROTEIN POPF1. (Q8XPT2); Nodulation protein; NolX. (Q989P8); Nodulation protein nolX. (P55711); Nodulation protein NolX. (Q93LZ2); Nodulation protein NolX. (Q9EUG7); Nodulation protein nolX. (P33213);
DUF802~DUF802	*R. solanacearum; X. axonopodis (pv. citri);*	Hypothetical protein XAC3753. (Q8PG64*); Probable transmembrane protein (Q8XQ05*);
Avirulence~Avirulence	*R. solanacearum; X. axonopodis (pv. citri); X. campestris (pv. citri); X. campestris (pv. vesicatoria); X. campestris; X. oryzae (pv. oryzae); X. oryzae;*	Avirulence protein AvrXa7-3M. (Q6GWX1); Avirulence protein AvrXa7-1M. (Q6GWX7); Avirulence protein. (Q9EZV3); Avirulence protein AvrXa7-4M. (Q6GWX4); Avirulence protein. (Q9F0D0); Hypothetical 122 kDa avirulence protein in avrBs3 region. (P14727); AvrBs3-2 protein. (Q07061); PROBABLE AVRBS3-LIKE PROTEIN. (Q8XYE3); Apl3 protein. (Q9Z3F5); Avirulence protein. (Q8PRG7); PthA protein. (Q56780); Apl1 protein. (Q9R7J3); Avirulence protein AvrXa7-2M. (Q6GWX3); Avirulence protein. (Q8PRN6); Avirulence protein AvrXa10. (Q56830); PthB. (Q7X130); Apl2 protein. (Q9Z3F6); Avirulence protein. (Q8PRM3); Avirulence protein. (Q8PRK7);
RgpF-RgpF	*M. loti; Rhizobium sp. (strain NGR234); X. axonopodis (pv. citri); X. campestris (pv. campestris);*	Mll4799 protein. (Q98D97); Hypothetical protein XAC3576. (Q8PGP0); Hypothetical protein wxcX. (O34262); Hypothetical 45.0 kDa protein y4gN. (P55470);
TPR_2~TPR_1~Sulfotransfer_1	*M. loti; X. axonopodis (pv. citri); uncultured bacterium 560;*	TPR domain/sulfotransferase domain protein. (Q6SGF7); Mlr4028 protein. (Q98EY4); Hypothetical protein XAC3051. (Q8PI47);

One regulatory domain found in large numbers in *Pseudomonas *genome is the PAS domain (Pfam PF00989) [36], which is present in 25 ORFs in *P. aeruginosa *PAO1 and 30 ORFs in *P. syringae *pathovar *tomato*. The average number of PAS-containing ORFs in complete proteobacterial genomes is about 10. Although PAS domains are only found in a limited subset of bacterial regulators, they are at the forefront of molecular innovation with 9 of the novel architectures identified in *P. aeruginosa*, and 5 of those in *P. syringae *pathovar *tomato *containing PAS domains (see supplementary data for more details). *Xanthomonas *genomes also encode a large number of PAS-containing polypeptides, (18 and 21 in X. *axonopodis *and X. *campestris *respectively). However, each *X. fastidiosa *encodes only one: PhoR, a regulator generally associated with responses to phosphate limitation. Ten novel PAS architectures are present in each *Xanthomonas *genome, of which 7 are common and 3 are unique to each strain (some of which are illustrated in Figure 3). PAS domains, which are involved in sensing light, oxygen and other environmental factors, have particular importance in helping bacteria to adapt to a changing environment, an ability of little value to *X. fastidiosa *in its restricted and relatively constant niches.

**Figure 3:**
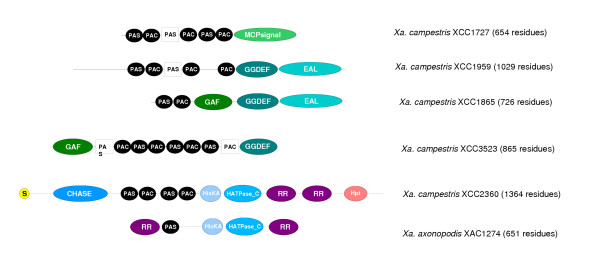
Examples of proteins containing phytochrome domains.

**Figure 4:**
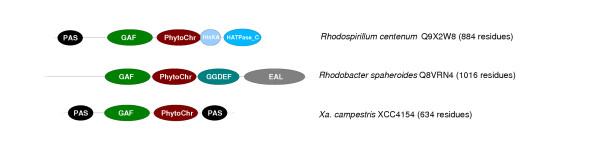
Examples of proteins containing phytochrome domains.

One intriguing signal transduction domain identified in unique domain architectures from both *P. syringae *and *Xanthomonas *was a phytochrome domain (Pfam:PF00360) (Figure 4). This domain enables light-mediated signal transduction in plants and bacteria, through binding a light-sensitive chromophore [37,38]. Phytochrome-containing proteins are used to detect light, and to discriminate between different wavelengths of light. Phytochromes are used for shade avoidance by plants, and to detect depth in soil or water or other conditions where light is attenuated. The short list of bacteria that contain phytochromes includes photosynthetic species (*e.g*. *Rhodospirillum centenum*, *Anabaena *species strain PCC7120 and *Synechocystis *species strain PCC6803) as well as plant associated bacteria (*e.g*. *R. leguminosarum*, *A. tumefaciens*) and soil bacteria (*e.g. P. putida*) [38,39]. An unusual photosynthetic strain, *Bradyrhizobium *species ORS278 uses phytochrome to regulate the photosynthesis gene cluster and a similar induction was seen with *Rhodopseudomonas pallustris *but not with several other photosynthetic bacteria [40]. It is not known why phytochrome proteins are retained in non-photosynthetic bacteria but it has been suggested that the phytochrome-like sensor kinases in *Agrobacterium *may play a role in detecting depth in soil strata as a means of optimising interactions with roots [39]. Most of the bacterial phytochrome proteins have a PAS domain and a GAF domain at the N-terminus and a histidine kinase domain at the C-terminus (see Figure 4), though a phytochrome from *Rhodobacter sphaeroides *(UniProt:Q8VRN4; see Figure 4) has a more complex domain architecture [40]. The presence of two phytochromes in *P. syringae*, one of them with a unique architecture, may reflect the recruitment of phytochrome to a novel regulatory function unique to *P. syringae*. Protein PSPTO2652 from *P. syringae *is unique in that it has an additional C-terminal histidine kinase. Another unusual domain architecture is the PAS-GAF-Phytochrome-PAS organisation found in *Xanthomonas *proteins XAC4293 and XCC4154 (Figure 4), which, if shown to be functional, may represent a new phytochrome protein family.

### Further analysis of novel *Pseudomonas *protein domain architectures

The availability of multiple finished and unfinished *Pseudomonas *genomes allowed us to study in more detail the distribution, genomic context and properties of *Pseudomonas gene products *highlighted by this analysis. Closer examination of the genomic context of the *P. syringae *genes encoding proteins with unusual domain architectures showed that most were flanked on either or both sides by genes that have few or no orthologues in other *Pseudomonas *strains, suggesting that these novel genes have been recruited simultaneously with other genes, possibly of related function, or that they have recombined into the genome at hotspots for recombination and insertion of alien DNA.

To further address the hypothesis that at least some of these architectures have been acquired by horizontal gene transfer we examined the GC content and third position GC content of each of these genes, in comparison to the total genome (0.593 GC, 0.716 GC3). Sixteen of the genes deviated from the average GC3 content by more than 0.05. High GC3 content genes include *pvsA*, PSPTO4084, PSPTO2413 and *cfa6*. Low GC3 content genes include *hrpZ*, PSPTO3210, *glf*, PSPTO4696, *hopPtoS*(1,2 & 3), PSPTO2259, PSPTO0400, *avrF *and PSPTO1070. The GC content of flanking genes frequently reflected that of the novel gene, most strikingly for *glf*, PSPTO2441, PSPTO4696, *hopPtoS*(1,2 &3), PSPTO4699, PSPTO1070 & PSPTO2632, which were each associated with low GC regions containing few ORFs with orthologues in other *Pseudomonas *genomes.

**Figure 5:**
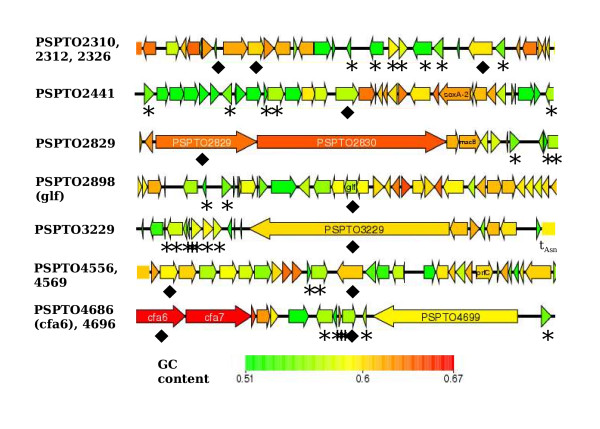
**Genetic islands unique to *Pseudomonas syringae***. Genes encoding transposases are marked with an asterisk (*) and the asparaginyl tRNA gene is marked 'tAsn'. Black diamonds indicate genes encoding unique domain architectures [49].

One other feature frequently associated with horizontally transferred genes is the presence of IS elements, tRNAs, plasmid and phage genes in flanking regions. PSPTO3229, PSPTO4569, PSPTO2312, PSPTO2829, PSPTO2310, Glf, PSPTO2441, PSPTO4696 and PSPTO2326 are all located in close proximity to IS elements and phage-like sequences, or in defined regions of the genome flanked by IS elements and phage-like sequences (see Figure 5).

Overall, this analysis suggests that a large number of the novel architectures present in *P. syringae pathovar. tomato *are uniquely associated with this species or pathovar of *Pseudomonas*, and that many of these genes have been acquired by horizontal gene transfer and are located in regions of the genome with a high potential for recombination and rearrangement.

## Conclusions

Our initial observations, from the clustering of complete prokaryotic proteomes on the basis of domain content, motivated us to test whether any protein domains or domain architectures are specifically associated with a plant-associated lifefstyle. We identified nine protein domain families that are found in phylogenetically diverse plant-associated bacteria but not in non-plant-associated Bacteria (Table 1). Inevitably, there is an element of random chance in the species distribution of domain families; however, we observed that most of domains whose functions are at least partly known are implicated in the plant associated lifestyle. Therefore it seems possible that the two domains of unknown function (DUF811 and DUF1427) may also turn out to be significant for this lifestyle. Several domain families were also found only in plant pathogenic bacteria and in eukaryotes (Table 2). For example the RolB/RolC-like domain family is restricted to plant-associated bacteria and to plants of the genus *Nicotiana*, and is implicated in modulating auxin activity.

Having investigated patterns of presence or absence of domains within bacterial proteomes, we next identified which domains are most over-represented in the plant-pathogenic Proteobacteria as compared with the frequency of occurrence in all the sequenced Proteobacteria (Table 3). Amongst the most over-represented domains was the guanylate cyclase domain. This was largely due to the large number of guanylate-cyclase-like proteins encoded by *B. japonicum *and *S. meliloti*. Although this approach may have revealed some potential leads for further investigation, it should be remembered that this analysis was rather crude and susceptible to the biased phylogenetic distribution of the organisms for which complete genome sequence data are currently available. However, detailed analysis of the frequency distributions of protein domain families in various organisms may yield rewards.

As well as the repertoire of domains, another important aspect of a proteome is the repertoire of domain architectures; that is the combinations of domains found within a single protein. Just as for the repertoire of domains, the species distribution of a domain architecture might be explained by chance. Nevertheless, the proteins listed in Table 4 may be a good starting point for further investigation of bacterium-plant interactions.

Many of these protein identified in this study have N-terminal predicted signal peptide motifs, suggesting that they are secreted. Further experiments are required to determine whether proteins of unknown function will also have a role in plant-specific functions. Many proteins involved in bacteria-plant interactions, such as TTSS-secreted effectors have subtle or conditional phenotypes, and would not be identified in conventional mutant-phenotype screens. Assays to detect subtle differences in growth *in planta *or in disease development are labour-intensive. Bioinformatic analyses such as this one represent useful and informative tools for reducing experimental search space, particularly when combined with other post-genomic techniques such as microarray analyses.

We found relatively little evidence of lateral dissemination of niche-specific novel architectures between phylogenetically distinct divisions in the Proteobacteria, with less than 20 phytobacteria-specific domain architectures present in two or more divisions of the Proteobacteria. We did identify a number of domain architectures and domains that were uniquely conserved in both plant-associated prokaryotes and eukaryotes. The methodology used in this study makes no prior assumptions about the nature or cause of "uniqueness". Unique architectures identified using this approach include rare domains, novel domain combinations and architectures that are truncated relative to the majority of similar proteins (which may represent deletions and loss of function mutations). Some proteins will inevitability be included or excluded because of the limitations of current domain prediction technology. However, in addition to identifying protein candidates for further investigation, this type of analysis can be used to challenge and improve current models for domain prediction and expose errors and limitations of genome sequence data and protein prediction. For example, consider a case in which a protein is identified as having the "unique" architecture B~C~D. Additional examination of the protein may reveal that the protein has a similar sequence to proteins with the architecture A~B~C~D. The absence of the A domain may indicate a genuine alteration in structure and potentially in function, or a frameshift in the genome sequence data, or a functional "A" domain that fails to meet current predictive criteria. Each of these hypotheses can be tested by further research and experimentation, both *in silico *and in the lab.

Although our approaches to identifying candidate genes and proteins of significance to lifestyle have led to several potential leads and interesting hypotheses, there are some caveats. Firstly, evolution does not proceed exclusively through loss and gain of domains and domain shuffling; for example, protein innovation can also occur through mutation and divergence within domain families. Also, it is becoming increasingly apparent that an organism's physiology, behaviour and ecology depend as much on higher order 'systems level' phenomena as on the inventory of molecular components.

We chose to base our surveys of protein domains on the Pfam because this mature database is relatively comprehensive in its coverage (*e.g*. compared with SMART) and its data is of high quality. Furthermore, its data is distributed in a form that is ideally suited for constructing database queries such as those in this study. Another advantage is that in Pfam no two domains ever overlap in their coverage of a protein sequence, which significantly simplifies the analysis. However, it should be noted that Pfam is not absolutely infallible and some of its threshold values are rather stringent, leading to failure to identify some 'outlying' members of a domain family.

In summary, this study has described and applied a new approach for identifying architectural innovation and potentially important domains in proteins from genome sequence data. The data generated in this study have highlighted a large number of interesting and largely uncharacterised novel proteins and suggested new insights into the molecular basis of interactions between bacteria and their plant hosts, which will provide inspiration for future experimental research.

## Methods

The Pfam relational database data files were downloaded from the Pfam website [46]. The census of domains and architectures were taken from Pfam release 16.0 (November 2004) using custom PERL scripts to wrap SQL queries against the Pfam relational database.

The complete bacterial genomes included in Pfam 16.0, and hence considered in this study, are listed in the supplementary data. We excluded from the analysis of domain architectures all protein sequences in UniProt [47] that are designated as fragments.

A file listing the presence or absence of each Pfam domain in each proteome can be found in the supplementary data. Each row in this file represented a vector used for the clustering of bacterial proteomes. Neighbour-joining was performed using PHYLIP [41]. Trees were visualised using ATV [51].

BLAST [42] searches were performed using the NCBI [48] and Expasy [49] web servers. Comparison between *Pseudomonas *genomes was aided by use of PseudoDB [50]. Transmembrane and signal peptide predictions were taken from Pfam, which in turn uses TMHMM [45] and SignalP [43]. It should be remembered that predictive methods often have difficulty distinguishing between signal peptides and N-terminal transmembrane helices [44].

## Authors' contributions

DJS and GMP conceived the original study, carried out the bioinformatics analyses, and drafted the manuscript. JAD proposed extending the study to symbionts as well as pathogens. All the authors contributed to interpretation of the data and to writing the final manuscript.

## Supplementary Material

Additional File 1 This table lists the 459 domain architectures that are found in one or more plant-associated bacteria but are absent from other bacteria for which complete sequence data is available. Click here for file

Additional File 2 Prokaryotic genomes included in Pfam16.0 (and hence in this study). Click here for file

Additional File 3 "domains.tab.gz" Species distribution of each of the 3,774 Pfam domains. This tab-delimited file has been compressed using gzip. Click here for file
