# Supplementary material for: Protein domains and architectural innovation in plant-associated Proteobacteria
Source: BMC Genomics. 2005 Feb 16;6:17. doi: 10.1186/1471-2164-6-17 (PMC554113; doi:10.1186/1471-2164-6-17)
Supplement: Additional File 1 — This table lists the 459 domain architectures that are found in one or more plant-associated bacteria but are absent from other bacteria for which complete sequence data is available. [file 1471-2164-6-17-S1.pdf]

Domain architecture	Species distribution	Proteins (UNIPROT accession numbers). Asterisks indicate protein has a predicted N-terminal signal peptide
2-Hacid_dh	Arabidopsis thaliana (Mouse-ear cress); Fusobacterium nucleatum subsp. vincentii ATCC 49256; Homo sapiens (Human); Oryza sativa (japonica cultivar-group); M . loti; Saccharomyces cerevisiae (Baker's yeast);	ORF YPL276w. (Q08987); D-3-phosphoglycerate dehydrogenase. (Q98DF3); Putative glycerate dehydrogenase (At2g45630/F17K2.16). (O64643); D-3-phosphoglycerate dehydrogenase (EC 1.1.1.95). (Q7P6L6); Hypothetical LOC403340. (Q6P3W3); Hypothetical protein FLJ34843. (Q8NAS8); Hypothetical protein P0708B04.46 (Hypothetical proteinP0479C12.21). (Q6Z8P7); Hypothetical protein P0453E05.129 (Hypothetical proteinOJ1579_C03.11). (Q84ZJ3);
3HCDH_N~3HCDH~4HBT	A. tumefaciens ;	AGR_pAT_493p. (Q7D3B2); 3-hydroxyacyl-CoA dehydrogenase. (Q8UJY0);
AA_kinase~ACT~ACT~Orn_Arg_deC_N~Orn_DAP_Arg_deC	X. axonopodis (pv. citri); X. campestris (pv. campestris); X. fastidiosa (strain Temecula1 / ATCC 700964); X. fastidiosa;	Bifunctional diaminopimelate decarboxylase/aspartate kinase. (Q8P772); Bifunctional diaminopimelate decarboxylase/asparta. (Q8PII6); Diaminopimelate decarboxylase. (Q87EA9); Bifunctional diaminopimelate decarboxylase/aspartate kinase. (Q9PEB2);
AA_kinase~DUF619	Dictyostelium discoideum (Slime mold); X. axonopodis (pv. citri); X. campestris (pv. campestris); X. fastidiosa (strain Temecula1 / ATCC 700964); X. fastidiosa;	Acetylglutamate kinase (EC 2.7.2.8) (NAG kinase) (AGK) (N-acetyl-L-glutamate 5-phosphotransferase). (Q8P8J6); Acetylglutamate kinase (EC 2.7.2.8) (NAG kinase) (AGK) (N-acetyl-L-glutamate 5-phosphotransferase). (Q8PK29); Acetylglutamate kinase (EC 2.7.2.8) (NAG kinase) (AGK) (N-acetyl-L-glutamate 5-phosphotransferase). (Q87EL2); Acetylglutamate kinase (EC 2.7.2.8) (NAG kinase) (AGK) (N-acetyl-L-glutamate 5-phosphotransferase). (Q9PEM7); Similar to X. axonopodis (Pv. citri). Acetylglutamatekinase. (Q86A87);
ABC_tran~ABC_tran~BPD_transp_2	M . loti;	Ribose ABC transporter, ATP-binding protein. (Q987K1);
ABC_tran~UPF0261	B. japonicum;	ABC transporter permease protein. (Q89PU8);
ABM~Ni_hydr_CYTB	R. solanacearum;	HYPOTHETICAL TRANSMEMBRANE PROTEIN. (Q8XYB2);
AMP-binding~PP-binding~Condensation~AMP-binding~PP-binding~Condensation~AMP-binding~PP-binding~Condensation~Condensation~Condensation~AMP-binding~PP-binding	P. aeruginosa; P. syringae (pv. tomato);	Probable non-ribosomal peptide synthetase. (Q9I157); Pyoverdine chromophore synthetase. (Q884F8);
AMP-binding~PP-binding~Condensation~AMP-binding~PP-binding~Condensation~AMP-binding~PP-binding~Thioesterase	B. japonicum;	ID930. (Q9AMR5); Blr2108 protein. (Q79UB9);
AMP-binding~PP-binding~Condensation~AMP-binding~PP-binding~Thioesterase	Xanthomonas albilineans; X. axonopodis (pv. citri);	Non-ribosomal peptide synthase. (Q70C52); ATP-dependent serine activating enzyme. (Q8PKR7);
AMP-binding~PP-binding~ketoacyl-synt~Ketoacyl-synt_C~Acyl_transf_1~PP-binding~Aminotran_3~Bac_luciferase~Condensation~AMP-binding~PP-binding~Condensation~AMP-binding~PP-binding~Condensation~AMP-binding~PP-binding~Condensation~AMP-binding~PP-binding	R. solanacearum;	PROBABLE PEPTIDE SYNTHETASE PROTEIN. (Q8XS40);
AMP-binding~PP-binding~ketoacyl-synt~Ketoacyl-synt_C~Acyl_transf_1~PP-binding~Condensation~AMP-binding~PP-binding~Condensation~PP-binding	R. solanacearum;	PROBABLE POLYKETIDE SYNTHASE PROTEIN. (Q8XYF2);
AMP-binding~Thioesterase	M . loti;	Mll6752 protein. (Q988G5); Mll6746 protein. (Q988G9); Peptide synthetase homolog. (Q98NV6);
ANTAR~TP_methylase	X. axonopodis (pv. citri);	Uroporphyrin-III C-methyltransferase. (Q8PKL4);
ATP-grasp~CoA_binding	B. japonicum;	Blr3159 protein. (Q89QG9);
Abhydrolase_1~GerE	M . loti;	Mll5392 protein. (Q98BX2);
Abhydrolase_1~Guanylate_cyc	B. japonicum;	Bll8011 protein. (Q89BY7);
Acyl-CoA_dh_N~Acyl-CoA_dh_M~Acyl-CoA_dh~Acyl-CoA_dh	B. japonicum;	Bll7818 protein. (Q89CH9);

Acyl-CoA_dh~Acyl-CoA_dh	B. japonicum;	Blr3450 protein. (Q89PN0);
Acyl_transf_1~PP-binding~ketoacyl-synt~Ketoacyl-synt_C~Acyl_transf_1~ADH_zinc_N~PP-binding	E. carotovora subsp. atroseptica SCRI1043; P. syringae (pv. tomato); P. syringae;	Type I polyketide synthase. (Q6D9L1); Coronafacic acid polyketide synthase I. (Q87W70); Type I polyketide synthase. (Q9Z3T9);
Acylphosphatase~Sua5_yciO_yrdC~Peptidase_M22~HupF_HypC	B. japonicum;	HypF. (Q9ANP2); Hydrogenase maturation protein. (Q79UW6);
Aegerolysin	Agrocybe aegerita (Black poplar mushroom); Aspergillus fumigatus (Sartorya fumigata); Clostridium bifermentans; Neurospora crassa; Pleurotus ostreatus (Oyster mushroom) (White-rot fungus); P. aeruginosa;	Aegerolysin Aa-Pri1 precursor. (O42717); PriA. (Q8X1M9); Hypothetical protein. (Q9I710); Related to Hemolysin. (Q8WZT0*); Hemolysin-like protein. (O32337); Hemolysin-like protein. (O32338); Asp-hemolysin precursor (Asp-HS). (Q00050);
Agro_virD5	Agrobacterium rhizogenes; A. tumefaciens ; A. tumefaciens; Plasmid Ti;	VirD5. (Q8VT84); VirD5 (AA 1-841) protein. (Q44207); Tiorf159 protein. (Q9R6B6); VirA/G regulated protein. (Q8U5Y9); Hypothetical protein. (Q52603); AGR_pTi_24p. (Q7D2T1); VirD5. (Q9JN11); Riorf171 protein. (Q9F584);
Ala_racemase_N~Arginase	M . loti;	Mlr5700 protein. (Q98B74);
Ald_Xan_dh_C~Ald_Xan_dh_C2~Ald_Xan_dh_C2~COXG	B. japonicum;	CutL protein. (Q89PE9);
Aldedh~Aldedh	A. tumefaciens ; Homo sapiens (Human); M . loti; S. meliloti;	AGR_L_75p. (Q7CW12); NADP-dependent aldehyde dehydrogenase. (Q8U6F8); Putatively membrane-anchored aldehyde dehydrogenase protein (EC1.2.1.3). (Q92VA3); Aldehyde dehydrogenase. (Q98DB0); Hypothetical protein MGC10204. (Q8IZ83);
Anthrax_toxA	P. aeruginosa;	Adenylate cyclase ExoY. (Q9I1S4); Adenylate cyclase. (O85345);
Arg_tRNA_synt_N~tRNA-synt_1d	B. japonicum;	Arginyl-tRNA synthetase. (Q89KZ1);
Asp_Arg_Hydrox~Pro_3_hydrox_C	M . loti; S. meliloti; Streptomyces sp; Streptomyces sp. TH1;	L-proline 3-hydroxylase. (P96010); L-proline 3-hydroxylase type II. (O09345); PUTATIVE L-PROLINE 3-HYDROXYLASE PROTEIN. (Q92LF6); L-proline 3-hydroxylase. (Q989T9);
Autoind_bind	Agrobacterium rhizogenes; A. tumefaciens ; A. tumefaciens; Oligotropha carboxidovorans (Pseudomonas carboxydovorans); Proteus vulgaris; Rhizobium leguminosarum (biovar viciae);	Transcriptional regulator. (Q8L2C1); Riorf119 protein. (Q9F5D3); TrlR. (O69215); TraR. (Q84G43); Regulation protein RhiR. (Q6LBC3); Quorum sensing TraR agonist. (Q8U5Y6); AGR_pTi_32p. (Q7D2S8);
Avidin	B. japonicum; Gallus gallus (Chicken); Streptomyces avidinii; Streptomyces violaceus (Streptomyces venezuelae);	Avidin precursor. (Q98SH4*); Avidin precursor. (P02701*); Streptavidin precursor. (P22629*); Streptavidin V2 precursor (SA V2). (Q53533*); Streptavidin V1 precursor (SA V1). (Q53532*); Avidin-related protein 1 precursor. (O13153*); Avidin-related protein 7 precursor. (P56736*); Avidin-related protein 6 precursor. (P56735*); Avidin-related protein 4/5 precursor. (P56734*); Avidin-related protein 3 precursor. (P56733*); Avidin-related protein 2 precursor. (P56732*); Blr5658 protein. (Q89IH6);
Avirulence~Avirulence	R. solanacearum; X. axonopodis (pv. citri); X. campestris (pv. citri); X. campestris (pv. vesicatoria); X. campestris; Xanthomonas oryzae (pv. oryzae); Xanthomonas oryzae;	Avirulence protein AvrXa7-3M. (Q6GWX1); Avirulence protein AvrXa7-1M. (Q6GWX7); Avirulence protein. (Q9EZV3); Avirulence protein AvrXa7-4M. (Q6GWX4); Avirulence protein. (Q9F0D0); Hypothetical 122 kDa avirulence protein in avrBs3 region. (P14727); AvrBs3-2 protein. (Q07061); PROBABLE AVRBS3-LIKE PROTEIN. (Q8XYE3); Apl3 protein. (Q9Z3F5); Avirulence protein. (Q8PRG7); PthA protein. (Q56780); Apl1 protein. (Q9R7J3); Avirulence protein AvrXa7-2M. (Q6GWX3); Avirulence protein. (Q8PRN6); Avirulence protein AvrXa10. (Q56830); PthB. (Q7X130); Apl2 protein. (Q9Z3F6); Avirulence protein. (Q8PRM3); Avirulence protein. (Q8PRK7);
AvrB_AvrC	P. syringae (pv. glycinea); P. syringae (pv. syringae); P. syringae; X. campestris (pv. campestris);	Hypothetical protein. (Q7X1Q9); Avirulence protein. (Q8P8X2); Avirulence C protein. (P13836); Avirulence protein. (Q52378); Hypothetical protein. (Q9L6V6); Avirulence B protein. (P13835); Hypothetical protein. (Q79NM6);
BNR~HIM~BNR~HIM~BNR~HIM~BNR~HIM~BNR~HIM~BNR~HIM~BNR~HIM~BNR~HIM~BNR~HIM~BNR~HIM~BNR~HIM~BNR~HIM~BNR~HIM~BNR~HIM~BNR~HIM~BNR~HIM~BNR~HemolysinCabind	E. carotovora subsp. atroseptica SCRI1043;	Putative hemagglutinin/hemolysin-related protein. (Q6D230);

BPD_transp_2~ABC_tran~ABC_tran	B. japonicum; uncultured bacterium 582;	Branched-chain amino acid ABC transporter, ATP-binding/permeaseprotein. (Q6SEX8); ABC transporter ATP-binding/permease protein. (Q89P22);
BPD_transp_2~Str_synth	B. japonicum; M . loti;	Permease protein of sugar ABC transporter. (Q98JY6); ABC transporter permease protein. (Q89CC8); ABC transporter permease protein. (Q89GL9);
BTAD~Abhydrolase_1	B. japonicum;	Bll5740 protein. (Q89I99);
BTAD~TPR_4~TPR_2	M . loti;	Probable regulatory protein. (Q98IE9);
B_lectin~B_lectin	Arisaema heterophyllum; Arisaema lobatum; Arum maculatum (Cuckoo-pint); Crocus sativus; Crocus vernus; Pinellia pedatisecta; Pinellia ternata; Pseudomonas sp. BW11M1; Tulipa hybrid cultivar; X. axonopodis (pv. citri);	Hypothetical protein XAC0868. (Q8PP20*); Putidacin L1. (Q8GEJ9); Complex specificity lectin precursor. (Q41622*); Mannose-binding lectin. (Q9FVA1*); Mannan-binding lectin. (Q9AT49*); Mannose-binding lectin. (Q9FV99*); Mannose-binding lectin ALA. (Q6Q5X6); Lectin precursor. (Q38731*); Mannose-binding lectin AHA. (Q7XAT9*); Agglutinin. (Q7Y0G6*); Mannose-binding lectin. (Q6SRZ4); Mannose-binding lectin. (Q6SRZ3); Lectin precursor. (Q38730*); Lectin. (Q6Y1R9);

Bcl-2	African swine fever virus (isolate Malawi Lil 20/1) (ASFV); African swine fever virus (strain BA71V) (ASFV); African swine fever virus (strain E-70 / isolate MS44) (ASFV); Ateline herpesvirus 3; Bos taurus (Bovine); Brachydanio rerio (Zebrafish) (Danio rerio); Callitrichine herpesvirus 3; Canarypox virus; Canis familiaris (Dog); Cercopithecine herpesvirus 15 (Rhesus Epstein Barr virus); Coturnix coturnix japonica (Japanese quail); Drosophila melanogaster (Fruit fly); Felis silvestris catus (Cat); Fowlpox virus (FPV); Fowlpox virus (isolate HP-438[Munich]); Gallus gallus (Chicken); Geodia cydonium (Sponge); Herpesvirus papio; Homo sapiens (Human); Human herpesvirus 8 (HHV-8) (Kaposi's sarcoma-associated herpesvirus); Macaca mulatta rhadinovirus 17577; Meleagrid herpesvirus 1 (herpesvirus of turkeys); Murid herpesvirus 4 (MuHV-4) (Murine gammaherpesvirus 68); Mus musculus (Mouse); Pongine herpesvirus 1; P. aeruginosa; Rattus norvegicus (Rat); Rhesus monkey rhadinovirus H26-95; Saimiriine herpesvirus 2 (strain 11) (SaHV-2) (Herpesvirus saimiri); Saimiriine herpesvirus 2; Suberites domuncula (Sponge); Sus scrofa (Pig); Xenopus laevis (African clawed frog); Xenopus tropicalis (Western clawed frog) (Silurana tropicalis);	Apoptosis regulator Bcl-B (Bcl-2-like 10 protein) (Anti-apoptoticprotein NrH). (Q9HD36); Hypothetical protein. (Q6DC66); BCL2L10. (Q99M66); Pro-apoptotic Bcl-2/Bax-like protein. (Q7KJ42); AT16536p (CG8238-PA). (Q8T8Y5); Drob-1 (BG1) (Bcl-2 ortholog dBorg-1) (CG33134-PA) (Proapoptotic Bcl-2homolog DEBCL). (Q9V9C8); Bcl-2 family member protein. (Q7KM33); A1 splice variant. (Q86W13); A1-b protein. (O55177); BHRF1. (Q8UZJ4); Bcl2a1a protein. (Q8K164); Bcl-2-related protein A1 (BFL-1 protein) (Hemopoietic-specific earlyresponse protein) (A1-A). (Q07440); Bcl-2-related protein A1 (BFL-1 protein) (Hemopoietic-specific earlyresponse protein) (GRS protein). (Q16548); B-cell leukemia/lymphoma 2 related protein A1d. (O55179); BCL2-related protein A1. (Q925A9); Protein A1. (Q9W6F2); Bcl-2-related ovarian killer protein. (O88857); Bcl-2-related ovarian killer protein. (Q9DGJ5); Similar to BCL2-related ovarian killer. (Q7T381); Bcl-2-related ovarian killer protein-like (Apoptosis activatorMtd). (O35425); BCL-2-related ovarian killer protein (BOK protein). (Q9UMX3); Nr13. (Q8UWD5); Bcl-2-related ovarian killer protein. (Q792S6); Bcl-2 related ovarian killer. (Q9UL32); Bcl-2-related ovarian killer protein. (Q9I8I2); VBcl-2. (Q9Q5K8); Mcl1b. (Q8UWD6); Cell death regulator Mcl-1a. (Q9I9N3); Myeloid cell leukemia protein 1. (Q95KR3); BHRF1. (Q9IHR2); Bcl-2 homolog (BHRF1). (Q9WGB5); EAT/MCL-1 protein (Myeloid cell leukemia sequence 1). (P97287); Mus musculus adult male colon cDNA, RIKEN full-length enrichedlibrary, clone:9030625M01 product:weakly similar to APOPTOSISREGULATOR BCL-G LONG FORM (Mus musculus adult male stomach cDNA, RIKENfull-length enriched library, clone:2210008O09 product:weakly similarto APOPTOSIS REGULATOR BCL-G LONG FORM) (Mus musculus adult maletestis cDNA, RIKEN full-length enriched library, clone:4933405K19product:weakly similar to APOPTOSIS REGULATOR BCL-G LONG FORM). (Q9CPT0); BAK protein. (Q9JK59); Mus musculus NOD-derived CD11c +ve dendritic cells cDNA, RIKEN full-length enriched library, clone:F630041J23 product:BCL2-antagonist/killer 1, full insert sequence. (Q8C264); Mcl-1. (Q8HYS5); Mcl-1 protein. (Q9Z1P3); Bcl2l14 protein. (Q8R141); Apoptosis regulator NR-13. (Q90343); Induced myeloid leukemia cell differentiation protein Mcl-1. (Q07820); Pro-apoptotic protein BAKM variant. (Q8NFF3); Apoptosis regulator BCL-G long form (BCL2-like 14, isoform 1). (Q9BZR8); Myeloid cell leukemia sequence 1, isoform 1 (Myeloid celldifferentiation protein) (Myeloid cell leukemia protein 1) (BCL2-related). (Q9UNJ1); Bcl-2 homologous antagonist/killer (Apoptosis regulator BAK). (O08734); Bcl-2 homologous antagonist/killer (Apoptosis regulator BAK) (BCL2-like 7 protein). (Q16611); Putative Bcl-2 homologous antagonist/killer 2 (Apoptosis regulatorBAK-2). (Q13014); Myeloid cell leukemia sequence 1. (Q6AYY5); Anti-apoptotic NR13. (Q90ZN1); Hypothetical protein. (Q6AYK4); Mcl-1. (Q7YRZ9); Bcl-2 related anti-apoptotic protein. (Q804D0); Similar to Apoptosis inhibitor, Bcl2 family proteins (Apoptosisinhibitor-like protein). (Q9DH00); RSORF1 N13-like protein. (Q9E1F2); Mus musculus adult male testis cDNA, RIKEN full-length enrichedlibrary, clone:4933430L01 product:weakly similar to APOPTOSISREGULATOR BCL-G LONG FORM. (Q9D3W3); Bcl-2 homolog. (Q9WRT6); BHP1 protein. (Q9N754); ORF 16; functional anti-apoptotic factor vBCL-2 homolog; EBV BHRF1homolog (Bcl-2) (Hypothetical protein) (ORF 16). (P90504); Bcl-2 homolog. (Q77NK4); GH01265p (BOK). (Q95U83); Bcl-2-like protein 13 (Mil1 protein) (Bcl-rambo). (P59017); Bcl-2-like protein 13 (Mil1 protein) (Bcl-rambo). (Q9BXK5); Apoptosis regulator BAX, membrane isoform alpha. (Q07812); Apoptosis regulator BAX, membrane isoform alpha. (O02703); Bax (Bcl2-associated X protein). (Q9I9N4); Bax. (Q98U13); Bax protein splice variant k. (Q9JKL3); Bax-protein. (Q8SQ43); Bax-sigma. (Q9NYG7); Bax isoform psi. (Q8WZ49); Hypothetical protein MGC76049. (Q6P4Y4); Bax. (Q8HYU5); Bcl2-associated X protein kappa isoform. (Q8K3J2); Apoptosis regulator BAX, cytoplasmic isoform beta. (Q07814); Apoptosis regulator BAX, membrane isoform alpha. (Q63690); Apoptosis regulator BAX, membrane isoform alpha. (Q07813); Bcl2 like protein (Viral Bcl-2). (O40636); Orf 16; bcl-2 family member. (Q9YTQ0); Bcl-2-like gene 16 protein. (Q01001); BHP1 protein (BHP1g protein). (Q9N9X1); ORF64. (Q8BEN2); Bcl-2-like protein FPV039. (Q9J5G4); Apoptosis regulator Bcl-2 homolog precursor (LMH-5W). (Q07819); Apoptosis regulator Bcl-2 homolog precursor (LMH-5W). (Q07818); Apoptosis regulator Bcl-2 homolog precursor. (P42485); Putative apoptotic regulator. (Q70HB1); Bcl-x homologous

Bro-N~Bro-N	Culex nigripalpus baculovirus; Helicoverpa armigera nucleopolyhedrovirus G4; X. fastidiosa;	CUN001 putative bro protein, ATP_GTP_A motif, similar to AcMNPV ORF2. (Q919R4); CUN108 putative bro protein, ATP_GTP_A motif, similar to AcMNPVORF2. (Q919G9); Bro. (Q99GY7); CUN109 putative bro protein, ATP_GTP_A motif, similar to AcMNPVORF2. (Q919G8); Phage-related protein. (Q9PCU2);
Bro-N~Bro-N~Bro-N	X. fastidiosa (strain Temecula1 / ATCC 700964); X. fastidiosa;	Phage-related protein. (Q9PAJ2); Phage-related protein. (Q87AU0); Phage-related protein. (Q87CE5); Phage-related protein. (Q9PFH4);
CBM_14	Adoxophyes honmai nucleopolyhedrovirus; Adoxophyes orana granulovirus (AoGV); Agrotis segetum granulosis virus (AsGV) (Agrotis segetum granulovirus); Anopheles gambiae (African malaria mosquito); Anopheles gambiae str. PEST; Autographa californica nuclear polyhedrosis virus (AcMNPV); Blomia tropicalis (Mite); Bombyx mori nuclear polyhedrosis virus (BmNPV); Branchiostoma belcheri tsingtaunese; Caenorhabditis elegans; Choristoneura fumiferana defective nucleopolyhedrovirus; Choristoneura fumiferana granulovirus (CfGV); Choristoneura fumiferana nuclear polyhedrosis virus (CfMNPV); Cladosporium fulvum (Fulvia fulva); Cryptophlebia leucotreta granulosis virus (ClGV) (Cryptophlebia leucotreta granulovirus); Ctenocephalides felis (Cat flea); Culex nigripalpus baculovirus; Cydia pomonella granulosis virus (CpGV) (Cydia pomonella granulovirus); Drosophila melanogaster (Fruit fly); Entamoeba dispar; Entamoeba histolytica; Epiphyas postvittana nucleopolyhedrovirus (EppoMNPV); Helicoverpa armigera nuclear polyhedrosis virus; Helicoverpa armigera nucleopolyhedrovirus G4; Helicoverpa armigera single nucleocapsid polyhedrovirus; Helicoverpa zea single nucleocapsid nucleopolyhedrovirus; Heliothis zea virus 1; Lymantria dispar multicapsid nuclear polyhedrosis virus (LdMNPV); Mamestra configurata nucleopolyhedrovirus (MacoNPV); Mamestra configurata nucleopolyhedrovirus A; Mamestra configurata nucleopolyhedrovirus B; Neodiprion lecontei nucleopolyhedrovirus; Neodiprion sertifer nucleopolyhedrovirus; Orgyia pseudotsugata multicapsid polyhedrosis virus (OpMNPV); Ornithodoros moubata (Soft tick); Paramecium bursaria chlorella virus 1 (PBCV-1); Phthorimaea operculella granulovirus; Plutella xylostella granulovirus; Rachiplusia ou multiple nucleopolyhedrovirus (RoMNPV); R. solanacearum; Riftia pachyptila (Tube worm); Spodoptera exigua nucleopolyhedrovirus; Spodoptera litura multicapsid nucleopolyhedrovirus (SpltMNPV); Tachypleus tridentatus (Japanese horseshoe crab); Xestia c-nigrum granulosis virus (XnGV) (Xestia c-nigrum granulovirus);	CG13075-PA. (Q9VUX8*); CG12009-PA. (Q9VZR6*); CG7714-PA (RH24988p). (Q9VE40*); PROBABLE LIPOPROTEIN TRANSMEMBRANE. (Q8XZL0*); Race-specific elicitor A4 precursor. (Q00363*); Hypothetical protein W02A2.3. (Q9XUB6*); CG14957-PA. (Q9VZT6*); CG7549-PA. (Q9VHU5); CG13643-PA. (Q9VC14); CG14244-PA. (Q9VBC3); GH13023p (CG7549-PB). (Q95U23); Putative chitin binding protein. (Q8T5J2); AgCP12109. (Q7PX44); ORF79 similar to AcMNPV ORF150. (Q91EX6*); LP03531p (CG32284-PA). (Q8MS92*); Putative chitin binding protein. (Q8MU77); CG32036-PB. (Q9VT02); ORF_66. (Q7T9U9*); CG15313-PA. (Q9W2Z1); RE19838p (CG5756-PB). (Q8SXJ4*); ENSANGP00000023258. (Q7PF25*); CG5756-PA. (Q9V8B7*); CG31893-PA (LP19665p). (Q9VLL9*); P91 capsid protein. (Q8JKR5*); CG7874-PA (GH13361p). (Q9VWK3); Variable region-containing chitin-binding protein 4. (Q6IWM9); CG14301-PA. (Q9VE56); CG32656-PA. (Q8IR84); RE32881p. (Q7JXX2); CUN035 putative p91 capsid virion protein, similar to AcMNPVORF83. (Q919N4); Mucin/peritrophin-like protein precursor. (Q6QZV3); Hypothetical protein. (Q8B9B3); P95. (Q8B9H1); CG14959-PA. (Q9VZR9*); P95=AcMNPV orf83. (O92446*); Capsid associated protein. (Q7TLR9*); AcMNPV orf145. (O92497); 91K-like protein. (Q8JM52*); Capsid associated protein VP91. (Q80LN0); P95 protein. (O57075*); ORF78. (Q8V5S3*); CG13675-PA. (Q9VSE5); Vp91 capsid. (Q8QLD3*); Hypothetical 11.2 kDa protein in IE1-IEN intergenic region. (P41707*); Vp91 capsid. (Q77K65); Hypothetical 91.1 kDa protein (ORF86). (O10336*); Hypothetical protein. (Q91GH4*); Hypothetical protein. (Q91GH8*); Hypothetical 96.2 kDa protein in GP41-PNK intergenic region precursor. (Q06670*); ORF101 VP91 capsid. (Q91EV4); AcMNPV orf150. (O92502*); Viral capsid protein 91. (Q6JK76); CG14959-PC (RE24633p). (Q7KV71); Viral capsid associated protein. (Q6JP85); Vp91capsid. (Q99GX2*); ORF77 virion protein 91kD (Vp91). (Q9J860*); CG8192-PA. (Q9V7D4*); LD06965p. (Q8T0F0*); Viral capsid associated protein. (Q6VTR1); 91K. (Q71AB1); CG14607-PA. (Q9VI81*); CG14880-PA (Cg14880-pb) (RE15157p). (Q9VEW1*); RE01745p. (Q8SX53*); Hypothetical protein. (Q7T5S8*); ORF9 similar to AcMNPV ORF145. (Q91F35); Hypothetical protein. (Q8JMB4*); AgCP9296. (Q7PYX0); CG13676-PA. (Q9VSE3*); Hypothetical protein. (Q91BJ6*); Peritrophin-like protein 3. (Q8N0M7*); ORF86. (Q77IW4); Hypothetical protein PhopGV043. (Q8JS16*); Orf86-like protein. (Q8V5R8*); Exoskeleton beta-chitin-specific binding protein. (Q9NB50*); SeORF68-like protein. (Q77LZ7); ORF68. (Q9J867*); Hypothetical protein. (O10349*); Hypothetical protein. (Q6VTN5); LdOrf-30 peptide. (Q9YMU4*); Hypothetical protein. (Q91BW0*); ORF20. (Q9PZ23*); Hypothetical protein. (Q91BK1); Hypothetical protein. (Q99GW5*); AgCP5629. (Q7Q5Q4*); Hypothetical protein. (Q8JLZ1); Hypothetical protein. (Q8QL70*); AcNPV ORF145 homolog (ORF11). (P89258); ORF12. (Q99H35); ORF134. (Q9J809); Hypothetical protein. (Q77KA1); Hypothetical protein. (Q80LS5); RH43162p. (Q8MQI4*); Hypothetical protein. (Q77LZ2); Hypothetical protein. (Q71A48); ORF10. (Q6QXC9); ORF105. (Q9PYT8*); Hypothetical protein. (Q91GC8); Hypothetical protein. (Q8QLJ1*); Hypothetical protein PhopGV008. (Q8JS51); Hypothetical protein. (Q71AG9); ORF9. (Q8JL58*); Tachycitin. (P91818*); Hypothetical protein. (Q6VTK1); RE30702p. (Q8SYW5*); CG31439-PA. (Q8IMS9); Major allergen Blo t 12 precursor. (Q17282*); CG14304-PA. (Q9VE59); Hypothetical 11.0 kDa protein (ORF142). (O10373); ORF67. (Q6QXQ0); PxORF59 peptide. (Q9DVX4*); CG14608-PA. (Q9VI80); Cyst wall-specific glycoprotein Jacob. (Q962P5*); Cyst wall-specific glycoprotein Jacob. (Q962P6*); A332L protein. (Q84646); RH52423p. (Q8SWZ5*); CG14959-PB. (Q8IRD9);
CBM_4_9~CBM_4_9	P. aeruginosa;	Hypothetical protein. (Q9I060*);

CBS~CBS~GHMP_kinases	B. japonicum;	Blr5970 protein. (Q89HM0);
CBS~HisKA~HATPase_c	P. aeruginosa;	Probable two-component sensor. (Q9HZW5);
CD225	Bos taurus (Bovine); Homo sapiens (Human); Ictalurus punctatus (Channel catfish); Mus musculus (Mouse); Oncorhynchus mykiss (Rainbow trout) (Salmo gairdneri); Rattus norvegicus (Rat); Strongylocentrotus purpuratus (Purple sea urchin); Torpedo marmorata (Marbled electric ray); X. axonopodis (pv. citri); X. campestris (pv. campestris); Xenopus laevis (African clawed frog); Xenopus tropicalis (Western clawed frog) (Silurana tropicalis);	Mus musculus adult male corpora quadrigemina cDNA, RIKEN full-lengthenriched library, clone:B230312J21 product:unknown EST, full insertsequence. (Q8BR26); Interferon Inducible Protein 2. (Q8QFL3); Interferon inducible protein 1. (Q8QFM4); Putative haemopoietic membrane protein (Fragilis4). (O88728); Similar to RIKEN cDNA 6330512M04 gene. (Q810P6); Transmembrane protein. (Q8PD53); MGC64538 protein. (Q7SYS1); Interferon-induced transmembrane protein 1 (Interferon-induced protein17) (Interferon-inducible protein 9-27) (Leu-13 antigen) (CD225antigen). (P13164); Interferon-induced protein 1-8U. (Q95MQ3); Inteferon-induced membrane protein Leu-13/9-27. (Q95MQ2); Hypothetical protein. (Q9NC83); Interferon-inducible mRNA (cDNA 1-8). (Q14617); Ifitm2 protein (Interferon induced transmembrane protein 2) (FragilisR). (Q99J93); Interferon-inducible protein 16 (Hypothetical protein). (Q9R175); Mus musculus 10 day old male pancreas cDNA, RIKEN full-length enrichedlibrary, clone:1810061A10 product:INTERFERON-INDUCIBLE PROTEIN homolog(Mus musculus 18-day embryo whole body cDNA, RIKEN full-lengthenriched library, clone:1110004C05 product:INTERFERON-INDUCIBLEPROTEIN homolog) (Fragilis) (Interferon-inducible protein 15)(Interferon induced transmembrane protein 3). (Q9CQW9); Mus musculus 10 day old male pancreas cDNA, RIKEN full-length enrichedlibrary, clone:1810060G19 product:INTERFERON-INDUCIBLE PROTEINhomolog. (Q9D8L6); Interferon-inducible protein. (P26376); Interferon-induced transmembrane protein 3 (Interferon-inducibleprotein 1-8U). (Q01628); Interferon-induced transmembrane protein 2 (Interferon-inducibleprotein 1-8D). (Q01629); Mus musculus adult male testis cDNA, RIKEN full-length enrichedlibrary, clone:4930507H06 product:similar to INTERFERON-INDUCIBLEPROTEIN. (Q8BVR2); Mus musculus 18-day embryo whole body cDNA, RIKEN full-length enrichedlibrary, clone:1110036C17 product:similar to INTERFERON-INDUCIBLEPROTEIN (Fragilis2). (Q9D103); LOST1. (Q8IXB3); Mus musculus adult male testis cDNA, RIKEN full-length enrichedlibrary, clone:4933438K12 product:similar to INTERFERON-INDUCIBLEPROTEIN. (Q9D3R8); Hypothetical protein. (Q6DFT4); Interferon induced transmembrane protein 1. (Q8R2S7); Mus musculus 16 days embryo head cDNA, RIKEN full-length enrichedlibrary, clone:C130069F04 product:hypothetical protein, full insertsequence. (Q8C838); 14 kDa transmembrane protein. (Q91499); Transmembrane protein. (Q8PQ26*); Interferon induced protein 2. (Q8JH61*); Hypothetical protein FLJ90091. (Q8N2N8); Hypothetical protein DKFZp547J199. (Q8ND36); Similar to lymphocyte antigen 6 complex, locus G5B; G5b protein; openreading frame 31. (Q7Z6L0); Similar to lymphocyte antigen 6 complex, locus G5B; G5b protein; openreading frame 31. (Q96FA8); Interferon-inducible protein variant 10. (Q9R176);
CHASE2~PAS~HisKA~HATPase_c	P. aeruginosa;	Probable two-component sensor. (Q9HWZ1*);
CHASE3~GAF~GGDEF	X. axonopodis (pv. citri); X. campestris (pv. campestris);	Sensor histidine kinase. (Q8PB32*); Sensor histidine kinase. (Q8PMT1*); Sensor histidine kinase. (Q8PME1*);
CHASE3~GGDEF	X. campestris (pv. campestris);	Sensor histidine kinase. (Q8PAN4*);
CHASE3~HAMP~HisKA~HATPase_c~Response_reg	X. axonopodis (pv. citri); X. campestris (pv. campestris);	Two-component system sensor protein. (Q8PCA9*); Two-component system sensor protein. (Q8PNZ1*);
CHASE3~HAMP~PAS~PAC~HisKA~HATPase_c~Response_reg	P. syringae (pv. tomato);	Sensory box histidine kinase/response regulator. (Q884G4);
CHASE3~HisKA_3~HATPase_c	R. solanacearum;	TRANSMEMBRANE SENSOR KINASE VSRA TRANSCRIPTION REGULATOR PROTEIN (EC2.7.-.-). (Q8Y2P6*); VsrA. (Q52558);
CHASE3~HisKA~HATPase_c~Response_reg	B. japonicum;	Two-component hybrid sensor and regulator. (Q89MB7);
CHASE3~PAS~HisKA~HATPase_c	B. japonicum;	Two-component hybrid sensor and regulator. (Q89IE7);
CHASE4~HAMP~PAS~PAC~GGDEF	P. aeruginosa;	Hypothetical protein. (Q9I594);
CHASE~HWE_HK	M . loti;	Sensory transduction histidine kinase. (Q98FM2*);

CHASE~PAS	P. syringae (pv. tomato);	CHASE domain/PAS domain protein. (Q883N8);
CHASE~PAS~PAC~PAS~PAC~HisKA~HATPase_c~Response_reg~Response_reg~Hpt	X. axonopodis (pv. citri); X. campestris (pv. campestris);	Two-component system sensor protein. (Q8P883*); Two-component system sensor protein. (Q8PJN8*);
CHASE~PAS~PAC~PAS~PAC~PAC~HisKA~HATPase_c~Response_reg~Response_reg~Hpt	P. aeruginosa;	Probable sensor/response regulator hybrid. (Q9HWR8);
CMD~CMD~CMD	S. meliloti;	Hypothetical protein. (Q930M4);
COesterase~Abhydrolase_2	X. axonopodis (pv. citri);	Carboxylesterase. (Q8PJY4);
Cache~HAMP~GAF~HisKA~HATPase_c	B. japonicum;	Two-component hybrid sensor and regulator. (Q89RB0);
Cache~HAMP~Guanylate_cyc	B. japonicum; Rhizobium sp. (strain NGR234); Spirulina platensis;	Blr6052 protein. (Q89HD9); Adenylate cyclase (EC 4.6.1.1). (Q9EXQ2*); Hypothetical protein. (Q6W199);
Cache~HAMP~HAMP~MCPsignal	S. meliloti;	PROBABLE CHEMORECEPTOR (METHYL-ACCEPTING CHEMOTAXIS) TRANSMEMBRANEPROTEIN. (Q92SH9); Methyl-accepting chemotaxis protein McpX. (Q9F5M7);
Cache~HAMP~PAS~PAC~PAS~PAS~PAC~GGDEF	R. solanacearum;	PROBABLE TRANSMEMBRANE PROTEIN. (Q8XTU2);
Cache~HAMP~SpoIIE	Desulfotalea psychrophila LSv54; M . loti; S. meliloti; Rhizobium sp. (strain NGR234); X. axonopodis (pv. citri);	Related to ICFG protein. (Q6AND8); Putative regulatory protein. (Q92WB1*); Mll6700 protein. (Q988K4*); Sigma factor sigB regulation protein rsbU. (Q6W2C9); IcfG protein. (Q8PPW1);
Calx-beta~Calx-beta~He_PIG~He_PIG~He_PIG~He_PIG~He_PIG~He_PIG~He_PIG~He_PIG~He_PIG~Autotransporter	X. campestris (pv. campestris);	Hemagglutinin. (Q8P377*);
Calx-beta~Exo_endo_phos	X. axonopodis (pv. citri); X. campestris (pv. campestris);	Nuclease. (Q8PDA9); Nuclease. (Q8PQ81);
Cellulase~CBM_2	Acidothermus cellulolyticus; Actinomyces sp. 40; Butyrivibrio fibrisolvens; Cellulomonas flavigena; Clostridium cellulovorans; Clostridium longisporum; Globodera rostochiensis (Golden nematode); X. axonopodis (pv. citri); X. campestris (pv. campestris); X. fastidiosa (strain Temecula1 / ATCC 700964); X. fastidiosa;	Endo-1,4-beta-glucanase (EC 3.2.1.4). (Q9S3V3); Endoglucanase E1 precursor (EC 3.2.1.4) (Endo-1,4-beta-glucanase E1)(Cellulase E1) (Endocellulase E1). (P54583*); Endoglucanase 1 (EC 3.2.1.4) (Endo-1,4-beta-glucanase) (Cellulase). (P20847*); Endo-1,4-beta-glucanase. (Q87AH4); Endo-1,4-beta-glucanase. (Q9PF60); Major extracellular endoglucanase precursor (EC 3.2.1.4) (Endo-1,4-beta-glucanase) (Cellulase). (P19487*); Endoglucanase D precursor (EC 3.2.1.4) (Endo-1,4-beta-glucanase C) (Cellulase D). (P28623*); Cellulase. (Q8PPS3*); Endoglucanase A precursor (EC 3.2.1.4) (Endo-1,4-beta-glucanase A)(Cellulase A). (P54937); Endoglucanase. (O66064); Beta-1,4-endoglucanase (EC 3.2.1.4). (O16028*);
Cellulase~DPBB_1	X. campestris (pv. campestris); X. fastidiosa (strain Temecula1 / ATCC 700964); X. fastidiosa;	Cellulase. (Q8P513*); Extracellular endoglucanase. (Q87AG9); Extracellular endoglucanase. (Q9PF68);
CheB_methylest~CheR_N~CheR~PAC~PAS~PAC~HWE_HK	S. meliloti;	Putative chemotaxis methyltransferase protein. (Q92W49);
CheR_N~CheR~HWE_HK	X. axonopodis (pv. citri);	Methyltransferase. (Q8PG87);
CheR~TPR_1	R. solanacearum;	PUTATIVE METHYLTRANSFERASE PROTEIN. (Q8XPJ3);
CheR~TPR_2	Myxococcus xanthus; S. meliloti;	Putative methyltransferase-chemotaxis. (Q92YM8); CheR4. (Q8KQ64);
Chitin_synth_1~DegT_DnrJ_EryC1	E. carotovora subsp. atroseptica SCRI1043;	Putative bifunctional enzyme including aminotransferase and chitinsynthase. (Q6D5J4);
Chorismate_bind~Chorismate_bind~GATase	P. aeruginosa PAO1;	Phenazine biosynthesis protein PhzE. (Q7DC81);

Colicin	Citrobacter freundii; Escherichia coli; Escherichia fergusonii; Hafnia alvei; Plasmid ColE1; Plasmid ColIb-P9; P. aeruginosa; Serratia marcescens; Shigella boydii; Shigella sonnei;	Colicin Y. (Q9KJ98); Colicin U. (O24681); Colicin S4. (Q9XB47); Colicin A. (P04480); Colicin N. (P08083); Bacteriocin 28b. (Q06308); Colicin E1. (Q8GH13); Colicin E1. (Q8GH14); Pyocin S5. (Q9I4Y4); Colicin protein. (Q46736); Colicin protein. (Q46740); Colicin protein. (Q46734); Colicin protein. (Q46737); Colicin E1 protein. (Q51626); Colicin protein. (Q46745); Colicin protein. (Q46744); Colicin protein. (Q46732); Colicin protein. (Q46738); Colicin protein. (Q57462); Colicin protein. (Q46742); Colicin protein. (Q46743); Colicin Ib. (Q8G8L5); Colicin protein. (Q46746); Colicin Ib protein. (Q7DJZ6); Alveicin A bacteriocin toxin. (Q6WRX0); Alveicin B bacteriocin toxin. (Q6WRW3); Colicin Ib protein. (P04479); Colicin Ia protein. (P06716); Colicin Ia. (Q8GH10); Colicin E1. (Q8GH12); Colicin K. (Q47502); Colicin E1* protein. (P21178); Colicin E1 protein. (P02978); Colicin 10. (Q47125); Colicin 5. (Q47500); Pyocin S5. (Q7WXX2);
Collar~Collar~Collar	B. japonicum;	Blr4714 protein. (Q89L34);
Condensation~AMP-binding~PP-binding~Condensation~AMP-binding~PP-binding~Condensation~AMP-binding~PP-binding~Condensation~AMP-binding~PP-binding~Condensation~AMP-binding~PP-binding~Condensation~AMP-binding~PP-binding~Condensation~AMP-binding~PP-binding	E. carotovora subsp. atroseptica SCRI1043;	Non-ribosomal peptide synthetase. (Q6D738);
Condensation~AMP-binding~PP-binding~Condensation~AMP-binding~PP-binding~Condensation~AMP-binding~PP-binding~Condensation~AMP-binding~PP-binding~Condensation~AMP-binding~PP-binding~Condensation~AMP-binding~PP-binding~Thioesterase~Thioesterase	E. carotovora subsp. atroseptica SCRI1043;	Non-ribosomal peptide synthetase. (Q6D739);
Condensation~AMP-binding~PP-binding~Condensation~AMP-binding~PP-binding~Condensation~AMP-binding~PP-binding~Condensation~AMP-binding~PP-binding~Thioesterase	Bacillus brevis (Brevibacillus brevis); P. syringae (pv. tomato);	Non-ribosomal peptide synthetase, terminal component. (Q87W61); Gramicidin S synthetase II [Includes: ATP-dependent proline adenylase(ProA) (Proline activase); ATP-dependent valine adenylase (ValA)(Valine activase); ATP-dependent ornithine adenylase (OrnA) (Ornithineactivase); ATP-dependent leucine adenylase (LeuA) (Leucine activase)]. (P14688); Gramicidin S synthetase 2. (Q44928);
Condensation~AMP-binding~PP-binding~Condensation~Condensation~AMP-binding~PP-binding~Condensation~AMP-binding~PP-binding~Condensation~Condensation~AMP-binding~PP-binding	P. aeruginosa;	Probable non-ribosomal peptide synthetase. (Q9I179);
Condensation~AMP-binding~PP-binding~Condensation~Condensation~Condensation~PP-binding	E. carotovora subsp. atroseptica SCRI1043;	Putative non-ribosomal peptide synthetase. (Q6D9Y1);
Condensation~Condensation~AMP-binding~PP-binding~Condensation~AMP-binding~PP-binding~Condensation~AMP-binding~PP-binding~Condensation~AMP-binding~PP-binding~Condensation~AMP-binding~PP-binding~Thioesterase~Thioesterase	P. syringae (pv. tomato); R. solanacearum;	PROBABLE PEPTIDE SYNTHETASE PROTEIN. (Q8XS39); Non-ribosomal peptide synthetase, terminal component. (Q881Q3);
Cu-oxidase_3~Cu-oxidase_2~Cu-oxidase	B. japonicum;	Respiratory nitrite reductase. (Q89EJ6); Respiratory nitrite reductase. (O31380*);
Cu-oxidase_3~Cu-oxidase_3~Cu-oxidase_2	B. japonicum; Streptomyces antibioticus; Streptomyces lavendulae;	Laccase precursor (EC 1.10.3.2). (Q8GB87); Phenoxazinone synthase (EC 1.-.-.-) (PHS). (Q53692); Bll2293 protein. (Q89SV7);
Cupin_3~HTH_AraC~HTH_AraC	Pseudomonas fluorescens; X. campestris (pv. campestris);	Transcriptional regulator. (Q8PDM4); Regulatory protein. (Q83V19);

Cytochrom_C~Cytochrom_C~Cytochrom_C	P. aeruginosa; S. meliloti; Vibrio marinus (Moritella marina);	Probable cytochrome c. (Q9HVL3*); Putative cytochrome c class I protein, probably cytochrome c4. (Q92UY9*); ORF21. (Q9RA08*);
Cytochrom_C~Cytochrom_D1	Alcaligenes eutrophus (Ralstonia eutropha); Burkholderia cepacia (Pseudomonas cepacia); Paracoccus denitrificans; Paracoccus pantotrophus (Thiosphaera pantotropha); P. aeruginosa; Pseudomonas fluorescens; Pseudomonas stutzeri (Pseudomonas perfectomarina);	Nitrite reductase precursor (EC 1.7.2.1) (Cytochrome cd1) (Cytochromeoxidase) (Hydroxylamine reductase) (EC 1.7.99.1). (P24040*); Nitrite reductase precursor. (Q52519); Nitrite reductase precursor (EC 1.7.2.1) (Cytochrome cd1) (Cytochromeoxidase) (Hydroxylamine reductase) (EC 1.7.99.1). (Q51700*); Nitrite reductase precursor (EC 1.7.2.1) (Cytochrome cd1) (Cytochromeoxidase) (Hydroxylamine reductase) (EC 1.7.99.1). (P72181*); Nitrite reductase precursor (EC 1.7.2.1) (Cytochrome cd1) (Cytochromeoxidase) (Hydroxylamine reductase) (EC 1.7.99.1). (P24474*); Cd1 nitrite reductase. (Q9F0W9*); Cytochrome cd1 nitrite reductase precursor. (Q44012*); NirS protein. (Q76KD7); NirN precursor. (P95418*); ORF507 protein. (P95552*); NirN protein. (Q76KD8); Probable c-type cytochrome. (Q9I609*);
Cytochrom_D1	Burkholderia cepacia (Pseudomonas cepacia); Paracoccus denitrificans; P. aeruginosa; Pseudomonas stutzeri (Pseudomonas perfectomarina); Pyrobaculum aerophilum;	NirF protein. (Q76KE5); NirF protein. (Q51480*); NirF protein. (Q52521*); Hypothetical protein nirF precursor. (Q9R9J9*); Nitrite reductase (Cytochrome d1), conjectural. (Q8ZST1);
DUF1036~PG_binding_1~DUF1036	B. japonicum;	Blr8102 protein. (Q89BP6);
DUF1217~DUF1217	A. tumefaciens ;	Hypothetical protein Atu0653. (Q8UHM7);
DUF1217~DUF1217~DUF1217	A. tumefaciens ; B. japonicum; S. meliloti;	Hypothetical protein SMc00986. (Q92KK3); AGR_C_1161p. (Q7D105); Hypothetical protein Atu2524. (Q8UCG3); AGR_C_4586p. (Q7CWV0); Blr4709 protein. (Q89L39);
DUF1236~DUF1236	B. japonicum;	Bll8057 protein. (Q89BU1); Hypothetical exported glutamine-rich protein. (Q89D03);
DUF1254	Archaeoglobus fulgidus; M . loti; S. meliloti;	Mll5469 protein. (Q98BQ5*); Hypothetical protein. (Q92ZY0); Hypothetical protein AF2162. (O28120*);
DUF126~DUF521	A. tumefaciens ; P. aeruginosa; S. meliloti;	Hypothetical protein Atu4683. (Q8U6X3); AGR_L_398p. (Q7CVK7); Hypothetical protein SMb20269. (Q92WS0); Hypothetical protein. (Q9I485);
DUF1289~NUDIX	X. campestris (pv. campestris);	MutT/nudix family protein. (Q8PAE9);
DUF1332~DnaJ	A. tumefaciens ; S. meliloti;	Hypothetical protein SMc01853. (Q92K37); Molecular chaperone, DnaJ family. (Q8UDL1); AGR_C_3834p. (Q7CXV1);
DUF1403	Agrobacterium rhizogenes; A. tumefaciens; Oligotropha carboxidovorans (Pseudomonas carboxydovorans); M . loti; Ruegeria sp. PR1b;	Mlr9360 protein. (Q981I5); Mlr6196 protein. (Q98A16); Mll9647 protein. (Q98P18); Riorf137 protein. (Q9F5B5); Ylf. (O52286); HYPOTHETICAL CONSERVED PROTEIN. (Q8KGW3); RC231. (Q8KVV9); Hypothetical protein. (Q6LBD5); Mll9348 protein. (Q981J5*); Msl9347 protein. (Q981J6);
DUF1419	Agrobacterium rhizogenes; A. tumefaciens ; A. tumefaciens; Oligotropha carboxidovorans (Pseudomonas carboxydovorans); M . loti;	Mll9335 protein. (Q981K6); Hypothetical protein riorf91 (Hypothetical protein orf91). (Q9KW93); Hypothetical protein Atu6099. (Q8U636); Hypothetical protein. (Q6LB72); Tiorf45 protein. (Q9R6L4); Hypothetical protein Atu5101. (Q8UKK1); AGR_pAT_147p. (Q7D3X0); AGR_pTi_186p. (Q7D2I5);
DUF1427	A. tumefaciens ; B. japonicum; P. aeruginosa; R. solanacearum; Rhizobium leguminosarum (biovar trifolii); S. meliloti; X. campestris (pv. campestris);	Hypothetical protein XCC2052. (Q8P914); Bsl6958 protein. (Q89EW2); Hypothetical protein. (Q93EB2); HYPOTHETICAL TRANSMEMBRANE PROTEIN. (Q8Y2U1*); AGR_L_1747p. (Q8U4X9*); Hypothetical protein. (Q92Y85); Bsr4258 protein. (Q89MD5); Hypothetical protein. (Q9I0E5*);
DUF1428~DUF1428	M . loti;	Mll7839 protein. (Q984U4);
DUF1484	R. solanacearum;	Hypothetical protein RSc3215. (Q8XUH3); Hypothetical protein RSc3085. (Q8XUU8); Hypothetical protein RSc0885. (Q8Y108); Hypothetical protein RSc3190. (Q8XUJ8); Hypothetical protein RSc0966. (Q8Y0S7);
DUF1486	A. tumefaciens ; Neurospora crassa; P. aeruginosa; P. syringae (pv. tomato); R. solanacearum; M . loti; S. meliloti;	Hypothetical protein. (Q7SFH5); Hypothetical protein Atu3018. (Q8UBJ8); Hypothetical protein. (Q92YL1); Mlr2224 protein. (Q98IW1); Hypothetical protein. (Q9I3U3); Hypothetical protein. (Q9JP27); AGR_L_3571p. (Q7CRD4); Hypothetical protein RSc0819. (Q8Y171);
DUF1501~DUF1501	E. carotovora subsp. atroseptica SCRI1043;	Putative exported protein. (Q6D5J3);

DUF1515	M . loti; S. meliloti;	Mlr8550 protein. (Q982P6); Mlr8037 protein. (Q984E6); Mlr9754 protein. (Q98NT1); Hypothetical protein SMb20519. (Q92W45); HYPOTHETICAL TRANSMEMBRANE PROTEIN. (Q92NZ0);
DUF1520	B. japonicum; S. meliloti;	Bll4817 protein. (Q89KT6); Hypothetical protein SMa1082. (Q92ZA5); Hypothetical protein sm12C4.5. (Q9R9N6); Bll7993 protein. (Q89C05);
DUF1520~DUF1520	B. japonicum;	Bll6557 protein. (Q89FZ1); Bll2849 protein. (Q89RC3); Bll0361 protein. (Q89XF3);
DUF1521~DUF1521	B. japonicum;	Blr1649 protein. (Q89TX4); ID205. (Q9ANI0); Blr1806 protein. (Q79US6);
DUF1534	P. syringae (pv. tomato);	Hypothetical protein. (Q87Z14); Hypothetical protein. (Q87Z15); Hypothetical protein. (Q87XT9); Hypothetical protein. (Q87XU0); Hypothetical protein. (Q88B95); Hypothetical protein. (Q87V97);
DUF1534~DUF1534~DUF1534~DUF1534~DUF1534	P. syringae (pv. tomato);	Hypothetical protein. (Q87W99);
DUF1629	X. axonopodis (pv. citri); X. campestris (pv. campestris);	Hypothetical protein XCC1624. (Q8PA65); Hypothetical protein XCC1620. (Q8PA69); Hypothetical protein XAC2246. (Q8PKC9); Hypothetical protein XAC3319. (Q8PHD8); Hypothetical protein XAC3267. (Q8PHI7); Hypothetical protein XAC2862. (Q8PIN3); Hypothetical protein XAC3322. (Q8PHD7); Hypothetical protein XAC2507. (Q8PJM3); Hypothetical protein XAC2635. (Q8PJA4);

DUF239	Arabidopsis thaliana (Mouse-ear cress); Gossypium hirsutum (Upland cotton); Oryza sativa (japonica cultivar-group); X. axonopodis (pv. citri); Zea mays (Maize);	Carboxyl-terminal proteinase-like. (Q6ZAJ4); F14N23.7. (Q9SY61); F26F24.22. (Q9LR24*); Carboxyl-terminal peptidase-like. (Q6Z1P7); Similarity to carboxyl-terminal proteinase. (Q9FIP3); Similarity to carboxyl-terminal proteinase. (Q9FIP4); Expressed protein (At2g44210/F4I1.2) (Hypothetical protein). (O64856*); Hypothetical protein P0419C04.13. (Q75M42); Hypothetical protein At3g48230/T24C20_110 (At3g48230). (Q8GX31); Hypothetical protein T24C20_110. (Q9STR5); Putative ZmEBE-1 protein. (Q69Y39); Similarity to carboxyl-terminal proteinase (Hypothetical proteinAt5g56530). (Q9LVC1*); Putative carboxyl-terminal proteinase. (Q93X76); Gb|AAF04872.1 (AT5g50150/MPF21_17). (Q9FG96*); Hypothetical protein. (Q8LFH3*); P0583G08.7 protein. (Q942T2*); Hypothetical protein At2g44250. (O64860*); OSJNBa0026J14.25 protein. (Q8RYQ9); Hypothetical protein At1g55360. (Q8GUK9); Putative carboxyl-terminal peptidase. (Q84JS7); Hypothetical protein At1g23340/F26F24_11 (At1g23340). (Q8GY67); Hypothetical protein OSJNBa0059E14.15. (Q84M37); F20B24.18. (Q9SGX5); Putative carboxyl-terminal peptidase. (Q8LAN5*); At1g70550. (Q84VY0); Gb|AAC16072.1 (AT3g13510/MRP15_15). (Q9LJE0*); Hypothetical protein F7A10.1. (Q9C8A7*); Hypothetical protein F5A18.27 (Hypothetical protein F24J13.12). (Q9S7X2*); T1N24.4 protein. (Q9XGZ4*); Putative carboxyl-terminal proteinase. (Q6ZL19); Putative DD1A protein. (Q6ZIY2); T16B5.11 protein. (Q9SAC7); Hypothetical protein XAC3314. (Q8PHE3); Hypothetical protein At2g44220/F4I1.3. (Q8L8B1); Hypothetical protein At2g44220. (O64857*); ZmEBE-1 protein. (Q84JV5); P0506B12.3 protein (P0460C04.23 protein). (Q8S1T9*); Expressed protein (At2g44240/F4I1.5). (O64859*); Hypothetical protein At4g23390/F16G20_90 (At4g23390). (Q8GXV8); Similarity to carboxyl-terminal proteinase (Hypothetical proteinAt5g05030). (Q9FF71*); Hypothetical protein At2g27320. (Q9XIN9*); Similarity to carboxyl-terminal proteinase. (Q9FGP1*); Hypothetical protein At2g35250. (O82168*); Hypothetical protein T22P22_50. (Q9LYG4); P0518C01.31 protein (P0698H10.9 protein). (Q94DF7); Hypothetical protein At2g20170. (Q9SL64*); Hypothetical protein F16G20.90 (Hypothetical protein AT4g23390). (O81735); Emb|CAB87684.1. (Q9LHT4*); T1N24.5 protein. (Q9XGZ6*); Emb|CAB87684.1. (Q9FG52); AT5g25410/F18G18_150. (Q941C9*); Emb|CAB87684.1. (Q9FKK5); Hypothetical protein F16G20.80 (Hypothetical protein AT4g23380). (O81734*); P0503E05.29 protein (P0697D09.16 protein). (Q9ARU9*); Expressed protein. (Q75IE5); Hypothetical protein At1g10190. (Q8VYE1); Putative DD1A protein. (Q6ZLC1); Hypothetical protein At2g24950. (Q9SK38*); Gb|AAD23018.1. (Q9FGI5*); Putative DD1A protein. (Q6Z3Q6); Hypothetical protein OJ1123_D06.9. (Q75IE7); Hypothetical protein OJ1695_H09.14. (Q6K8Y8); Hypothetical protein AT4g17860. (O49685); Hypothetical protein F16G20.50 (Hypothetical protein AT4g23350). (O81731*); ZmEBE-2 protein. (Q84VQ8); ZmEBE-2 protein. (Q84VQ9); Hypothetical protein AT4g15050. (O23359*); Hypothetical protein F24G24.10 (Hypothetical protein AT4g10210). (Q9SV95*); OSJNBa0024F24.18 protein. (Q8LQ23*); Hypothetical protein F7H19.270 (Hypothetical protein AT4g23080). (O82757); OSJNBa0024F24.20 protein. (Q8LQ21*); Hypothetical protein P0700F06.4 (Hypothetical proteinOJ1249_F12.16). (Q6K693); OSJNBb0005B05.10 protein. (Q7XQ31*); T9A4.9 protein. (O82619*); Hypothetical protein F24G24.20 (Hypothetical protein AT4g10220). (Q9SV94); Hypothetical protein At2g44220/F4I1.3. (Q8L877); Hypothetical protein OJ1695_H09.15. (Q6K8Y7); T9A4.10 protein. (O82620*); T27C4.14 protein. (Q9M838);
DUF336~Glyoxalase	B. japonicum;	Blr6270 protein. (Q89GS5);
DUF35~adh_short	B. japonicum;	Blr3430 protein. (Q89PQ0);
DUF419~DUF419	E. carotovora subsp. atroseptica SCRI1043;	Hypothetical protein. (Q6D6T9);
DUF427~DUF427	M . loti; Rhodococcus erythropolis;	Hypothetical protein. (Q6XMX7); Mll7342 protein. (Q986I4); Mll2277 protein. (Q98IS0);
DUF442~Lactamase_B	A. tumefaciens ; M . loti; S. meliloti; X. fastidiosa (strain Temecula1 / ATCC 700964); X. fastidiosa;	Metallo-beta-lactamase superfamily protein. (Q8UAA9); Hypothetical protein. (Q92ZB8); AGR_L_2726p. (Q7CSJ2); Hypothetical protein. (Q87AD6); Mlr2158 protein. (Q98J12); Hypothetical protein. (Q9PFB0);

DUF680	M . loti;	Msr8665 protein. (Q98CK8*); Mll1966 protein. (Q98JF5); Msr7957 protein. (Q984L0*); Mlr7958 protein. (Q984K9); Msr7956 protein. (Q984L1); Mlr3103 protein. (Q98GZ6*); Msl1965 protein. (Q98JF6*); Msr5105 protein. (Q98CK9*); Msr7955 protein. (Q984L2); Msr5104 protein. (Q98CL0*);
DUF726	Arabidopsis thaliana (Mouse-ear cress); Ashbya gossypii (Yeast) (Eremothecium gossypii); Caenorhabditis elegans; Candida albicans (Yeast); Candida glabrata CBS138; Dictyostelium discoideum (Slime mold); Homo sapiens (Human); Kluyveromyces lactis NRRL Y-1140; Mus musculus (Mouse); Neurospora crassa; Oryza sativa (japonica cultivar-group); Plasmodium falciparum (isolate 3D7); Plasmodium yoelii yoelii; P. syringae (pv. tomato); Saccharomyces cerevisiae (Baker's yeast); Schizosaccharomyces pombe (Fission yeast); Yarrowia lipolytica CLIB99;	Hypothetical protein B1D1.130. (Q9P6A7); Hypothetical protein. (Q8SSU8); At2g18100. (Q84W08); Hypothetical protein At2g18100. (Q9SL37); SPAC607.08c protein. (Q9US10); Hypothetical protein. (Q7SGA3); Hypothetical protein. (Q7SEZ8); SPAC6F6.13c protein. (O14244); Hypothetical protein F35D11.3. (Q20035); Hypothetical protein Ca49C4.05c. (O94063); Similar to sp|P43564 Saccharomyces cerevisiae YFL034w singleton. (Q6CRZ9); Similar to sp|P43564 Saccharomyces cerevisiae YFL034w. (Q6FLY1); Similar to CAGL-IPF4629.1 Candida glabrata. (Q6C4H8); ACR220Cp. (Q75BQ1); Hypothetical 119.5 kDa protein in MOB2-RIM15 intergenic region. (P43564); Hypothetical protein. (Q7R7Y2); Hypothetical protein. (Q8I5Y6); Hypothetical protein LOC255104. (Q7Z6K6); Mus musculus 0 day neonate skin cDNA, RIKEN full-length enrichedlibrary, clone:4632413C14 product:DJ301K23.1 (NOVEL PROTEIN SIMILAR TOPREDICTED YEAST AND WORM PROTEINS) homolog (Mus musculus 10 daysneonate skin cDNA, RIKEN full-length enriched library,clone:4732490M16 product:DJ301K23.1 (NOVEL PROTEIN SIMILAR TOPREDICTED YEAST AND WORM PROTEINS) homolog). (Q9D675); RIKEN cDNA 4632413C14. (Q91WU4); Hypothetical protein DKFZp686C23231. (Q6MZN5); Hypothetical protein F23E13.100 (Hypothetical protein AT4g36210). (O65513); Hypothetical protein. (Q889K3); Mus musculus 2 days pregnant adult female ovary cDNA, RIKEN full-length enriched library, clone:E330017C12 product:DJ301K23.1 (NOVELPROTEIN SIMILAR TO PREDICTED YEAST AND WORM PROTEINS) homolog. (Q8BIX8); Hypothetical protein FLJ45636. (Q6ZSC6); Hypothetical protein OSJNBa0055O03.4. (Q9FW10);
DUF746~DUF746	R. solanacearum;	PUTATIVE TRANSPOSASE PROTEIN. (Q8XUK9); Hypothetical protein RSc0889. (Q8Y104);
DUF748~DUF748~DUF748~DUF748	R. solanacearum;	PUTATIVE TRANSMEMBRANE PROTEIN. (Q8Y0Z7);
DUF763	Aeropyrum pernix; Archaeoglobus fulgidus; B. japonicum; Methanobacterium thermoautotrophicum; Methanopyrus kandleri; Picrophilus torridus; Pyrobaculum aerophilum; Pyrococcus abyssi; Pyrococcus furiosus; Pyrococcus horikoshii; M . loti; S. meliloti; Sulfolobus solfataricus; Sulfolobus tokodaii; Thermoplasma acidophilum; Thermoplasma volcanium; X. axonopodis (pv. citri); X. campestris (pv. campestris);	Hypothetical protein XCC1094. (Q8PBM5); Hypothetical protein XAC1190. (Q8PN83); Hypothetical protein APE1824. (Q9YAX1); Hypothetical protein ST0586. (Q974S6); Hypothetical protein PF0611. (Q8U361); Hypothetical protein. (Q97VZ2); Hypothetical protein PH0745. (O58515); Hypothetical protein SMb21455. (Q92U57); Hypothetical protein. (Q9UZ46); Mlr6856 protein. (Q987Y3); Bll3834 protein. (Q89NK4); Uncharacterized conserved protein. (Q8TYA4); Hypothetical protein PAE0766. (Q8ZYH9); Hypothetical protein TVG0468151. (Q97BH6); Hypothetical protein Ta1095. (Q9HJ77); Hypothetical protein AF1496. (O28776); Hypothetical protein. (Q6L1J8); Hypothetical protein MTH448. (O26548); Hypothetical protein MTH449. (O26549);
DUF768	M . loti;	Msr8108 protein. (Q983Y6); Msr9525 protein. (Q98PB9); Msl4700 protein. (Q98DH6); Msl0435 protein. (Q98MU4); Msl0431 protein. (Q98MU8); Msr9731 protein. (Q98NV0); Mll9600 protein. (Q98P60); Msl9563 protein. (Q98P89); Msr9531 protein. (Q98PB6); Msr9526 protein. (Q98PB8); Msr5733 protein. (Q98B48); Msr9518 protein. (Q98PC5);
DUF769	X. fastidiosa (strain Temecula1 / ATCC 700964); X. fastidiosa;	Hypothetical protein. (Q87CS8); Hypothetical protein. (Q9PEY3*); Hypothetical protein. (Q9PEY7); Hypothetical protein. (Q9PEY2*); Hypothetical protein. (Q9PEY6*); Hypothetical protein. (Q87AN2); Hypothetical protein. (Q87AN4); Hypothetical protein. (Q9PEY4*); Hypothetical protein. (Q9PEY8*); Hypothetical protein. (Q87AN5); Hypothetical protein. (Q87CS6); Hypothetical protein. (Q9P9X4); Hypothetical protein. (Q879V6);
DUF802~DUF802	R. solanacearum; X. axonopodis (pv. citri);	Hypothetical protein XAC3753. (Q8PG64*); PROBABLE TRANSMEMBRANE PROTEIN. (Q8XQ05*);
DUF802~DUF802~DUF802	X. axonopodis (pv. citri);	Hypothetical protein XAC3754. (Q8PG63);
DUF802~DUF802~DUF802~DUF802~DUF802	X. campestris (pv. campestris);	Hypothetical protein XCC3711. (Q8P4J6*);

DUF811	P. aeruginosa; R. solanacearum;	Hypothetical protein. (Q9I6E4*); Hypothetical protein. (Q9I6E5*); Hypothetical protein RSc3082. (Q8XUV1);
DUF982	A. tumefaciens ; M . loti; S. meliloti; Rhizobium sp. (strain NGR234);	Hypothetical protein SMb21469. (Q92U47); AGR_C_3168p. (Q8U587); Mll0147 protein. (Q98NG7); Hypothetical protein. (Q6W279); Hypothetical protein SMb20550. (Q92W18); Hypothetical protein SMb20546. (Q92W22); Msr8381 protein. (Q983D0); Mll0951 protein. (Q98LN5); Hypothetical protein SMb21471. (Q92U45); Mll0594 protein. (Q98MG1); AGR_C_3319p. (Q8U579); Mll3139 protein. (Q98GX0); Hypothetical protein Atu1219. (Q8UG23); Hypothetical protein Atu5450. (Q8UJM6); Hypothetical protein Atu5053. (Q8UKP7); Hypothetical protein. (Q6W161); Hypothetical protein SMb21480. (Q92U36); Hypothetical protein SMb21470. (Q92U46); Hypothetical protein SMb21296. (Q92VA8); Hypothetical protein SMb20551. (Q92W17); Msr9401 protein. (Q981Y3); Mll6995 protein. (Q987M2); Mlr9590 protein. (Q98P69); AGR_pAT_661p. (Q7D320); AGR_C_2251p. (Q7CZQ4); AGR_C_724p. (Q8U5M2); Hypothetical protein SMc04238. (Q92P82); Msl6994 protein. (Q987M3);
DctM~DedA~DedA	A. tumefaciens ; B. japonicum;	ABC transporter, membrane spanning protein. (Q8UAW2*); AGR_L_3123p. (Q7CS03); Bll6832 protein. (Q89F69);
DctM~DedA~MCD	S. meliloti;	Hypothetical protein. (Q930W3);
Dehydratase_LU~Dehydratase_MU	M . loti;	Glycerol dehydratase large subunit. (Q988J0);
DspF	Erwinia amylovora; E. carotovora subsp. atroseptica SCRI1043; Erwinia pyrifoliae; Erwinia stewartii; Pantoea agglomerans (pv. gypsophilae) (Erwinia herbicola); Pectobacterium atrosepticum; P. syringae (pv. tomato); P. syringae;	DspF. (Q6RK54); DspF (DspB protein). (O54621); WtsF. (Q9FCY6); Putative avirulence protein. (Q6D5C6); DspF. (Q6QPL4); Hypothetical protein (Avirulence protein AvrF). (Q9JP37); Avirulence protein. (O66102); DspF. (Q9KH43);
EAL~PAC~PAC	R. solanacearum;	Hypothetical protein RSp1051. (Q8XR17);
Epimerase_2~UDPG_MGDP_dh_N~UDPG_MGDP_dh~ UDPG_MGDP_dh_C	B. japonicum;	Bll6307 protein. (Q89GN8);

F-box~LRR_1	Anopheles gambiae str. PEST; Arabidopsis thaliana (Mouse-ear cress); Brachydanio rerio (Zebrafish) (Danio rerio); Caenorhabditis elegans; Candida glabrata CBS138; Capsella rubella; Drosophila melanogaster (Fruit fly); Glycine max (Soybean); Homo sapiens (Human); Kluyveromyces lactis NRRL Y-1140; Mus musculus (Mouse); Neurospora crassa; Oryza sativa (Rice); Oryza sativa (japonica cultivar-group); R. solanacearum; Rattus norvegicus (Rat); Saccharomyces cerevisiae (Baker's yeast); Xenopus laevis (African clawed frog); Yarrowia lipolytica CLIB99;	GALA PROTEIN 1. (Q8XRE0); CG9952-PA (RE01138p) (Partner of Paired). (Q9W214); Putative F-box protein family, AtFBL3 (Putative F-box family proteinAtFBL3). (Q8RWU5); F-box/LRR-repeat protein 14 (F-box and leucine-rich repeat protein14). (Q8BID8); F-box/LRR-repeat protein 14 (F-box and leucine-rich repeat protein14). (Q8N1E6); Hypothetical protein FLJ40218. (Q8N7Y4); Fbl13 protein. (Q6AZS0); Hypothetical protein MGC40195. (Q6TGX5); Mus musculus adult male testis cDNA, RIKEN full-length enrichedlibrary, clone:4921539K22 product:hypothetical F-box domain containingprotein, full insert sequence. (Q8CDU4); GALA PROTEIN 3. (Q8XYF7); F-box and leucine-rich repeat protein 13 transcript variant 2. (Q6UVW7); AgCP9906. (Q7PZW8); FBXL13 protein. (Q8NEE6); Hypothetical protein. (Q6DCN6); F-box protein family, AtFBL4. (Q8LGK0); F-box protein family, AtFBL6 (Hypothetical protein At2g25490). (Q9SKK0); FBXL2 protein. (Q6IAN3); F-box/LRR-repeat protein 2 (F-box and leucine-rich repeat protein 2)(F-box protein FBL2/FBL3). (Q9UKC9); CG8272-PA (LD27656p). (Q9V4Y8); Putative F-box protein family, AtFBL4 (Putative F-box protein familyprotein FBL4) (AT4g15470/dl3775w). (Q9C5D2); Hypothetical protein F7A7_240. (Q9M004); CG14891-PA (GH16156p). (Q9VET4); At2g17020 (At2g17020/At2g17020). (Q9SDA8); Mus musculus adult male testis cDNA, RIKEN full-length enrichedlibrary, clone:4933409A11 product:hypothetical F-box domain containingprotein, full insert sequence. (Q8CDE9); Hypothetical protein zgc:73374. (Q6PBJ7); CG32085-PA. (Q9VTL8); RH06780p. (Q8SXG5); F22K20.10 protein (F-box protein family, AtFBL5). (O49286); Yarrowia lipolytica chromosome C of strain CLIB99 of Yarrowialipolytica. (Q6CBX1); Ubiquitin ligase complex F-box protein GRR1. (P24814); F-box protein (EIN3-binding F-box protein 2). (Q708Y0); F-box and leucine-rich repeat protein 19. (Q6PCT2); F-box/LRR-repeat protein 7 (F-box and leucine-rich repeat protein 7)(F-box protein FBL6/FBL7). (Q9UJT9); Hypothetical protein F4I10.140 (Hypothetical protein AT4g33210). (Q9SMY8); F-box/LRR-repeat protein 20 (F-box and leucine-rich repeat protein 20)(F-box/LRR-repeat protein 2-like). (Q96IG2); F-box/LRR-repeat protein 20 (F-box and leucine-rich repeat protein 20)(F-box/LRR-repeat protein 2-like). (Q9CZV8*); Hypothetical protein FLJ38068. (Q8N1P0); Hypothetical protein AT4g30640. (Q9M096); Hypothetical protein T21F11.10 (Putative F-box family protein,AtFBL14). (Q9M8M4); Hypothetical protein FLJ25911. (Q8N789); MGC64561 protein. (Q7SZ73); CG4221-PA (LD38495p). (Q9VF10); S-phase kinase-associated protein 2 (F-box protein Skp2) (CyclinA/CDK2-associated protein p45) (p45skp2) (F-box/LRR-repeat protein 1). (Q13309); AT19096p. (Q8T437); CG9316-PA. (Q9VIK3); Putative F-box protein. (Q8H8E1); Candida glabrata strain CBS138 chromosome M complete sequence. (Q6FJ52); CG1839-PA (GH11272p). (Q9VY46); Putative F-box protein Fbl2. (Q69X07); Putative F-box protein. (Q6Z796); GALA PROTEIN 5. (Q8XZN8); Similar to Arabidopsis thaliana transport inhibitor response 1 (TIR1) (T48087). (Q9FRB6); F24J8.5 protein (At1g21410/F24J8_17). (Q9LPL4); F-box and leucine-rich repeat protein 13 transcript variant 1. (Q6UVW8); F-box protein AtFBL5. (Q8LDN3); F20N2.2 (At1g55590/F20N2_18). (Q9ZWC6); CG13213-PA (Cg13213-pb). (Q9V5W4); SD01170p. (Q961T2); Hypothetical protein. (Q9ARF5); Similarities with sp|P38285 Saccharomyces cerevisiae YBR158w CST13singleton. (Q6CVS2); Fbxl4 protein. (Q8CIC4); Transport inhibitor response 1 (TIR1) (Putative transport inhibitorresponse TIR1, AtFBL1 protein). (O24660); Grr1. (O22512*); Hypothetical protein B0393.3. (Q17492); CG5003-PA. (Q9VAZ0); Hypothetical protein. (Q7SBQ7); AT3g54650/T5N23_10. (Q8W104); F-box/LRR-repeat protein 20 (F-box and leucine-rich repeat protein 20)(F-box/LRR-repeat protein 2-like). (Q9QZH7*); CG9003-PA. (Q9V605);
F5_F8_type_C~Big_4~Big_4	X. axonopodis (pv. citri); X. campestris (pv. campestris);	Hypothetical protein XAC2534. (Q8PJJ7*); Hypothetical protein XCC2399. (Q8P849*);
FAD_binding_4~FAD-oxidase_C~CCG~CCG	R. solanacearum;	PUTATIVE OXIDOREDUCTASE PROTEIN (EC 1.-.-.-). (Q8Y282);
Fer2~FAD_binding_6	B. japonicum;	Blr7998 protein. (Q89C00);
Fer2~Fer2_2~Ald_Xan_dh_C~Ald_Xan_dh_C2	B. japonicum; Desulfotalea psychrophila LSv54; Desulfovibrio desulfuricans; Desulfovibrio gigas; uncultured bacterium 582;	Blr6161 protein. (Q89H34); Aldehyde oxidoreductase (EC 1.2.-.-) (Molybdenum iron sulfur protein). (Q46509); Probable aldehyde oxidoreductase. (Q6ARM6); Aldehyde oxidoreductase. (Q9REC4); Aldehyde oxidoreductase, putative. (Q6SF55);

Fer4~Fer4~Molybdop_Fe4S4~Molybdopterin~Molydop_binding	X. campestris (pv. campestris);	Formate dehydrogenase related protein. (Q8P7E7);
Fil_haemagg~DUF637	P. syringae (pv. tomato);	Filamentous haemagglutinin family protein. (Q880F2);
Flagellin_IN~FliD_C	B. japonicum;	Blr3696 protein. (Q89NY7);
Flg_bb_rod~FlaE~FlaE~DUF1078	P. syringae (pv. tomato);	Flagellar hook protein FlgE. (Q884Z9);
FliD_N~Flagellin_IN~FliD_C~FliD_C	R. solanacearum;	PROBABLE FLAGELLAR HOOK-ASSOCIATED PROTEIN 2 (FILAMENT CAPPROTEIN). (Q8XST4);
GAF~GAF~GAF~GAF~GAF~GGDEF~EAL	B. japonicum;	Bll7580 protein. (Q89D63);
GAF~GAF~PAC~GAF~HisKA~HATPase_c~Response_reg	M . loti;	Two-component sensor histidine kinase. (Q984C1);
GAF~HisKA_2~HATPase_c	B. japonicum; Methanosarcina mazei (Methanosarcina frisia);	Hypothetical sensory transduction histidine kinase (EC 2.7.3.-). (Q8PT32); Bll6988 protein. (Q89ET4); Blr2180 protein. (Q89T70);
GAF~HisKA~HATPase_c~Response_reg~Response_reg~Response_reg	X. axonopodis (pv. citri); X. campestris (pv. campestris);	Two-component system sensor protein. (Q8PBD7); Two-component system sensor protein. (Q8PMZ3);
GAF~PAC~HWE_HK	M . loti;	Mlr3690 protein. (Q98FN5);
GAF~PAC~PAS~GGDEF	X. axonopodis (pv. citri);	Hypothetical protein XAC4358. (Q8PEJ1);
GAF~PAC~PAS~PAC~GAF~HisKA~HATPase_c~Response_reg	X. campestris (pv. campestris);	Two-component system sensor protein. (Q8P8T6);
GAF~PAC~PAS~PAC~PAS~PAC~PAS~GGDEF	X. axonopodis (pv. citri); X. campestris (pv. campestris);	Histidine kinase/response regulator hybrid protein. (Q8P525); Histidine kinase-response regulator hybrid protein. (Q8PPS5);
GAF~PAS~GGDEF	X. campestris (pv. campestris);	Hypothetical protein XCC4224. (Q8P354);
GAF~PAS~HWE_HK~HATPase_c	S. meliloti;	Hypothetical protein SMc01507. (Q92K06);
GAF~PAS~PAC~PAS~PAC~GAF~HisKA~HATPase_c~Response_reg	X. axonopodis (pv. citri);	Two-component system sensor protein. (Q8PKV3);
GAF~Phytochrome	Bradyrhizobium sp. ORS278; X. axonopodis (pv. citri);	Phytochrome-like protein. (Q8PEQ2); Bacteriophytochrome. (Q8VUB6);
GAF~Phytochrome~HisKA~HATPase_c~HWE_HK	P. syringae (pv. tomato);	Bacteriophytochrome, putative. (Q882H5);

	Aplysia californica (California sea hare); Arabidopsis thaliana (Mouse-ear cress); Ashbya gossypii (Yeast) (Eremothecium gossypii); Aspergillus fumigatus (Sartorya fumigata); Bos taurus (Bovine); Brachydanio rerio (Zebrafish) (Danio rerio); Caenorhabditis elegans; Candida albicans (Yeast); Candida glabrata CBS138; Cavia porcellus (Guinea pig); Debaryomyces hansenii CBS767; Dolichos biflorus (Horse gram); Drosophila melanogaster (Fruit fly); Encephalitozoon cuniculi GB-M1; Gallus gallus (Chicken); Glycine max (Soybean); Glycine soja (Wild soybean); Homo sapiens (Human); Kluyveromyces lactis (Yeast); Kluyveromyces	Yarrowia lipolytica chromosome A of strain CLIB99 of Yarrowialipolytica. (Q6CI77); Hypothetical protein MAL13P1.121. (Q8IE96); Yarrowia lipolytica chromosome C of strain CLIB99 of Yarrowialipolytica. (Q6CBD9); Putative nucleoside-triphosphatase (EC 3.6.1.15) (Nucleosidetriphosphate phosphohydrolase) (NTPase). (P52913); Nucleoside-triphosphatase II precursor (EC 3.6.1.15) (NTPase-II)(Nucleoside triphosphate hydrolase 2). (Q27895*); Nucleoside-triphosphatase I precursor (EC 3.6.1.15) (NTPase-I)(Nucleoside triphosphate hydrolase 1). (Q27893*); NTPase. (O76144*); Nucleoside triphosphate hydrolase. (Q7YZC8); Kluyveromyces lactis strain NRRL Y-1140 chromosome D of strain NRRL Y-1140 of Kluyveromyces lactis. (Q6CQW7); Apyrase (EC 3.6.1.5). (Q70KY5); Similar to CA0704|IPF11806 Candida albicans IPF11806. (Q6BRF5); ADR006Wp. (Q75AB2); Possible guanosine diphosphatase. (Q9BHV5*); Golgi apyrase (EC 3.6.1.5) (ATP-diphosphatase) (Adenosinediphosphatase) (ADPase) (ATP-diphosphohydrolase) (Golgi nucleosidediphosphatase). (P40009); YER005W. (Q6B252); Hypothetical protein (Related to apyrase) (NDPase/NTPase). (Q7SAL4); Ectonucleoside triphosphate diphosphohydrolase 4 (EC 3.6.1.6)(NTPDase4) (Uridine-diphosphatase) (UDPase) (Lysosomal apyrase-likeprotein of 70 kDa). (Q9Y227); Ectonucleoside triphosphate diphosphohydrolase 4 (EC 3.6.1.6)(NTPDase4) (Uridine-diphosphatase) (UDPase) (Lysosomal apyrase-likeprotein of 70 kDa). (Q9DBT4); Zgc:92668. (Q6DH30); Lysosomal apyrase-like protein 1. (Q9NQZ7); LALP1. (Q9ET10); ENTPD4 protein. (Q8NE73); Similar to sp|P40009 Saccharomyces cerevisiae YER005w YND1. (Q6FRC2); Hypothetical protein. (Q7RAN3); Probable guanosine-diphosphatase. (Q9C2M0); Guanosine-diphosphatase, putative (EC 3.6.1.42). (Q6MYR7); Nucleoside-diphosphatase mig-23 (EC 3.6.1.-) (NDPase) (Abnormal cellmigration protein 23). (Q21815*); Probable guanosine-diphosphatase (EC 3.6.1.42) (GDPase). (Q8TGG8); Putative nucleoside phosphatase. (Q8S6I3); Putative nucleosid phosphatase. (Q8LM38); NTPDase-1. (Q6QHT8); Similar to sp|P32621 Saccharomyces cerevisiae YEL042w GDA1. (Q6BU56); Ectonucleoside triphosphate diphosphohydrolase 1 (EC 3.6.1.5) (NTPDase1) (Ecto-ATP diphosphohydrolase) (ATPDase) (Lymphoid cellactivation antigen) (Ecto-apyrase) (CD39 antigen). (Q9MYU4*); Guanosine-diphosphatase (EC 3.6.1.42) (GDPase). (Q8TGH6*); Ynd1 protein (Spcc11e10.05c protein). (Q9USP2); ATP-diphosphohydrolase 1. (Q7YTA4); Yarrowia lipolytica chromosome C of strain CLIB99 of Yarrowialipolytica. (Q6CBK6); Nucleoside triphosphate diphosphohydrolase-8 (EC 3.6.1.5). (Q6UQ22); ATP diphosphohydrolase. (Q8RVT8*); Putative NTPDase. (Q6IEK0); Ectonucleoside triphosphate diphosphohydrolase 3 (EC 3.6.1.5)(NTPDase3) (Ecto-ATP diphosphohydrolase) (ATPDase) (Ecto-apyrase)(CD39 antigen-like 3) (HB6). (O75355); Ectonucleoside triphosphate diphosphohydrolase 1 (EC 3.6.1.5)(NTPDase1) (Ecto-ATP diphosphohydrolase) (ATPDase) (Lymphoid cellactivation antigen) (CD39 antigen) (Ecto-apyrase). (O18956*); Guanosine-diphosphatase (EC 3.6.1.42) (GDPase). (P32621*); Guanosine-diphosphatase (EC 3.6.1.42) (GDPase). (Q9HEM6*); Mus musculus adult male corpora quadrigemina cDNA, RIKEN full-lengthenriched library, clone:B230353O06 product:ectonucleoside triphosphatediphosphohydrolase 3, full insert sequence (Nucleoside triphosphatediphosphohydrolase-3) (EC 3.6.1.5) (Mus musculus 2 days pregnant adultfemale oviduct cDNA, RIKEN full-length enriched library,clone:E230001J21 product:ectonucleoside triphosphatediphosphohydrolase 3, full insert sequence). (Q8BFW6); Nucleoside triphosphate diphosphohydrolase 3. (Q80Z26); Similar to sp|P32621 Saccharomyces cerevisiae YEL042w GDA1. (Q6FLR7); AFR362Cp. (Q753F2); Ectonucleoside triphosphate diphosphohydrolase 1 (EC 3.6.1.5) (NTPDase1) (Ecto-ATP diphosphohydrolase) (ATPDase) (Lymphoid cellactivation antigen) (Ecto-apyrase) (CD39 antigen). (P97687*); Hypothetical protein MGC76207. (Q6NVI9); Ectonucleoside triphosphate diphosphohydrolase 1 (EC 3.6.1.5)(NTPDase1) (Ecto-ATP diphosphohydrolase) (ATPDase) (Lymphoid cellactivation antigen) (Ecto-apyrase) (CD39 antigen). (P55772*); Mus musculus adult male testis cDNA, RIKEN full-length enrichedlibrary, clone:4921511C05 product:ectonucleoside triphosphatediphosphohydrolase 1, full insert sequence. (Q8CDV7); Ectonucleoside triphosphate diphosphohydrolase 1 (EC 3.6.1.5)(NTPDase1) (Ecto-ATP diphosphohydrolase) (ATPDase) (Lymphoid cellactivation antigen) (Ecto-apyrase) (CD39 antigen). (P49961*); Guanosine-diphosphatase (EC 3.6.1.42) (GDPase). (Q9UT35); Entpd1 protein. (Q921Q6*); Ectonucleoside triphosphate

GFO_IDH_MocA~GFO_IDH_MocA_C~Aldo_ket_red	A. tumefaciens ; M . loti; S. meliloti;	Mlr6441 protein. (Q989G0); Putative oxidoreductase. (Q92XJ9); Oxidoreductase. (Q8UB70); AGR_L_3327p. (Q7CRQ4);
GGDEF~EAL~Response_reg	X. axonopodis (pv. citri); X. campestris (pv. campestris);	C-di-GMP phosphodiesterase A. (Q8P9A7); C-di-GMP phosphodiesterase A. (Q8PL14);
GMC_oxred_N~GLF	P. syringae (pv. tomato);	UDP-galactopyranose mutase. (Q881J5);
GXGXG~GXGXG	A. tumefaciens ;	Formyl-methanofuran dehydrogenase. (Q8U871); AGR_L_1266p. (Q7CUD5);
Gamma-BBH	Angiostrongylus cantonensis; Anopheles gambiae str. PEST; Ashbya gossypii (Yeast) (Eremothecium gossypii); Caenorhabditis elegans; Candida glabrata CBS138; Debaryomyces hansenii CBS767; Drosophila melanogaster (Fruit fly); Homo sapiens (Human); Kluyveromyces lactis NRRL Y-1140; Macaca fascicularis (Crab eating macaque) (Cynomolgus monkey); Mus musculus (Mouse); Neurospora crassa; Pichia farinosa (Yeast); Pseudomonas sp. (strain AK-1); Rattus norvegicus (Rat); M . loti; Saccharomyces cerevisiae (Baker's yeast); Xenopus laevis (African clawed frog); Yarrowia lipolytica CLIB99; uncultured bacterium 578;	Similar to sp|P23180 Saccharomyces cerevisiae YHL021c. (Q6FMD9); Hypothetical protein. (Q7S3G2); Hypothetical 53.1 kDa protein in SPO11-OPI1 intergenic region. (P23180); Similar to DEHA0C03839g Debaryomyces hansenii. (Q6C1G9); ADR024Wp. (Q75A94); Similar to CA5280|IPF1899 Candida albicans. (Q6BVE2); Hypothetical protein. (Q9UVG4); Trimethyllysine hydroxylase (EC 1.14.11.8). (Q8NIQ9); Trimethyllysine dioxygenase (EC 1.14.11.8) (Epsilon-trimethyllysine 2-oxoglutarate dioxygenase) (TML-alpha-ketoglutarate dioxygenase) (TMLhydroxylase) (TML dioxygenase) (TMLD). (Q96UB1); AEL035Wp. (Q757P7); Putative gamma-butyrobetaine dioxygenase (EC 1.14.11.1) (Gamma-butyrobetaine,2-oxoglutarate dioxygenase) (Gamma-butyrobetainehydroxylase) (Gamma-BBH). (Q19000); Similar to sp|P23180 Saccharomyces cerevisiae YHL021c singleton. (Q6CQT2); Gamma-butyrobetaine,2-oxoglutarate dioxygenase (EC 1.14.11.1). (Q8MUC4); Similar to ca|CA1591|IPF14663 Candida albicans unknown function. (Q6CX63); Similar to ca|CA1591|IPF14663 Candida albicans IPF14663 unknownfunction. (Q6BLJ0); CG14630-PA. (Q9W5B5); Hypothetical protein. (Q7S7S2); Similar to sp|Q96UB1 Neurospora crassa Trimethyllysinedioxygenase. (Q6CCC7); Hypothetical protein M05D6.7. (Q21526); CG10814-PA (LP01339p). (Q9V6P0); CG5321-PA. (Q9VY24); AgCP6271. (Q7Q783); Gamma-butyrobetaine dioxygenase (EC 1.14.11.1) (Gamma-butyrobetaine,2-oxoglutarate dioxygenase) (Gamma-butyrobetaine hydroxylase) (Gamma-BBH). (O75936); Gamma-butyrobetaine dioxygenase (EC 1.14.11.1) (Gamma-butyrobetaine,2-oxoglutarate dioxygenase) (Gamma-butyrobetaine hydroxylase) (Gamma-BBH). (Q9QZU7); Gamma-butyrobetaine dioxygenase (EC 1.14.11.1) (Gamma-butyrobetaine,2-oxoglutarate dioxygenase) (Gamma-butyrobetaine hydroxylase) (Gamma-BBH). (Q924Y0); Probable gamma-butyrobetaine dioxygenase (EC 1.14.11.1) (Gamma-butyrobetaine,2-oxoglutarate dioxygenase) (Gamma-butyrobetainehydroxylase) (Gamma-BBH). (Q98KK0); MGC68497 protein. (Q6P426); Gamma-butyrobetaine,2-oxoglutarate dioxygenase, putative. (Q6SFT2); TMLHE protein. (Q6IA90); CG4335-PA. (Q9VDM7); Trimethyllysine dioxygenase, mitochondrial precursor (EC 1.14.11.8)(Epsilon-trimethyllysine 2-oxoglutarate dioxygenase) (TML-alpha-ketoglutarate dioxygenase) (TML hydroxylase) (TML dioxygenase) (TMLD). (Q91ZW6); Trimethyllysine dioxygenase, mitochondrial precursor (EC 1.14.11.8) (Epsilon-trimethyllysine 2-oxoglutarate dioxygenase) (TML-alpha-ketoglutarate dioxygenase) (TML hydroxylase) (TML dioxygenase) (TMLD). (Q91ZE0); Trimethyllysine dioxygenase, mitochondrial precursor (EC 1.14.11.8)(Epsilon-trimethyllysine 2-oxoglutarate dioxygenase) (TML-alpha-ketoglutarate dioxygenase) (TML hydroxylase) (TML dioxygenase) (TMLD). (Q9NVH6); Gamma-butyrobetaine dioxygenase (EC 1.14.11.1) (Gamma-butyrobetaine,2-oxoglutarate dioxygenase) (Gamma-butyrobetaine hydroxylase) (Gamma-BBH). (P80193); TMLHE protein. (Q8TBT0); Gamma butyrobetaine hydroxylase. (Q8WNU7); GM16107p. (Q8MSF7);
GerE~TPR_2	M . loti;	Adenylate cyclase. (Q98JC9);
Glutaminase~STAS~cNMP_binding	B. japonicum;	Probable glutaminase 1 (EC 3.5.1.2). (Q89NA7); Probable glutaminase 2 (EC 3.5.1.2). (Q89KV2);
Glutaredoxin~Rhodanese	X. fastidiosa (strain Temecula1 / ATCC 700964);	Glutaredoxin-like protein. (Q87BN9);
Glyco_hydro_18~fn3~fn3~CBM_5_12	Bacillus circulans; Kurthia zopfii; P. syringae (pv. tomato);	Chitinase. (Q9KHB3*); Chitinase. (Q48494*); Chitinase A1 precursor (EC 3.2.1.14). (P20533*); Chitinase. (Q88AJ0);
Glyco_hydro_53~Ricin_B_lectin	E. carotovora subsp. atroseptica SCRI1043;	Putative exported plant proteoglycan hydrolase. (Q6D8W9);

Glyco_hydro_6~CBM_2	Microbispora bispora; Micromonospora cellulolyticum; R. solanacearum; Thermomonospora fusca; X. fastidiosa (strain Temecula1 / ATCC 700964); X. fastidiosa;	Cellulose 1,4-beta-cellobiosidase. (Q87E00); 1,4-beta-cellobiosidase. (Q9PDW2); PROBABLE EXOGLUCANASE A (1,4-BETA-CELLOBIOSIDASE) PROTEIN (EC3.2.1.91). (Q8XS97); Endoglucanase A precursor (EC 3.2.1.4) (Endo-1,4-beta-glucanase) (Cellulase). (P26414*); Endoglucanase E-2 precursor (EC 3.2.1.4) (Endo-1,4-beta-glucanase E-2)(Cellulase E-2) (Cellulase E2). (P26222*); Endo-beta-1,4-glucanase. (Q53488);
Glyco_hydro_6~fn3	X. campestris (pv. campestris);	1,4-beta-cellobiosidase. (Q8P514*); 1,4-beta-cellobiosidase. (Q8P622);
Glyco_transf_28~DUF1205	B. japonicum; E. carotovora subsp. atroseptica SCRI1043; Micromonospora echinospora (Micromonospora purpurea); Micromonospora griseorubida; Micromonospora megalomicea subsp. nigra; Saccharopolyspora erythraea (Streptomyces erythraeus); Saccharopolyspora spinosa; Streptomyces antibioticus; Streptomyces argillaceus; Streptomyces cyanogenus; Streptomyces fradiae; Streptomyces galilaeus; Streptomyces globisporus; Streptomyces griseus subsp. griseus; Streptomyces halstedii; Streptomyces nanchangensis; Streptomyces narbonensis; Streptomyces nogalater; Streptomyces peucetius; Streptomyces purpurascens; Streptomyces rishiriensis; Streptomyces rochei (Streptomyces parvullus); Streptomyces sp. (strain C5); Streptomyces sp. AM-7161; Streptomyces venezuelae; Streptomyces violaceoruber; Streptomyces violaceus (Streptomyces venezuelae);	Glycosyltransferase. (Q70J68); Glycosyltransferase. (Q9RQR5); Glycosyl transferase. (Q9RP99); Glycosyltransferase. (Q70J80); Glycosyl transferase homolog. (Q9ZGC0); Putative glycosyl transferase. (Q9ZA43); NanG5. (Q7WTE9); Probable NDP-forosamyltransferase. (Q9ALN7); AclK. (Q8VWB7); AknK. (Q9L555); Glycosyl transferase. (Q9RPA2); Putative baumycin biosynthesis protein. (P95834); Glycosyltransferase. (Q87830); Putative glycosyl transferase. (Q9RN63); Putative glycosyl transferase. (Q9L4U6); RhoG. (Q8RS24); Glycosyl transferase. (Q54824); Glycosyltransferase. (Q53881); Glycosyl transferase. (Q9ZGH7); Glycosyltransferase. (Q83WE1); TDP-desosamine glycosyltransferase. (Q9F826); NbmD. (Q8KRX7); Glycosyltransferase. (P95747); TDP-megosamine glycosyltransferase. (Q9F839); Glycosyltransferase. (O54224); RdmH. (Q935Z8); Putative glycosyltransferase. (Q83X73); Desosaminyltransferase. (O33935); Glycosyltransferase. (Q76KZ6); Glycosyltransferase. (O87831); Glycosyl transferase homolog. (Q9ZGB1); Putative glycosyltransferase. (Q83X64); TDP-mycarose glycosyltransferase. (Q9F832); EryBV (Putative glycosyltransferase). (O33939); Glycosyltransferase LndGT4. (Q6T1C7); Glycosyltransferase. (Q70J70); Glycosyltransferase. (O87479*); Putative glycosyltransferase. (Q6D1R8); Glycosyl transferase. (Q9F8U7*); ORF5; putative. (Q56176); CalG1. (Q8KNF2); Glycosyltransferase. (Q70J67); Glycosyltransferase. (O87480*); C-glycosyl transferase. (Q7WT14); Glycosyl transferase. (Q9RPA1); Glycosyl transferase homolog. (Q9ZGB8*); Glycosyl transferase. (Q8GMG8); Bll2692 protein. (Q89RS0);
Glyco_transf_36	Clostridium thermocellum; Neurospora crassa; X. axonopodis (pv. citri); X. campestris (pv. campestris);	NdvB protein. (Q8P3J4); NdvB protein. (Q8PEZ3); Cellodextrin phosphorylase. (Q93HT8); Cellodextrin phosphorylase. (O24780); Hypothetical protein. (Q7S0S2);
Glycos_transf_1~ABC2_membrane	B. japonicum;	Bll4316 protein. (Q89M77);
Glycos_transf_2~Chitin_synth_2	Azorhizobium caulinodans; B. japonicum; Bradyrhizobium sp. (strain SNU001); Bradyrhizobium sp. ORS285; Bradyrhizobium sp. WM9; Mesorhizobium sp. (strain 7653R); Rhizobium etli; Rhizobium galegae; Rhizobium leguminosarum (biovar viciae); M . loti; S. meliloti; Rhizobium sp. (strain N33); Rhizobium sp. (strain NGR234); Rhizobium sp. SIN-1; Rhizobium tropici; Wautersia taiwanensis;	NodC. (Q8GNH5); NodC. (O52478); N-acetylglucosaminyltransferase (EC 2.4.1.-) (Nodulation protein C). (P72334*); N-acetylglucosaminyltransferase (EC 2.4.1.-) (Nodulation protein C). (P04341); Rhizobium meliloti nodulation genes nodA, nodB and nodC. (Q52971); N-acetylglucosaminyltransferase (EC 2.4.1.-) (Nodulation protein C). (P17862); N-acetylglucosaminyl transferase. (Q70YC2); Nodulation N-acetylglucosaminyltransferase NodC. (Q9AQ23*); N-acetylglucosaminyltransferase (EC 2.4.1.-) (Nodulation protein C). (P26024); PROBABLE NODULATION PROTEIN C, N-ACETYLGLUCOSAMINYLTRANSFERASE. (Q8KJI5); Common nodulation protein C, NodC (N-acetylglucosaminyltransferase). (Q8KLG3); Nodulation protein NodC. (Q93U10); NODC=POLYSACCHARIDE POLYMERIZING enzyme homolog. (Q9R614); N-acetylglucosaminyltransferase (EC 2.4.1.-) (Nodulation protein C). (P04340); N-acetylglucosaminyltransferase (EC 2.4.1.-) (Nodulation protein C). (P50357); N-acetylglucosaminyltransferase (EC 2.4.1.-) (Nodulation protein C). (P50356*); NodC. (Q6PTX8); NodC. (Q9Z3I6); NodC protein. (Q53254); N-acetylglucosaminyltransferase (EC 2.4.1.-) (Nodulation protein C). (Q07755*); NodC protein. (Q9RAN5*);
Glycos_transf_2~Glyco_transf_25	M . loti;	Mll0582 protein. (Q98MH1);
Glycos_transf_2~NodS~Polysacc_deac_1	M . loti;	Mll3280 protein. (Q98GK8);
Glyoxalase~Abhydrolase_2	S. meliloti;	Hypothetical protein SMc01956. (Q92K03);
Glyoxalase~Acetyltransf_1	P. aeruginosa;	Hypothetical protein. (Q9HZ97);
Glyphos_transf~Glycos_transf_2	P. aeruginosa;	Hypothetical protein. (Q9I4N8);
GntR~GntR~FCD	R. solanacearum;	PROBABLE TRANSCRIPTION REGULATOR PROTEIN. (Q8XSY9);

Guanylate_cyc~DUF323	B. japonicum;	Bll6746 protein. (Q89FF4);
Guanylate_cyc~TPR_1~TPR_2	B. japonicum; M . loti; S. meliloti;	Bll7664 protein. (Q89CY1); Probable adenylate cyclase protein (EC 4.6.1.1). (Q92W27); Adenylate cyclase; Cya3. (Q98EW6);
Guanylate_cyc~TPR_1~TPR_2~TPR_1~TPR_2	B. japonicum;	Blr6986 protein. (Q89ET6);
Guanylate_cyc~TPR_2~TPR_1	B. japonicum; M . loti; S. meliloti;	Blr7791 protein. (Q89CK6); PROBABLE ADENYLATE/GUANYLATE CYCLASE PROTEIN (EC 4.6.1.1). (Q92M76); Adenylate cyclase; Cya3. (Q98GL1);
Guanylate_cyc~TPR_2~TPR_4	B. japonicum; S. meliloti;	Putative adenylate cyclase protein (EC 4.6.1.1). (Q92WN9); Bll5043 protein. (Q89K68);
Guanylate_cyc~TPR_2~TPR_4~TPR_2	B. japonicum;	Bll5958 protein. (Q89HN2);
Guanylate_cyc~TPR_4	B. japonicum;	Bll7877 protein. (Q89CC0);
HAMP~Cache~HAMP~MCPsignal	A. tumefaciens ;	Methyl-accepting chemotaxis protein. (Q8UIC4); AGR_C_652p. (Q7D1L0);
HAMP~GAF~Guanylate_cyc	B. japonicum;	Blr0359 protein. (Q89XF5);
HAMP~Guanylate_cyc~TPR_2	B. japonicum;	Bll4769 protein. (Q89KX9);
HAMP~HAMP~HAMP~HAMP~HAMP~HAMP~HAMP~HAMP~GAF~HisKA~HATPase_c~Response_reg~Response_reg	M . loti;	Probable sensory histidine kinase. (Q98II7);
HAMP~HAMP~HAMP~HAMP~HAMP~HAMP~HAMP~HAMP~HAMP~HAMP~HAMP~GAF~HisKA~HATPase_c~Response_reg~Response_reg~Response_reg	B. japonicum;	Two-component hybrid sensor and regulator. (Q89S13);
HAMP~PAC~HWE_HK	M . loti;	Histidine protein kinase. (Q98P62);
HAMP~PAC~PAC~PAS~PAC~GGDEF~EAL	R. solanacearum;	PROBABLE TRANSMEMBRANE PROTEIN. (Q8XT61*);
HAMP~PAS~PAC~PAS~PAS~PAC~HisKA~HATPase_c~Response_reg	B. japonicum;	Two-component hybrid sensor and regulator. (Q89XC9);
HEAT~TPR_2	P. syringae (pv. tomato);	TPR domain protein. (Q880Q2);
HEM4~HemX	R. solanacearum;	PROBABLE BIFUNCTIONAL: UROPORPHYRIN-III C-METHYLTRANSFERASE ANDUROPORPHYRINOGEN-III SYNTHASE TRANSMEMBRANE PROTEIN (EC 2.1.1.107) (EC4.2.1.75). (Q8XWW4);
HIM~HIM~HIM~HIM~HIM~HIM~HIM~HIM~Hep_Hag~HIM~HIM~Hep_Hag~HIM~YadA	R. solanacearum;	PUTATIVE HEMAGGLUTININ-RELATED PROTEIN. (Q8XPL8);
HSDR_N~KAP_NTPase	P. syringae (pv. tomato);	Hypothetical protein. (Q87WC6);
HTH_1~Glycos_trans_3N~Glycos_transf_3	A. tumefaciens ;	Hypothetical protein Atu3917. (Q8U913); AGR_L_1856p. (Q7CTM2);
HTH_3~SBP_bac_7	M . loti;	Mll9374 protein. (Q981H4);
HTH_AraC~HTH_AraC~FmdA_AmdA	B. japonicum;	Transcriptional regulatory protein. (Q89LS7);
Haemagg_act~Fil_haemagg~DUF637	P. aeruginosa; P. syringae (pv. tomato);	Filamentous hemagglutinin, intein-containing, putative. (Q880E1); Probable hemagglutinin. (Q9I791*);
Haemagg_act~Fil_haemagg~DUF637~DUF1020	P. aeruginosa;	Hypothetical protein. (Q9I120*);
Haemagg_act~Glug~Glug~Glug~Glug~Glug~Glug~Glug~Glug~Glug~Glug~Glug~Glug~Glug	P. aeruginosa;	Adhesive protein CupB5. (Q9HWU6);
Haemagg_act~Glug~Glug~Glug~Glug~Glug~Glug~Glug~Glug~Glug~Glug~Glug~Glug~Glug~Glug~Glug~Glug~Glug	P. aeruginosa;	Hypothetical protein. (Q9HVN6);
Harpin	Erwinia amylovora; E. carotovora subsp. atroseptica SCRI1043; Erwinia chrysanthemi; Erwinia pyrifoliae; Erwinia stewartii; Pantoea agglomerans (pv. gypsophilae) (Erwinia herbicola); Pectobacterium atrosepticum; Pectobacterium carotovorum (subsp. carotovorum) (E. carotovora (subsp. carotovora));	Harpin (HrpNEp). (Q6XDB9); Harpin (Harpin-EA). (Q01099); HrpN. (Q6XDB7); HrpN. (Q9FCY8); Harpin. (Q6D5D7); HrpN. (Q6RK43); Harpin. (Q9KH45); HrpN. (Q6WEH4); Harpin protein. (Q9EXP0); Harpin (Harpin-ECH). (Q47278); Harpin. (Q700A0);
Helicase_C~HA2	P. syringae (pv. tomato);	ATP-dependent helicase HrpB, putative. (Q87W33);

HemY_N~TPR_2~TPR_4	R. solanacearum;	Hypothetical protein hemY. (Q8XWW5*);
HemolysinCabind~Cadherin~Cadherin~Cadherin~Cadherin~HemolysinCabind	B. japonicum;	Bll3714 protein. (Q89NW9);
HemolysinCabind~Glyco_hydro_16	A. tumefaciens ; M . loti; S. meliloti;	Endo-1,3-1,4-beta-glycanase. (Q8UA11); AGR_L_2532p. (Q7CSS8); Endo-1,3-1,4-beta-glycanase exsH (EC 3.2.1.-) (Succinoglycanbiosynthesis protein exsH). (O33680); Endo-1,3-1,4-beta-glycanase eglC (EC 3.2.1.-) (Succinoglycanbiosynthesis protein eglC). (Q9Z3Q2); Endo-1,3-1,4-beta-glycanase. (Q98I38);
HemolysinCabind~HCBP_related~HemolysinCabind	R. solanacearum;	PUTATIVE HEMOLYSIN-TYPE CALCIUM-BINDING PROTEIN. (Q8Y377);
HemolysinCabind~HCBP_related~HemolysinCabind~HCBP_related~HCBP_related~HemolysinCabind~HCBP_related~HemolysinCabind~HCBP_related~HemolysinCabind~HCBP_related~HemolysinCabind~HCBP_related~HemolysinCabind	M . loti;	Rhizobiocin; RzcA. (Q98LG7);
HemolysinCabind~HCBP_related~HemolysinCabind~HCBP_related~He_PIG~He_PIG~HemolysinCabind	X. axonopodis (pv. citri);	Hemolysin-type calcium binding protein. (Q8PKH6);
HemolysinCabind~HCBP_related~HemolysinCabind~HCBP_related~HemolysinCabind	X. fastidiosa (strain Temecula1 / ATCC 700964); X. fastidiosa;	Bacteriocin. (Q87BM1); Hemolysin-type calcium binding protein. (Q9PFI9);
HemolysinCabind~HCBP_related~HemolysinCabind~HCBP_related~HemolysinCabind~HCBP_related~HemolysinCabind	X. fastidiosa (strain Temecula1 / ATCC 700964); X. fastidiosa;	Hemolysin-type calcium binding protein. (Q87EK2); Hemolysin-type calcium binding protein. (Q9PEL7);
HemolysinCabind~HCBP_related~HemolysinCabind~HCBP_related~HemolysinCabind~HCBP_related~HemolysinCabind~HCBP_related	X. fastidiosa;	Hemolysin-type calcium binding protein. (Q9P9W1);
HemolysinCabind~HCBP_related~HemolysinCabind~HCBP_related~HemolysinCabind~HCBP_related~HemolysinCabind~HCBP_related~HemolysinCabind	X. fastidiosa (strain Temecula1 / ATCC 700964);	Hemolysin-type calcium binding protein. (Q87BF0);
HemolysinCabind~HCBP_related~HemolysinCabind~HCBP_related~HemolysinCabind~HCBP_related~HemolysinCabind~HCBP_related~HemolysinCabind~HCBP_related~HemolysinCabind	R. solanacearum;	PUTATIVE CALCIUM BINDING HEMOLYSIN PROTEIN. (Q8Y2T6);
HemolysinCabind~HCBP_related~HemolysinCabind~HCBP_related~HemolysinCabind~HCBP_related~HemolysinCabind~HCBP_related~HemolysinCabind~HCBP_related~HemolysinCabind~HCBP_related~HemolysinCabind	X. fastidiosa;	Bacteriocin. (Q9PAT8);
HemolysinCabind~He_PIG~HemolysinCabind	P. syringae (pv. tomato); P. syringae;	PsmE. (Q6EBW9); Mannuronan C-5-epimerase, putative. (Q87XU1);
HemolysinCabind~PPC~HemolysinCabind	S. meliloti;	Putative outer membrane secretion protein. (Q92UV3);
Hep_Hag~HIM~HIM~HIM~HIM~HIM~HIM~Hep_Hag~HIM~Hep_Hag~HIM~YadA	X. fastidiosa (strain Temecula1 / ATCC 700964);	Surface protein. (Q87D62);
Hep_Hag~HIM~HIM~HIM~HIM~HIM~HIM~YadA	S. meliloti;	Hypothetical protein SMc01708. (Q92KQ7);
Hep_Hag~HIM~HIM~HIM~HIM~HIM~Hep_Hag~HIM~Hep_Hag~HIM~YadA	X. fastidiosa;	Surface protein. (Q9PC04);
Hep_Hag~HIM~HIM~Hep_Hag~HIM	B. japonicum;	Blr5538 protein. (Q89IU6);
Hep_Hag~HIM~HIM~X_fast-SP_rel~HIM~HIM~X_fast-SP_rel~HIM~HIM~X_fast-SP_rel~HIM~HIM~X_fast-SP_rel~HIM~HIM~X_fast-SP_rel~HIM~HIM~HIM~Hep_Hag~HIM~Hep_Hag~HIM~YadA	X. fastidiosa;	Surface protein. (Q9PD50);
Hep_Hag~HIM~Hep_Hag~HIM~Hep_Hag~HIM~Hep_Hag~HIM~HIM~YadA	X. fastidiosa (strain Temecula1 / ATCC 700964); X. fastidiosa;	Outer membrane protein XadA. (Q87DF4); Surface-exposed outer membrane protein. (Q9PD63);

Hep_Hag~HIM~Hep_Hag~HIM~Hep_Hag~HIM~Hep_Hag~HIM~Hep_Hag~HIM~Hep_Hag~HIM~Hep_Hag~HIM~HIM~YadA	M . loti;	Mll2848 protein. (Q98HJ2);
Hep_Hag~HIM~Hep_Hag~HIM~Hep_Hag~HIM~Hep_Hag~HIM~Hep_Hag~HIM~Hep_Hag~HIM~Hep_Hag~HIM~YadA	X. axonopodis (pv. citri); X. campestris (pv. campestris);	Outer membrane protein. (Q8PCQ5); Outer membrane protein. (Q8PGS0);
Hep_Hag~HIM~Hep_Hag~HIM~Hep_Hag~HIM~YadA	X. axonopodis (pv. citri);	Outer membrane protein. (Q8PGR8);
Het-C~Het-C	P. syringae (pv. tomato);	Conserved domain protein. (Q87WJ0);
Hexapep~FdtA	P. syringae (pv. tomato); X. campestris (pv. campestris);	Lipopolysaccharide biosynthesis protein. (Q888F6); Bifunctional acetyl transferase/isomerase. (Q8PCT8); Putative bifunctional enzyme WxcM. (Q93S92);
HisKA~HATPase_c~Response_reg~Response_reg~GerE	A. tumefaciens ;	Two component sensor kinase/response regulator hybrid. (Q8U6K2); AGR_L_153glp. (Q7CVX1);
HisKA~HisKA~Response_reg~Guanylate_cyc	B. japonicum;	Two-component hybrid sensor and regulator. (Q89SW2);
HisKA~Response_reg~Guanylate_cyc	S. meliloti;	Putative regulatory protein. (Q92WJ5);
Hpt~Hpt~Hpt~Hpt~H-kinase_dim~HATPase_c~CheW~Response_reg	X. axonopodis (pv. citri); X. campestris (pv. campestris); X. fastidiosa (strain Temecula1 / ATCC 700964); X. fastidiosa;	PilL protein. (Q8P6P6); PilL protein. (Q8PI00); Chemotaxis-related protein kinase. (Q87D38); Chemotaxis-related protein kinase. (Q9PC33);
Hpt~Hpt~Hpt~Hpt~Hpt~Hpt~HATPase_c~CheW~Response_reg	P. syringae (pv. tomato);	Sensor histidine kinase/response regulator. (Q87VA9);
Hpt~Hpt~Hpt~Hpt~Hpt~Hpt~Hpt~HATPase_c~CheW~Response_reg	P. aeruginosa;	Still frameshift probable component of chemotactic signal transductionsystem. (Q9I696); ChpA. (O87001);
HrpE	Erwinia amylovora; E. carotovora subsp. atroseptica SCRI1043; Erwinia chrysanthemi; Erwinia stewartii; Pectobacterium atrosepticum; Pectobacterium carotovorum (subsp. carotovorum) (E. carotovora (subsp. carotovora)); Pseudomonas fluorescens; P. syringae (pv. glycinea); P. syringae (pv. phaseolicola); P. syringae (pv. savastanoi); P. syringae (pv. syringae); P. syringae (pv. tabaci); P. syringae (pv. tomato); P. syringae;	Type III secretion protein. (Q6D5E3); HrpE. (Q8KUL7); HrpE. (Q6WEG8); HrpE. (Q6RK37); HrpE. (Q9FCZ4); HrpE. (Q46622); HrpE. (Q9F0A7*); Putative hrp type III secretion pathway protein. (O87461*); HrpE. (Q60243); HrpE. (Q6BEP1); Type III secretion protein HrpE. (Q887C4); HrpE. (Q6XDA7); RspE. (Q93PY3); HrpE. (Q6UDQ7); HrpE. (Q52478); HrpE. (Q6QQC0);
HrpF	Erwinia amylovora; E. carotovora subsp. atroseptica SCRI1043; Erwinia chrysanthemi; Erwinia pyrifoliae; Erwinia stewartii; Pectobacterium atrosepticum; Pectobacterium carotovorum (subsp. carotovorum) (E. carotovora (subsp. carotovora)); P. syringae (pv. glycinea); P. syringae (pv. phaseolicola); P. syringae (pv. savastanoi); P. syringae (pv. syringae); P. syringae (pv. tabaci); P. syringae (pv. tomato);	HrpF. (P94765); Type III secretion protein HrpF. (Q887C3); HrpF. (Q60244); HrpF. (O87462); HrpF. (Q9FCZ3); HrpF. (O87439); HrpF. (Q46623); HrpF. (Q6BEP0); Type III secretion protein. (Q6D5E2); HrpF. (Q6XDA6); HrpF. (Q6WEG9); HrpF. (Q6RK38); HrpF. (Q6QQC1); HrpF. (Q6QPK3);

HrpZ	Pseudomonas ficuserectae; P. syringae (pv. actinidiae); P. syringae (pv. glycinea); P. syringae (pv. lachrymans); P. syringae (pv. maculicola); P. syringae (pv. phaseolicola); P. syringae (pv. pisi); P. syringae (pv. sesami); P. syringae (pv. syringae); P. syringae (pv. tabaci); P. syringae (pv. tomato); P. syringae pv. aceris; P. syringae pv. aptata; P. syringae pv. coronafaciens; P. syringae pv. delphinii; P. syringae pv. dendropanacis; P. syringae pv. eriobotryae; P. syringae pv. japonica; P. syringae pv. lapsa; P. syringae pv. magnoliae; P. syringae pv. mori; P. syringae pv. morsprunorum; P. syringae pv. myricae; P. syringae pv. oryzae; P. syringae pv. striafaciens; P. syringae pv. theae; P. syringae;	Harpin protein HrpZ. (Q6L8U9); Harpin protein HrpZ. (Q6L8U5); Harpin protein HrpZ. (Q6L8V3); Harpin protein HrpZ. (Q6L8V7); Type III helper protein HrpZ(Pto). (Q887C6); Harpin protein HrpZ. (Q6L905); Harpin protein HrpZ. (Q6L901); HrpZ. (Q52474); HrpZ. (Q7WTS0); Harpin protein HrpZ. (Q6L8Y9); Harpin protein HrpZ. (Q6L8Z7); Harpin protein HrpZ. (Q6L8Y5); Harpin protein HrpZ. (Q6L8Z3); Harpin protein HrpZ. (Q6L929); Harpin protein HrpZ. (Q6L945); Harpin protein HrpZ. (Q6L957); Harpin protein HrpZ. (Q6L953); Harpin protein HrpZ. (Q6L941); Harpin protein HrpZ. (Q6L933); Harpin protein HrpZ. (Q6L937); HrpZ. (Q9F0B0); HrpZ protein. (Q52481); HrpZ. (Q6UDR1); Harpin protein HrpZ. (Q6L961); Harpin elicitor. (O31180); Harpin. (O87653); Harpin protein HrpZ. (Q6L8X7); Harpin protein HrpZ. (Q6L8X3); Frp. (Q9R733); Harpin protein HrpZ. (Q6L8W9); Frp protein. (Q9R2T7); Frp protein. (Q9Z3U2); Harpin protein HrpZ. (Q7WUA3); Harpin protein HrpZ. (Q6L8W1); Harpin-PSS. (P35674); Harpin protein HrpZ. (Q6L8W5); Harpin protein HrpZ. (Q6L925); Harpin protein HrpZ. (Q6L921); Harpin protein HrpZ. (Q6L917); Harpin protein HrpZ. (Q6L913); Harpin protein HrpZ. (Q6L909); Harpin protein HrpZ. (Q9AJD5);
Ice_nucleation	Bordetella phage BPP-1; Erwinia herbicola; Pantoea ananas (Erwinia uredovora); Pseudomonas fluorescens; P. syringae (pv. syringae); P. syringae; X. campestris (pv. campestris); X. campestris (pv. translucens);	Ice nucleation protein. (P18127); Ice nucleation protein inaA. (P20469); Ice nucleation protein. (Q8PD38); Ice protein. (Q93N36); Ice nucleation protein. (P16239); Ice nucleation protein. (O33479); Ice nucleation protein. (P06620); Ice nucleation protein. (P09815); Ice nucleation protein. (O30611); Ice nucleation protein inaU. (Q47879); Bbp36. (Q775A9);
IclR~SMP-30	A. tumefaciens ; S. meliloti;	Transcriptional regulator, IclR family/regucalcin. (Q8UJL2); Putative calcium binding transcriptional regulatory protein. (Q92ZR8); AGR_pAT_685p. (Q7D306);
Ion_trans_2~cNMP_binding	B. japonicum; Oryza sativa (japonica cultivar-group); Paramecium tetraurelia; M . loti;	K+ channel, putative. (Q6BGF5); K+ channel, putative. (Q6BGA9); OSJNBb0050O03.14 protein. (Q7XT08); Blr7856 protein. (Q89CE1); Mll3241 protein. (Q98GN8*);
Kazal_2~Kazal_2	Drosophila melanogaster (Fruit fly); Mus musculus (Mouse); Phytophthora infestans (Potato late blight fungus); Rattus norvegicus (Rat); M . loti; Rhodnius prolixus (Triatomid bug);	Enhancer of split M1 protein precursor (E(spl)m1). (Q97176*); Follistatin-related protein 3 precursor (Follistatin-like 3)(Follistatin-related gene protein). (Q99PW7*); Follistatin-related protein 3 precursor (Follistatin-like 3)(Follistatin-related gene protein). (Q9EQC7*); Kazal-like serine protease inhibitor EPI2. (Q6PQH1); Thrombin inhibitor rhodniin. (Q06684); SD09502p. (Q960B5*); CG5392-PA. (Q9VUJ2*); Kazal-like serine protease inhibitor EPI1. (Q6PQH2); Mll2655 protein. (Q98HY5);
Kazal_2~Kazal_2~Kazal_2	Canis familiaris (Dog); Homo sapiens (Human); Mus musculus (Mouse); S. meliloti;	HYPOTHETICAL LIPOPROTEIN TRANSMEMBRANE. (Q92K80); Reversion-inducing cysteine-rich protein with Kazal motifs precursor(mRECK). (Q9Z0J1*); Reversion-inducing cysteine-rich protein with Kazal motifs precursor(hRECK) (Suppressor of tumorigenicity 15) (ST15). (O95980*); Reversion-inducing-cysteine-rich protein with Kasal motifs. (Q7YS79*);

Kelch_2~Kelch_1	Brachydanio rerio (Zebrafish) (Danio rerio); Candida glabrata CBS138; Debaryomyces hansenii CBS767; Dictyostelium discoideum (Slime mold); Emericella nidulans (Aspergillus nidulans); Homo sapiens (Human); Mus musculus (Mouse); Neurospora crassa; Plasmodium falciparum (isolate 3D7); Rattus norvegicus (Rat); Saccharomyces cerevisiae (Baker's yeast); X. axonopodis (pv. citri); Yarrowia lipolytica CLIB99;	Peas. (Q91XU6); Mus musculus adult male liver cDNA, RIKEN full-length enrichedlibrary, clone:1300011D16 product:INTERCELLULAR MEDIATOR, full insertsequence. (Q9DBG8); Testis intracellular mediator protein (PEAS). (Q9BQ90); Hypothetical protein. (Q6AYI2); Kelch domain containing 3 (Peas). (Q8VEM9); Hypothetical protein. (Q99JH9); KLHDC3 protein. (Q96GH7); Hypothetical protein FLJ40400. (Q8N7S8); Rab9 effector p40. (Q9BWB1); P40. (O00568); Rab9 effector p40. (Q6P092); RAB9P40 protein. (Q6IBG7); Kelch-domain protein. (Q703G6); Rab9 effector p40. (Q7Z6M1); Ring canal kelch-like protein. (Q8PEV4*); Hypothetical protein. (Q7SI30); Kelch repeats protein 2. (P50090); Zgc:91813. (Q6GQN7); Mus musculus adult male urinary bladder cDNA, RIKEN full-lengthenriched library, clone:9530020D24 product:hypothetical Galactoseoxidase, central domain structure containing protein, full insertsequence. (Q8BZG7); Zgc:85727. (Q6NUZ4); Rab9 effector p40. (Q8VCH5); RAB9P40 protein. (Q86Y76); YHR158C. (Q6B2C7); Kelch repeats protein 1. (P38853); Mus musculus 0 day neonate eyeball cDNA, RIKEN full-length enrichedlibrary, clone:E130314D10 product:hypothetical Kelch repeat containingprotein, full insert sequence. (Q8BPK7); Ring finger protein B (Protein rngB). (Q7M3S9); Debaryomyces hansenii chromosome A of strain CBS767 of Debaryomyceshansenii. (Q6BY21); Hypothetical protein. (Q8II48); Candida glabrata strain CBS138 chromosome I complete sequence. (Q6FR02); Similarity. (Q6CHD1); Hypothetical protein. (Q7S4F5); Similarity. (Q6C2P9); Full-length cDNA clone CS0DI007YN11 of Placenta of Homo sapiens(human). (Q86TY9);
LRR_1~Fic	X. campestris (pv. campestris);	Leucin rich protein. (Q8P7P6);

LRR_1~Pkinase_Tyr	Arabidopsis thaliana (Mouse-ear cress); Daucus carota (Carrot); Dictyostelium discoideum (Slime mold); Glycine max (Soybean); Homo sapiens (Human); Lotus japonicus; Lycopersicon esculentum (Tomato); Lymnaea stagnalis (Great pond snail); Medicago sativa (Alfalfa); Medicago truncatula (Barrel medic); Melilotus alba (White sweet clover); Neurospora crassa; Oryza sativa (Rice); Oryza sativa (japonica cultivar-group); Pisum sativum (Garden pea); P. aeruginosa; P. syringae (pv. tomato); Rattus norvegicus (Rat); Sorghum bicolor (Sorghum) (Sorghum vulgare); Vicia hirsuta; Zea mays (Maize);	OSJNBb0045P24.8 protein. (Q7XWN3*); Putative leucine rich repeat-type serine/threonine receptor-likekinase. (Q75N53); Receptor kinase-like protein. (O24435*); Putative receptor-like protein kinase. (O22938*); F20B17.5. (Q9MA13); Putative LRR receptor-like kinase 2. (Q6ZIW9); Receptor-like kinase RHG1. (Q8L3Y5); Hypothetical protein At1g79620/F20B17_5. (Q8GY50); OSJNBa0070M12.3 protein (OSJNBa0088H09.21 protein). (Q7X616*); Similarity to receptor protein kinase. (Q9LK43*); Putative receptor protein kinase. (Q75KZ6); Putative receptor-like protein kinase. (Q9ZVD4); Receptor protein kinase-like. (Q9LT95*); Putative LRR receptor protein kinase. (Q9SIX4*); Receptor-like protein kinase (At5g16590). (Q9FMD7*); Similar to Dictyostelium discoideum (Slime mold). Pats1 (Hypotheticalprotein roco2). (Q8SSS9); Putative receptor-like protein kinase. (O22178*); Putative brassinosteroid LRR receptor kinase protein. (Q7F0P5); Transmembrane protein kinase. (Q7XBA0*); Putative neurotrophin receptor LTRK 1 precursor (EC 2.7.1.112). (O76997*); Hypothetical protein F17O14.15. (Q9C9Y8*); Hypothetical protein roco11. (Q6XHA5); Leucine rich repeat receptor kinase-like protein (At4g22730). (O49654*); High affinity nerve growth factor receptor precursor (EC 2.7.1.112)(TRK1 transforming tyrosine kinase protein) (p140-TrkA) (Trk-A). (P04629*); High affinity nerve growth factor receptor precursor (EC 2.7.1.112)(p140-TrkA) (Slow nerve growth factor receptor) (Trk-A). (P35739*); Putative receptor protein kinase. (Q942J7); Putative leucine-rich repeat transmembrane protein kinase 1. (Q94HG1*); Putative receptor kinase. (Q9FK63*); Receptor-like kinase. (Q6QNV0); Putative receptor kinase (ESTs AU032341 (R3918)). (Q9LDG0*); Putative receptor-like protein kinase. (Q852J5); Receptor-like protein kinase (Putative receptor protein kinase). (Q9LVN2*); Receptor-like protein kinase. (Q9FJP1*); Putative somatic embryogenesis receptor kinase. (Q6Z8R5); Putative receptor protein kinase. (Q84WP2); Putative receptor kinase-like protein. (Q8VYT3*); F12M16.30. (Q9MAG1); Receptor-like kinase SYMRK. (Q8LKX1*); Putative receptor-like protein kinase. (Q9SLD0*); Receptor protein kinase, putative. (Q9C9N5*); Nodulation receptor kinase. (Q8L6F3*); Nodulation receptor kinase. (Q8L6A4*); SYM19. (Q8LKZ1*); Nodulation receptor kinase. (Q8L6F2*); F27J15.13. (Q9M9B0*); SYMRK. (Q8LKZ0*); Nodulation receptor kinase. (Q8L4H4*); Nodulation receptor kinase. (Q8L6L0*); Receptor-like protein kinase. (Q9FK65*); Nodulation receptor kinase. (Q8L6K5*); Putative receptor-like protein kinase. (Q9ZQC2); Receptor-like kinase homolog. (Q9M0A8*); Putative kinase-like protein TMKL1 precursor. (P33543*); Putative receptor-like protein kinase. (O64505*); Nodulation receptor kinase. (Q8L6F1*); Putative receptor kinase. (Q8H6J3); Putative receptor kinase. (Q8LJX8*); Probable protein kinase-like. (Q6EQM5); Serine/threonine-specific receptor protein kinase-like. (Q6ERN3); Serine/threonine-specific receptor protein kinase-like. (Q6ES01); Nodulation receptor kinase. (Q8L6K6*); OsD305. (Q948R2); Nodulation receptor kinase. (Q8L6K7*); Putative leucine-rich repeat receptor kinase (Putative receptor-likeprotein kinase). (Q75GV0); F14L17.16 protein. (Q9M9S4*); Receptor-like protein kinase. (O64397*); Receptor protein kinase-like protein. (Q9M1L7*); Receptor-like protein kinase. (O64398*); Putative leucine-rich repeat/receptor protein kinase. (Q6Z3S1); Putative receptor-like kinase RHG1. (Q6YW99); Putative receptor-like protein kinase 3. (Q69K60); Putative receptor-like protein kinase PRK1. (Q6H504); Putative receptor kinase. (Q9ASD8*); Hypothetical protein T14L22.12. (Q9FZA8*); Receptor-like protein kinase 1. (Q9AUC2*); Similarity to receptor-like protein kinase. (Q9FMW1*); Receptor-like protein kinase-like protein. (Q8LBQ5*); Putative leucine-rich repeat transmembrane protein kinase. (Q84JF8); Receptor protein kinase. (Q9LJY0*); T7N9.25. (O04567*); F11F12.7 protein (Protein kinase, putative) (Putative receptor-likeprotein kinase) (At1g50610). (Q9LPT1*); Leucine-rich repeat transmembrane protein kinase 1, putative; 10414-7611. (Q9C8M8*); Receptor kinase-like protein. (Q9XEV0*); Protein kinase, putative. (Q880J6); Putative receptor protein kinase. (Q8GY10); Putative receptor-like kinase RHG1. (Q6ZL12); F2K11.19. (Q9SH29*); OSJNBb0020O11.17 protein. (Q7XU76); OJ991113_30.7 protein. (Q7XUF9*); F20P5.27 protein. (O04545*); AT3g08680/F17O14_15. (Q93ZK0*); Putative receptor kinase. (Q8GYR5); Receptor kinase-like protein. (Q9FLL2*); Receptor-like protein kinase-like protein (Putative receptorkinase). (Q9LSI9*); Similar to Arabidopsis thaliana chromosome 4 BAC clone F28A21 ;putative protein

LacI~LacI~Peripla_BP_1	E. carotovora subsp. atroseptica SCRI1043;	LacI-family transcriptional regulator. (Q6D0M6);
Lipase_GDSL~Cupin_2	X. axonopodis (pv. citri); X. campestris (pv. campestris);	Rhamnogalacturonan acetylesterase. (Q8PE29); Rhamnogalacturonan acetylesterase. (Q8PQZ8);
Lipoprotein_5~Peptidase_S8~Autotransporter	X. axonopodis (pv. citri); X. campestris (pv. campestris);	Serine protease. (Q8PKM7*); Serine protease. (Q8P941);
Lipoprotein_9~Lipoprotein_9	R. solanacearum;	PUTATIVE OUTERMEMBRANE SIGNAL PEPTIDE PROTEIN. (Q8XU08*);
MAAL_N	M . loti;	Msl6020 protein. (Q98AF7);
MASE1~PAC~PAC~PAC~PAC~HisKA~HATPase_c	S. meliloti;	Putative two-component sensor histidine kinase protein (EC 2.7.3.-). (Q92UG9*);
MASE1~PAC~PAC~PAS~PAC~HisKA~HATPase_c	M . loti;	Two component sensor-kinase. (Q98LK8);
MASE1~PAS~PAC~PAC~PAS~GGDEF~EAL	P. aeruginosa;	Hypothetical protein. (Q9I4F7);
MASE2~Guanylate_cyc	P. aeruginosa;	Probable adenylate cyclase. (Q9HZ23);
MCE~MCE	Desulfotalea psychrophila LSv54; E. carotovora subsp. atroseptica SCRI1043;	Hypothetical protein. (Q6D4C9); Hypothetical protein. (Q6AQF6);
MHYT~PAC~GGDEF~EAL	M . loti;	Mlr3504 protein. (Q98G38*);
MHYT~PAC~HisKA~HATPase_c~Response_reg	B. japonicum;	Two-component hybrid sensor and regulator. (Q89C13);
MLTD_N~SLT	P. syringae (pv. tomato);	Hypothetical protein (Membrane-bound lytic murein transglycosylase D,putative). (Q9JP38);
MOSC~FAD_binding_6~NAD_binding_1~Fer2	B. japonicum;	Bll3766 protein. (Q89NR8);
MR_MLE_N	Agrobacterium rhizogenes; Methanopyrus kandleri; S. meliloti;	Riorf25 protein. (Q9F5G7); Putative dehydratase. (Q931B9); O-succinylbenzoate-synthase-related enzyme. (Q8TWI8);
MR_MLE_N~MR_MLE~MR_MLE	Burkholderia cepacia (Pseudomonas cepacia); R. solanacearum;	Putative chloromuconate cycloisomerase. (O34027); PROBABLE GLUCARATE DEHYDRATASE PROTEIN (EC 4.2.1.40). (Q8XRK2);
MTTB	Methanosarcina acetivorans; Methanosarcina barkeri; Methanosarcina mazei (Methanosarcina frisia); M . loti; S. meliloti; uncultured bacterium 581;	Trimethylamine methyltransferase; MttB. (Q98BV7); Trimethylamine methyltransferase; MttB. (Q98K37); Trimethylamine methyltransferase; MttB. (Q98KE4); Putative methyltransferase. (Q92YQ8); Mlr1212 protein. (Q98L24); PUTATIVE TRIMETHYLAMINE METHYLTRANSFERASE PROTEIN. (Q92P20); Mll1230 protein. (Q98L11); PUTATIVE METHYLTRANSFERASE PROTEIN. (Q92ND5); Mlr8280 protein. (Q983L0); Hypothetical protein SMc00886. (Q92RP1); Trimethylamine methyltransferase mttB1 (EC 2.1.1.-) (TMAmethyltransferase 1). (Q8TTA9); Trimethylamine methyltransferase mttB (EC 2.1.1.-) (TMAmethyltransferase). (O93658); Trimethylamine methyltransferase mttB2 (EC 2.1.1.-) (TMAmethyltransferase 2). (P58974); Trimethylamine methyltransferase mttB2 (EC 2.1.1.-) (TMAmethyltransferase 2). (Q8TS73); Trimethylamine methyltransferase mttB1 (EC 2.1.1.-) (TMAmethyltransferase 1). (P58973); Methyltransferase, putative. (Q6SFA2); Putative membrane protein, similar to methyltransferases. (Q92V70);
Met_synt_B12~Met_synt_B12	M . loti;	Mll6206 protein. (Q98A09);
Metallophos~HemolysinCabind~HCBP_related~HemolysinCabind	A. tumefaciens ;	Rhizobiocin/RTX toxin. (Q8U7N7); AGR_L_909p. (Q7CUW1);
Microcin	Aeromonas hydrophila; Escherichia coli; Escherichia fergusonii; Hafnia alvei; Plasmid ColIb-P9; P. aeruginosa; Shigella sonnei;	Alveicin B immunity protein. (Q6WRW2); Alveicin A immunity protein. (Q6WRW9); Colicin E1* immunity protein (ImmE1) (Microcin E1* immunity protein). (P22558); Colicin E1 immunity protein. (Q8GH11); Colicin E1 immunity protein (ImmE1) (Microcin E1 immunity protein). (P33637); Colicin E1 immunity protein. (Q7B9M7); 12.5 kDa polypeptide. (O87672); Colicin E1 immunity protein (ImmE1) (Microcin E1 immunity protein). (P02985); Colicin immunity protein. (Q9I4Y5); Colicin immunity protein. (Q7WXX3); Immunity protein. (Q46741); Immunity protein for colicin Ib. (Q7DJZ5); Immunity protein for colicin Ia. (P08701); Colicin Ia immunity protein. (Q7B9M2); Immunity protein for colicin Ib. (P08702*); Immunity protein. (Q46739); Colicin Ib immunity protein. (Q8G8S2);

MmoB_DmpM	Acinetobacter calcoaceticus; Acinetobacter sp; Alcaligenes eutrophus (Ralstonia eutropha); B. japonicum; Burkholderia cepacia (Pseudomonas cepacia); Burkholderia kururiensis; Burkholderia pickettii (Pseudomonas pickettii); Comamonas testosteroni (Pseudomonas testosteroni); Methylococcus capsulatus; Methylocystis sp. M; Methylocystis sp. WI14; Methylomonas sp. KSPIII; Methylomonas sp. KSWIII; Methylosinus trichosporium; Mycobacterium rhodesiae; P. aeruginosa; Pseudomonas butanovora; Pseudomonas mendocina; Pseudomonas putida; Pseudomonas sp; Pseudomonas sp. (strain CF600); Pseudomonas sp. KL28; Pseudomonas stutzeri (Pseudomonas perfectomarina); Ralstonia sp. E2; Ralstonia sp. KN1; Rhodococcus corallinus; Rhodococcus sp. AD45; Sulfolobus solfataricus; Xanthobacter sp. (strain Py2);	Methane monooxygenase regulatory protein B. (P18797); Soluble methane monooxygenase regulatory protein B (MMOB). (Q7DJP5); Soluble methane monooxygenase regulatory protein B (MMOB). (Q9R2V8); Methane monooxygenase regulatory protein B. (P27356); Soluble methane monooxygenase protein B. (O06118); MmoB. (Q9RAM1); Butane monooxygenase regulatory protein. (Q8KQE8); Toluene-4-monooxygenase system protein D. (TmoD). (Q97YS7); Toluene-4-monooxygenase system protein D (EC 1.14.13.-). (Q00459); TbhD. (O07071); Putative effector/coupling protein. (Q9RBN9); Toluene-3-monooxygenase ferredoxin protein (Effector subunit). (Q51942); TouD protein (Toluene o-xylene monooxygenase component). (O87801); Coupling/effector protein. (Q9ZET4); Putative hydroxylase component. (O69181); Tbc2D monooxygenase. (Q9EZN8); BmoD1 protein. (P95411); Phenol hydroxylase component. (O84960); Phenol hydroxylase component (Phenol hydroxylase subunit). (Q9ZNP5); Coupling protein. (Q53026); TbmC protein. (Q52571); DMS oxygenase component. (O32430); Blr3680 protein. (Q89P03); Subunit of phenolhydroxylase. (Q52163); Phenol hydroxylase. (Q52172); TomA2. (Q9ANX3); Phenol hydroxylase component. (Q9RAF7); Phenolhydroxylase component. (Q43980); Phenol hydroxylase P2 protein (EC 1.14.13.7) (Phenol 2-monooxygenaseP2 component). (P19731); CrpC. (O30591); Tbc1C monooxygenase. (Q7BJZ9); Putative alkene monooxygenase coupling/effector protein. (Q6XBI7); Phenol hydroxylase subunit PhkC. (Q8VUU0); Phenol hydroxylase component. (Q7WTJ5); LapM. (Q7WYF2); Butylphenol hydroxylase component A3. (Q842G4); Phenol hydroxylase component phM. (Q84AQ3);
Mn_catalase~DUF892	B. japonicum;	Bll3758 protein. (Q89NS6);
MobA_MobL~Viral_helicase1	A. tumefaciens ; A. tumefaciens;	Conjugation protein. (Q8U6F2); AGR_L_66p. (Q7CW18); Conjugal transfer protein. (Q8KRE4);
NHL~SdiA-regulated	P. aeruginosa;	Hypothetical protein. (Q9I6H2);
NTP_transf_2~PolyA_pol~HD~ACT~ACT	X. axonopodis (pv. citri); X. campestris (pv. campestris);	[Protein-PII] uridylyltransferase (EC 2.7.7.59) (PII uridylyl-transferase) (Uridylyl removing enzyme) (UTase). (Q8PAU4); [Protein-PII] uridylyltransferase (EC 2.7.7.59) (PII uridylyl-transferase) (Uridylyl removing enzyme) (UTase). (Q8PMJ8);
NTP_transferase~MannoseP_isomer~GlcNAc_2-epim	M . loti; S. meliloti;	Phosphomannose isomerase. (Q985M7); Probable mannose-6-phosphate isomerase, GDP-mannose pyrophosphorylaseprotein (EC 2.7.7.22). (Q92VN8);
NUDIX~TMP-TENI	X. fastidiosa (strain Temecula1 / ATCC 700964); X. fastidiosa;	DGTP-pyrophosphohydrolase. (Q87EA6); Bifunctional DGTP-pyrophosphohydrolase/thiamine phosphatesynthase. (Q9PEA8);
NeuB~Antifreeze~AP_endonuc_2	B. japonicum;	Blr5971 protein. (Q89HL9);

NodA	Azorhizobium caulinodans; Azorhizobium sp. SD02; Azorhizobium sp. SG05; Bradyrhizobium elkanii; B. japonicum; Bradyrhizobium sp. (strain ANU 289); Bradyrhizobium sp. (strain NC92); Bradyrhizobium sp. ARC403; Bradyrhizobium sp. CBP70; Bradyrhizobium sp. CBP90; Bradyrhizobium sp. CCT6220; Bradyrhizobium sp. D1; Bradyrhizobium sp. Genista10; Bradyrhizobium sp. LL13; Bradyrhizobium sp. ORS130; Bradyrhizobium sp. ORS135; Bradyrhizobium sp. ORS138; Bradyrhizobium sp. ORS166; Bradyrhizobium sp. ORS170; Bradyrhizobium sp. ORS1810; Bradyrhizobium sp. ORS1812; Bradyrhizobium sp. ORS1816; Bradyrhizobium sp. ORS1832; Bradyrhizobium sp. ORS1838; Bradyrhizobium sp. ORS1844; Bradyrhizobium sp. ORS1896; Bradyrhizobium sp. ORS285; Bradyrhizobium sp. ORS287; Bradyrhizobium sp. ORS352; Bradyrhizobium sp. ORS364; Bradyrhizobium sp. ORS524; Bradyrhizobium sp. ORS88; Bradyrhizobium sp. ORS938; Bradyrhizobium sp. Osaka6; Bradyrhizobium sp. USDA 3001; Bradyrhizobium sp. USDA 3139; Bradyrhizobium sp. USDA 3152A; Bradyrhizobium sp. USDA 3475; Bradyrhizobium sp. USDA 3505; Bradyrhizobium sp. USDA 3517; Bradyrhizobium sp. WM9; Bradyrhizobium sp. WU425; Burkholderia tuberum; Mesorhizobium ciceri; Mesorhizobium huakuii; Mesorhizobium mediterraneum; Mesorhizobium plurifarium; Mesorhizobium sp. (strain 7653R); Methylobacterium nodulans; Rhizobium etli; Rhizobium fredii (Sinorhizobium fredii); Rhizobium galegae; Rhizobium giardinii bv. giardinii; Rhizobium huautlense; Rhizobium leguminosarum (biovar trifolii); Rhizobium leguminosarum (biovar viciae); M . loti; S. meliloti; Rhizobium mongolense; Rhizobium sp. (strain N33); Rhizobium sp. (strain NGR234); Rhizobium sp. SIN-1; Rhizobium sp. STM251; Rhizobium sp. STM270; Rhizobium tropici; Rhizobium undicola; Sinorhizobium medicae; Sinorhizobium saheli bv. sesbaniae; Sinorhizobium sp. BR816; Sinorhizobium sp. ORS1044; Sinorhizobium sp. ORS1085; Sinorhizobium sp. ORS1230; Sinorhizobium sp. ORS1873; Sinorhizobium sp. RAT902; Sinorhizobium terangae bv. acaciae; Sinorhizobium terangae bv. sesbaniae; Sinorhizobium terangae; Wautersia taiwanensis;	Putative acyltransferase. (Q70ZX1); PROBABLE NODA N-ACYLTRANSFERASE PROTEIN. (Q8KJI4); NodA protein. (Q711Y7); NodA protein. (Q711Y5); Putative acyltransferase. (Q70ZX7); Putative acyltransferase. (Q70ZV9); Putative acyltransferase. (Q70ZW1); Putative acyltransferase. (Q70ZW3); Putative acyltransferase. (Q70ZW5); Nodulation protein A (EC 2.3.1.-). (Q52839); Putative acyltransferase. (Q70ZW7); Putative acyltransferase. (Q70ZY1); Nodulation protein A (EC 2.3.1.-). (Q9F0C9); Putative acyltransferase. (Q70ZW9); Nodulation protein A (EC 2.3.1.-). (P04674); Nodulation protein A (EC 2.3.1.-). (P50347); Nodulation protein A (EC 2.3.1.-). (Q9AMZ0); Nodulation protein A (EC 2.3.1.-). (P50326); Nodulation protein A. (Q7AUU9); Nodulation protein A. (Q7AUU8); Nodulation protein NodA. (Q93U12); Nodulation protein A. (Q7AUQ6); Putative acyltransferase. (Q70ZX3); NodA protein. (Q711W6); NodA protein. (Q711W4); NodA protein. (Q711W2); NodA protein. (Q711W0); Putative acyltransferase. (Q70ZV5); Putative acyltransferase. (Q70ZV7); Putative acyltransferase. (Q70ZX5); NodA protein. (Q711Y3); NodA protein. (Q711V8); NodA protein. (Q711V6); Putative acyltransferase. (Q70ZY2); NodA protein. (Q711X0); Putative acyltransferase. (Q70ZT5); NodA protein. (Q711W8); Nodulation N-acyltransferase NodA. (Q9AQ25); Putative acyltransferase. (Q70ZT7); Putative acyltransferase. (Q70ZT9); NodA protein. (Q93L89); Putative acyltransferase. (Q70ZU1); Putative acyltransferase. (Q70ZU3); Putative acyltransferase. (Q70ZU5); Putative acyltransferase. (Q70ZU7); Putative acyltransferase. (Q70ZX9); Putative acyltransferase. (Q70ZU9); Putative acyltransferase. (Q70ZV1); Putative acyltransferase. (Q70ZV3); NodA. (Q8GNH3); Nodulation protein A (EC 2.3.1.-). (Q53252); Nodulation protein A (EC 2.3.1.-). (P50349); Nodulation protein A (EC 2.3.1.-). (P72329); Nodulation protein A (EC 2.3.1.-). (P02962); Nodulation protein A (EC 2.3.1.-). (P04338); Nodulation protein A (EC 2.3.1.-). (P04673); Nodulation protein A (EC 2.3.1.-). (P50348); NodA protein. (Q712B1); Nodulation protein A (EC 2.3.1.-). (Q9RAN9); Nodulation protein A (EC 2.3.1.-). (Q07739); NodA protein. (Q712A9); NodA protein. (Q712A7); NodA protein. (Q712A5); NodA protein. (Q711Z9); NodA protein. (Q711Z5); NodA protein. (Q711Z3); NodA protein. (Q8VVG9); NodA protein. (Q711Z1); NodA protein. (Q711Y9); NodA protein. (Q711Y1); NodA protein. (Q711X9); NodA protein. (Q711V4); NodA protein. (Q711V2); NodA protein. (Q711V0); NodA protein. (Q711U6); Acyltransferase. (Q70YB4); NodA protein. (Q8VVH0); Nodulation protein A (EC 2.3.1.-). (Q8VVH1); Nodulation protein A (EC 2.3.1.-). (Q8VVF0); NodA acyltransferase. (Q6EX53); NodA protein. (Q712D2); NodA protein. (Q712D0); NodA protein. (Q712C6); NodA protein. (Q712C0); NodA protein. (Q712B8); NodA protein. (Q712B6); NodA protein. (Q712B3); Nodulation protein A (EC 2.3.1.-). (P24154); NodA. (Q6PTY0); NodA. (Q8KLI3); Hypothetical protein. (Q6EX56); Msr8756 protein. (Q98A33); ORF3 protein. (Q79AK7);

NodZ	Azorhizobium caulinodans; B. japonicum; Bradyrhizobium sp. WM9; Rhizobium etli; Rhizobium fredii (Sinorhizobium fredii); M . loti; Rhizobium sp. (strain NGR234);	Nodulation protein Z (EC 2.4.1.-). (P55355); Nodulation protein Z (EC 2.4.1.-). (Q45271); Nodulation protein NodZ. (O85712); Nodulation fucosyltransferase NodZ. (Q9AQ17); Nodulation protein; NodZ. (Q98AU6); PROBABLE FUCOSYLTRANSFERASE (NODULATION PROTEIN NODZ). (Q8KJ52); Probable fucosyl transferase protein, NodZ. (Q8KLG7); Nodulation protein Z (EC 2.4.1.-). (Q43966); Msr8743 protein. (Q98A36);
NolV	B. japonicum; Rhizobium etli; Rhizobium fredii (Sinorhizobium fredii); M . loti; Rhizobium sp. (strain NGR234);	Nodulation protein nolV. (P55716); Nodulation protein NolV. (Q93LZ0); Nodulation protein; NolV. (Q989P3); Nodulation protein. (O79US1); NolV. (Q9ANH3); Hypothetical protein nolV. (Q8KKY6); Nodulation protein nolV. (P33211); Hypothetical 7.1 kDa protein in nolU-nolV intergenic region (ORF4). (P33214);
NolX	R. solanacearum; Rhizobium fredii (Sinorhizobium fredii); M . loti; Rhizobium sp. (strain NGR234); X. axonopodis (pv. citri); X. axonopodis pv. glycines; X. campestris (pv. campestris); X. campestris (pv. vesicatoria); Xanthomonas oryzae (pv. oryzae);	HrpF protein. (Q8PBA6); HrpF protein. (Q8PQD2); HrpF. (Q83XD5); HrpF. (O33967); HrpF. (Q6F5A9); HrpF. (Q9KW22); Type III secretion system component. (Q6QJ83); SECRETED PROTEIN POPF2. (Q8XRF4); SECRETED PROTEIN POPF1. (Q8XPT2); Nodulation protein; NolX. (Q989P8); Nodulation protein nolX. (P55711); Nodulation protein NolX. (Q93LZ2); Nodulation protein NolX. (Q9EUG7); Nodulation protein nolX. (P33213);
Oxidored_nitro~Fer2_BFD	B. japonicum;	Nitrogenase iron-molybdenum cofactor biosynthesis protein nifE. (P26506);
PAC~GAF~GGDEF	E. carotovora subsp. atroseptica SCRI1043;	Putative exported protein. (Q6D226);
PAC~GAF~PAS~PAC~HisKA~HATPase_c~Response_reg	P. aeruginosa;	Probable sensor/response regulator hybrid. (Q9I495);
PAC~HATPase_c	X. campestris (pv. campestris);	Histidine kinase/response regulator hybrid protein. (Q8P912);
PAC~PAC~GGDEF~EAL	M . loti;	Mll1848 protein. (Q98JP5);
PAC~PAC~HWE_HK	M . loti;	Two component system sensor histidine kinase. (Q988F6);
PAC~PAC~PAS~PAC~PAC~HisKA~HATPase_c	A. tumefaciens ;	Two component sensor kinase. (Q8U7Z4); AGR_L_1117p. (Q7CUK9);
PAC~PAS~HisKA~HATPase_c	P. aeruginosa;	Probable two-component sensor. (Q9HWA7);
PAC~PAS~PAC~PAS~HisKA~HATPase_c~Response_reg	R. solanacearum;	PROBABLE COMPOSITE TWO-COMPONENT REGULATORY (SENSOR KINASE ANDRESPONSE REGULATOR HYBRID) TRANSCRIPTION REGULATOR PROTEIN. (Q8XPG5);
PAC~PAS~PAC~PAS~PAC~GGDEF	M . loti;	Mll3491 protein. (Q98G48);
PAC~PAS~PAC~PAS~PAC~HWE_HK	A. tumefaciens ;	Two component sensor kinase. (Q8UJE9); AGR_pAT_788p. (Q7D2U9);
PAC~PAS~PAC~PAS~PAC~PAC~MCPsignal	P. syringae (pv. tomato);	Methyl-accepting chemotaxis protein. (Q883C7);
PALP~OCD_Mu_crystall	P. syringae (pv. tomato);	Pyridoxal-phosphate dependent enzyme family/ornithine cyclodeaminasefamily protein. (Q881D4);
PAN~PAN	Caenorhabditis elegans; Phytophthora parasitica (Potato buckeye rot agent); M . loti;	Hypothetical protein R07A4.4. (Q21782); Hypothetical protein C29E6.1 (Let-653 protein). (Q27394*); CBEL protein, formerly GP34 precursor. (O42830*); Hypothetical protein F32B4.8. (O62201); Hypothetical protein ZC449.2. (Q23327*); Hypothetical protein H42K12.3. (O17347*); Hypothetical protein C16D9.1. (Q22902*); Mlr8222 protein. (Q983Q7); Hypothetical protein F52C9.5 in chromosome III. (Q10125*);
PAN~PAN~PAN	Boophilus microplus (Cattle tick); Caenorhabditis elegans; M . loti;	Hypothetical protein F41A4.1. (O61834*); Hypothetical protein ZC449.1. (Q23328*); Hypothetical protein T26C5.2. (Q22815*); Hypothetical protein C30H6.5. (O45277); Mll9162 protein. (Q982A3*); Antigen B membrane protein. (Q8I6X5); Mll9167 protein. (Q982A0*);
PAN~TPR_2~A2M_N~A2M_N_2	A. tumefaciens ; S. meliloti;	Hypothetical protein Atu3695. (Q8U9N1*); Hypothetical protein SMb21298. (Q92VA6*); AGR_L_2284p. (Q7CT39);
PAN~TPR_2~A2M_N~A2M_N_2~TPR_2	M . loti;	Mlr1663 protein. (Q98K29*);
PAP2~FA_desaturase	P. syringae (pv. tomato);	Fatty acid desaturase. (Q881R5);
PAPS_reduct~PAPS_reduct	X. axonopodis (pv. citri);	Phage-related protein. (Q8PK90);
PAS~GAF~PAS~GGDEF~EAL	R. solanacearum;	Hypothetical protein RSc0588. (Q8Y1V1);
PAS~GAF~Phytochrome~PAS	X. campestris (pv. campestris);	Phytochrome-like protein. (Q8P3C2);

PAS~PAC~GAF~GGDEF~EAL	Desulfotalea psychrophila LSv54; X. axonopodis (pv. citri); X. campestris (pv. campestris);	C-di-GMP phosphodiesterase A. (Q8P9J8); C-di-GMP phosphodiesterase A. (Q8PLB5); Hypothetical membrane protein. (Q6AIJ1);
PAS~PAC~PAC~GGDEF	Desulfotalea psychrophila LSv54; R. solanacearum;	Hypothetical protein RSp1203. (Q8XQL9); Hypothetical protein. (Q6AJ00);
PAS~PAC~PAC~HisKA_3~HATPase_c	P. aeruginosa;	Probable two-component sensor. (Q9I5T8*);
PAS~PAC~PAC~HisKA~HATPase_c~Response_reg~Response_reg	A. tumefaciens ;	Two component sensor kinase/response regulator hybrid. (Q8U8P3); AGR_L_1617p. (Q7CTY1);
PAS~PAC~PAC~PAC~GGDEF~EAL	X. axonopodis (pv. citri); X. campestris (pv. campestris);	Hypothetical protein XCC1959. (Q8P9A5); Hypothetical protein XAC1993. (Q8PL12);
PAS~PAC~PAC~PAC~PAC~HisKA~HATPase_c	B. japonicum;	Two-component hybrid sensor and regulator. (Q89KX5); NwsA. (Q06857);
PAS~PAC~PAC~PAS~PAC~MCPsignal	X. axonopodis (pv. citri); X. campestris (pv. campestris);	Chemotaxis protein. (Q8P9W8); Chemotaxis protein. (Q8PLP7);
PAS~PAC~PAS~GGDEF	X. campestris (pv. campestris);	GGDEF family protein. (Q8P4E9);
PAS~PAC~PAS~HisKA~HATPase_c~Response_reg	B. japonicum; Desulfotalea psychrophila LSv54;	Two-component hybrid sensor and regulator. (Q89IG2); Related to two-component system sensory/regulatory protein (Ntrfamily). (Q6ALX1);
PAS~PAC~PAS~PAC~GAF~GGDEF	E. carotovora subsp. atroseptica SCRI1043;	Hypothetical protein. (Q6D123);
PAS~PAC~PAS~PAC~GAF~GGDEF~EAL	B. japonicum;	Bll6545 protein. (Q89G03);
PAS~PAC~PAS~PAC~GAF~PAC~PAC~HWE_HK	S. meliloti;	Putative two-component sensor histidine kinase protein. (Q92UP1); ExsG protein. (O54064);
PAS~PAC~PAS~PAC~HisKA_2~HATPase_c	A. tumefaciens ;	Two component sensor kinase. (Q8UJU8); AGR_pAT_543p. (Q7D385);
PAS~PAC~PAS~PAC~PAS~PAC~HWE_HK	M . loti;	Sensor histidine kinase of two-component. (Q98P10);
PAS~PAC~PAS~PAC~PAS~PAC~PAC~HisKA~HATPase_c	B. japonicum;	Nodulation protein V (EC 2.7.3.-). (P15939);
PAS~PAC~PAS~PAC~PAS~PAC~PAS~PAC~PAS~PAC~HWE_HK	S. meliloti;	Putative sensory transduction histidine kinase. (Q930Y7);
PAS~PAC~PAS~PAS~PAC~PAS~PAC~HisKA~HATPase_c~Response_reg~Response_reg	M . loti;	Hybrid sensory histidine kinase. (Q98FL1);
PAS~PAS~HisKA_3~HATPase_c	Erwinia amylovora; E. carotovora subsp. atroseptica SCRI1043; Pectobacterium atrosepticum; X. axonopodis (pv. citri); X. campestris (pv. campestris);	Sensor kinase HrpX. (Q9X3S8); HrpX related protein. (Q8P9A6); HrpX related protein. (Q8PL13); HrpX. (Q6RK29); Two-component sensor kinase. (Q6D5F2);
PAS~PAS~HisKA~HATPase_c~Response_reg~Response_reg	A. tumefaciens ;	Two component sensor kinase/response regulator hybrid. (Q8UCZ6); AGR_C_4237p. (Q7CXB2);
PAS~PAS~PAC~PAS~PAC~GGDEF~EAL	B. japonicum;	Bll7107 protein. (Q89EH8);
PAS~PAS~PAC~PAS~PAC~PAS~PAC~PAS~PAC~GGDEF~EAL	S. meliloti;	Hypothetical protein. (Q92YN0);
PAS~PAS~PAS~GerE	P. aeruginosa;	Probable transcriptional regulator. (Q9I603);
PAS~PAS~PAS~HisKA~HATPase_c~Response_reg	M . loti;	Sensor kinase. (Q98M23);

PAX	Acropora millepora (Coral); Brachydanio rerio (Zebrafish) (Danio rerio); Branchiostoma floridae (Florida lancelet) (Amphioxus); Caenorhabditis briggsae; Caenorhabditis elegans; Canis familiaris (Dog); Ciona intestinalis; Coturnix coturnix (Common quail); Drosophila melanogaster (Fruit fly); Fugu rubripes (Japanese pufferfish) (Takifugu rubripes); Gallus gallus (Chicken); Halocynthia roretzi (Sea squirt); Homo sapiens (Human); Hydra littoralis (swiftwater hydra); Lampetra japonica (Japanese lamprey) (Entosphenus japonicus); Mus musculus (Mouse); Paracentrotus lividus (Common sea urchin); Phallusia mammilata; Ptychodera flava; Rattus norvegicus (Rat); Rhizobium etli; M . loti; Strongylocentrotus purpuratus (Purple sea urchin); Xenopus laevis (African clawed frog);	Pax6 protein isoform. (Q8VHH9); Paired box protein. (Q8VDB6); Transcription factor. (Q9N9G7); Paired box pox-neuro protein (Paired box neuronal protein). (P23758); Paired box protein. (O16801); Hypothetical protein K06B9.5. (Q21263); Mus musculus 12 days embryo embryonic body between diaphragm regionand neck cDNA, RIKEN full-length enriched library, clone:9430070E09product:paired box gene 9, full insert sequence. (Q8BS81); Hypothetical protein C04G2.7 (PAX protein). (Q17627); Pax-2/5/8. (Q8IAC6); CG11049-PC. (Q8IM96); Pax-A. (O02015); Transcription factor. (Q9N9G8); Transcription factor Pax-A. (O61609); Pax19 related. (O96092); HrPax-258 protein. (O76121); PAX-1 PRODUCT=SCLEROTOME development regulator/DNA-bindingtranscriptional activator. (Q9PSA2); Paired box protein. (Q9DGR0); Pax19. (O96090); Paired box protein. (Q9YH95); Paired box protein. (O57676); Paired box protein. (O57682); Pax9b (Pax9 protein). (Q98866); Pax-2 protein. (Q9PUK6); Paired box protein. (O57677); Transcription factor. (O93370); Paired box protein. (O16800); Pax19 related. (Q9UAH7); Pax-8 DNA-binding transcription factor. (Q9PUK5); Paired box protein. (Q9YI50); Paired box protein. (O57680); Paired box protein. (O57685); Paired box protein. (O16802); Paired-box containing protein Pax-2. (Q9PTX1); Paired box protein. (O57684); Pax9. (Q9DDW3); Pax19 related. (O96091); Pax19 related. (O96095); Pax19. (Q9Y1W7); Pax (Paired box) transcription factor protein 1. (Q21272); Pax-5 protein. (Q9W601); Paired box gene. (O57678); Transcription factor. (O93373); Paired box protein. (O57679); Pax19 related. (O96094); Paired box protein. (O57681); Pax9a. (Q98865); Pax19 related. (O96093); Hypothetical protein. (Q6AZQ6); Pax protein. (Q6TQD4); BSAP splice variant delta7. (Q6S732); Paired box pox-meso protein (Paired box mesodermal protein). (P23757); Paired box protein Pax-9. (P47242); Paired box protein Pax-9. (P55771); Paired box protein Pax-8. (P51974); Paired box protein Pax-8. (Q00288); Paired box protein Pax-8. (Q06710); Paired box protein Pax-8. (P47240); Paired box protein Pax-5 (B-cell specific transcription factor)(BSAP). (Q02650); Paired box protein Pax-5 (B-cell specific transcription factor)(BSAP). (Q02548); Paired box protein Pax-2. (P32114); Paired box protein Pax-2. (Q02962); Paired box protein Pax-2a (Pax[Zf-b]) (No isthmus protein). (Q90268); Paired box protein Pax-1. (P09084); Paired box protein Pax-1 (HUP48). (P15863); BSAP splice variant delta8. (Q6S731); BSAP splice variant delta9. (Q6S730); BSAP splice variant delta78. (Q6S729); AmphiPax-1. (Q17132); BSAP splice variant delta789. (Q6S728); Paired box gene 1. (Q6NTC0); MGC68430 protein. (Q7SZU4); CG11049-PA (Sparkling protein). (O16117); Hypothetical protein F48B9.5. (Q20551); Paired box protein. (Q8VDB4); Hypothetical protein F21D12.5. (Q19677); CG9610-PB. (Q8INR1); Paired box protein. (Q8VBY9); Hypothetical protein yi17-I. (Q8KL19); Hypothetical protein T14G12.1. (Q22509); Hypothetical protein C52D10.5. (Q966N7); Hypothetical protein C27H2.1. (O45267); Hypothetical protein F26H9.3. (P91852); Hypothetical protein W04G5.1. (O62393); Hypothetical protein R13.2. (Q21972); Hypothetical protein Y92H12A.3. (Q9BKW5); Transposase. (Q989K7); Hypothetical protein C09G9.7. (Q17876); Pax6 protein. (Q8VBZ1);
PCMT~Erythro_esteras	B. japonicum; S. meliloti; Rhizobium sp. (strain NGR234);	Bll4651 protein. (Q89L97); Hypothetical fusion protein. (Q92X74); Protein-L-isoaspartate O-methyltransferase (EC 2.1.1.77). (Q6W168);
PD40~F5_F8_type_C	R. solanacearum;	PROBABLE SIGNAL PEPTIDE PROTEIN. (Q8XT36);
PGI~PGI	R. solanacearum;	Hypothetical protein RSc2137. (Q8XXH7);
PHZA_PHZB	E. carotovora subsp. atroseptica SCRI1043; Pantoea agglomerans; P. aeruginosa; Pseudomonas chlororaphis (Pseudomonas aureofaciens); Pseudomonas fluorescens;	PhzY. (O33414); Phenazine biosynthesis protein phzB. (Q51788); Phenazine biosynthesis protein phzA. (Q51787); PhzB. (O69753); Phenazine biosynthesis protein PhzB. (Q9S508); Probable phenazine biosynthesis protein. (Q9HWH1); Probable phenazine biosynthesis protein. (Q9I2J9); Phenazine biosynthesis protein PhzA. (Q9S509); PhzB. (Q9R9G6); PhzX. (O33413); PhzA. (O69752); PhzA. (Q9R9G7); Probable phenazine biosynthesis protein. (Q7DCE0); Probable phenazine biosynthesis protein. (Q7DC83); EhpA. (Q8GPH5); Putative phenazine antibiotic biosynthesis protein. (Q6D3P1);

PPR~PPR~PPR~PPR~PPR	Anopheles gambiae str. PEST; Arabidopsis thaliana (Mouse-ear cress); Homo sapiens (Human); Neurospora crassa; Oryza sativa (japonica cultivar-group); Plasmodium falciparum (isolate 3D7); Plasmodium yoelii yoelii; R. solanacearum; Solanum demissum (Wild potato);	Fertility restorer. (Q76C20); Unknow protein (Unkonw protein). (Q6ASS1); Hypothetical protein At5g18950/F17K4_200. (Q8GYM2); Hypothetical protein At2g40240 (Hypothetical protein T07M07.12). (Q9S733); Hypothetical protein At4g35850. (Q8VYR5); Hypothetical protein F27K19_210 (Hypothetical proteinAt3g56030/F27K19_210). (Q9LY43); Putative pentatricopeptide (PPR) repeat-containing protein. (Q6ZGL2); Hypothetical protein. (Q8L9P6); Putative pentatricopeptide (PPR) repeat-containing protein. (Q6YUT0); Hypothetical protein. (Q7RYV8); Hypothetical protein. (Q7RJN7); Hypothetical protein. (Q8IM30); Hypothetical protein OJ1134F05.20. (Q8H8A9); Hypothetical protein OJ1754_E06.32. (Q84TA9); Hypothetical protein At5g42450/MDH9_15. (Q8GW82); OSJNBa0086B14.3 protein. (Q7XV52); Putative pentatricopeptide repeat-containing protein. (Q8H5Q4); Hypothetical protein OJ1134F05.14. (Q8H8B5); Membrane-associated salt-inducible protein-like. (Q6K9T5); P0487H02.18 protein. (Q943X5); P0481E12.11 protein. (Q94J37); PUTATIVE PPR REPEATS CONTAINING PROTEIN. (Q8XTC0); Expressed protein. (Q6AVS4); AgCP12383. (Q7PYP1); Putative leaf protein. (Q8LNF7); Putative leaf protein. (Q8LNG1); Hypothetical protein unannotated coding sequence from BAC F20L16(Hypothetical protein At5g18390). (Q94JX6); Putative selenium-binding protein. (Q9SLA5); Hypothetical protein. (Q6F2D0); Putative selenium-binding protein. (Q8S2J3); Hypothetical protein T3M22.4. (Q9C6G2); Gb|AAF34859.1. (Q9LUT4); Hypothetical protein. (Q96EY7);
PRC~PRC~PRC	Methanosarcina acetivorans; M . loti;	Mll3685 protein. (Q98FN8*); Antigen. (Q8TT13);
PTS_EIIA_2~PTS_EIIA_2~PTS-HPr~PEP-utilisers_N~PEP-utilizers~PEP-utilizers_C	P. aeruginosa; P. syringae (pv. tomato);	Phosphoenolpyruvate-protein phosphotransferase,EI/HPr/EIIAcomponents. (Q888R2); Probable phosphotransferase system enzyme I. (Q9HY55);
PepSY_TM~PepSY_TM~PepSY_TM~Flavodoxin_1~NAD_binding_1	P. aeruginosa; P. syringae (pv. tomato);	Iron-uptake factor. (O68591); Iron-uptake factor. (Q87WI1);
Peptidase_C14~TPR_1~TPR_2~TPR_1~TPR_2~TPR_1	M . loti;	Mll5190 protein. (Q98CE0);
Peptidase_C14~TPR_2	M . loti;	Mlr1170 protein. (Q98L57);
Peptidase_C39~RHS_repeat	R. solanacearum;	PUTATIVE RHS-RELATED PROTEIN. (Q8XTG0);
Peptidase_C39~TPR_2	P. aeruginosa;	Hypothetical protein. (Q9I065);
Peptidase_M10~HemolysinCabind~W_rich_C~W_rich_C~W_rich_C	B. japonicum;	Bll6027 protein. (Q89HG4);
Peptidase_M13	Caenorhabditis elegans; Drosophila melanogaster (Fruit fly); X. axonopodis (pv. citri);	Hypothetical protein K02F6.9. (O16636*); Hypothetical protein T20D4.9. (P91466); Hypothetical protein F18A12.4. (O16791); HL07928p. (Q8T0H9); GH14621p. (Q95U11); GH14576p. (Q95SM2); Hypothetical protein C53B7.7. (O45311*); Hypothetical protein ZK1248.1. (Q23427*); GH06227p. (Q961U6); CG3239-PB. (Q8IRR7); CG3239-PA (GH24674p). (Q9W4B8); CG8550-PA. (Q9V6C5*); Hypothetical protein T28A11.20. (P91519); Hypothetical protein C17B7.10. (O45157); Hypothetical protein F19C6.4. (Q09539); Hypothetical protein T28A11.17. (P91515*); Hypothetical protein Y19D10B.6. (Q9N566*); Hypothetical protein C17B7.8. (O45151); Hypothetical protein T20D4.8. (P91467); Hypothetical protein F09D12.2. (O44472); Hypothetical protein F42G10.1. (Q09393); GH11680p. (Q8T0S1); Hypothetical protein F12A10.4. (Q09946); CG4580-PA. (Q9VJK5*); Hypothetical protein F40B5.1 in chromosome X. (Q09319*); Hypothetical protein F40B5.3. (Q95ZU0*); Hypothetical protein F18A12.3. (O16792); Peptidase. (Q8PGA4); CG9780-PB. (Q86BB2); CG9780-PA (GH23891p). (Q9VN01*); Hypothetical protein F39E9.6. (O17115);
Peptidase_M23~HemolysinCabind~HCBP_related~HemolysinCabind~HCBP_related	X. fastidiosa (strain Temecula1 / ATCC 700964);	Hemolysin-type calcium binding protein. (Q879U3);
Peptidase_M23~HemolysinCabind~HCBP_related~HemolysinCabind~HCBP_related~HemolysinCabind~HCBP_related~HemolysinCabind~HCBP_related~HemolysinCabind	R. solanacearum;	PUTATIVE CALCIUM BINDING HEMOLYSIN PROTEIN. (Q8Y2T5);
Peptidase_M23~PG_binding_1	X. axonopodis (pv. citri); X. campestris (pv. campestris);	Lytic enzyme. (Q8PQ62); Lytic enzyme. (Q8PD91);

Peptidase_M28~Autotransporter	E. carotovora subsp. atroseptica SCRI1043; P. aeruginosa;	Hypothetical protein. (Q9I6G3*); PapB protein. (Q9F3Y1); Autotransporter. (Q6D577);
Peptidase_M28~TFR_dimer	Homo sapiens (Human); X. axonopodis (pv. citri);	Peptidase. (Q8PGK5); Prostate-specific membrane antigen-like protein. (Q9HBA9);
PhaC_N	B. japonicum; Paracoccus denitrificans; M . loti; Rhodobacter capsulatus (Rhodopseudomonas capsulata); Rhodobacter sphaeroides (Rhodopseudomonas sphaeroides);	Poly(3-hydroxyalkanoate) synthase. (Q51708); Poly-3-hydroxybutyrate synthase. (Q89M33); Poly-beta-hydroxybutyrate synthase. (Q98LP5); PHA-synthase. (Q8VMB1); Poly-3-hydroxybutyrate synthase. (Q8RLU5); Poly (3-hydroxyalkanoate) synthase. (Q9WX80); Polyhydroxyalkanoate synthase. (O05334); Poly-3-hydroxybutyric acid synthase. (Q9R879); Polyhydroxyalkanoate synthase. (Q53189); Polyhydroxyalkanoic synthase, PHA synthase. (Q9R613);
Phage_Coat_B	Bacteriophage Pf1; P. aeruginosa;	Coat protein B of bacteriophage Pf1). (Q9I5K5); Coat protein B precursor (Major coat protein). (P03621);
Phage_DNA_bind	Bacteriophage IKe; Bacteriophage f1; Bacteriophage fd, Bacteriophage f1, and Bacteriophage M13; Vibrio cholerae; X. campestris (pv. campestris), and Bacteriophage phi-Lf; Xanthomonas phage Cf; X. fastidiosa (strain Temecula1 / ATCC 700964);	Helix-destabilizing protein (Single-stranded DNA binding protein)(GPV). (P03670); RstB1. (Q9ANX6); Helix-destabilizing protein (Single-stranded DNA-binding protein) (SBP). (Q07481); Helix-destabilizing protein (Single-stranded DNA binding protein) (GPV). (P03669); V protein. (O80282); Bacteriophage f1 genes v, vii and viii. (Q38210); Phage-related protein. (Q87CW6);
Phage_T7_tail	A. tumefaciens ; Bacteriophage T3; Bacteriophage T7; Bacteriophage phiYeO3-12; Pseudomonas phage gh-1; Yersinia pestis phage phiA1122;	Tail fiber protein. (Q9T0Z9); Tail fiber protein. (P03748); Tail fiber protein. (P10308); Tail fiber protein. (Q8LTU2); Tail fiber protein. (Q8LTU3); Tail fiber protein. (Q8LTU4); Tail fiber protein. (Q8LTU5); Tail fiber protein. (Q8LTU6); Tail fiber protein. (Q8LTU7); Tail fiber protein. (Q8LTU8); Tail fiber protein. (Q8LTU9); Tail fiber protein. (Q8LTV0); Tail fiber protein. (Q8LTW7); Tail fiber protein. (Q8LTW8); Tail fiber protein. (Q8LTW9); Tail fiber protein. (Q8LTX0); Tail fiber protein. (Q8LTX1); Tail fiber protein. (Q8LTX2); Tail fiber protein. (Q8LTX3); Tail fiber protein. (Q8LTX4); Tail fiber protein. (Q8LTX5); Tail fiber protein. (Q8LTX6); Tail fiber protein. (Q8LTX7); Tail fiber protein. (Q8LTX8); Tail fiber protein. (Q8LTX9); Tail fiber protein. (Q8LTY0); Tail fiber protein. (Q8LTY1); Tail fiber protein. (Q8LTY2); Tail fiber protein. (Q8W5T8); Gene 17. (Q6WYH2); Gene 17. (Q6WYC3); Gene 17. (Q6WY69); Gene 17. (Q6WY17); Tail fiber protein. (Q858J8); Tail fiber protein. (Q859E1); AGR_C_2190p. (Q7CZT0); Tail fiber protein. (Q8UG54);
Phage_holin_2	Bacteriophage 186; Bacteriophage L-413C; Bacteriophage P2; Bacteriophage PSP3; Bacteriophage WPhi; E. carotovora subsp. atroseptica SCRI1043;	Holin. (P51773*); Orf24; P2 Y homolog; holin. (O80308*); Phage lysis protein Y, holin. (Q6D3X6); GpY. (Q773P5); Gp9. (Q6K1I2); GpY. (Q858W2);
Phage_integr_N~Peptidase_C48	B. japonicum;	Blr1693 protein. (Q89TT5);
Phage_integr_N~Phage_integr_N~Phage_integrase	Alcaligenes eutrophus (Ralstonia eutropha); Photorhabdus luminescens (Xenorhabdus luminescens); Plasmid pAE1; M . loti; S. meliloti;	Int. (Q8GDM2); Lambda integrase family of site specific recombinase; ORF2. (Q52212); Putative integrase/recombinase. (Q7WS65); Putative integrase/recombinase. (Q925Y2); PUTATIVE INTEGRASE/RECOMBINASE PROTEIN. (Q8KJE1); Putative integrase/recombinase. (Q7WXJ4); Orf2/integrase/recombinase fusion protein. (Q7WXS5); Putative integrase/recombinase. (Q7WXS2);

Phenol_Hydrox	Acinetobacter calcoaceticus; Acinetobacter sp; Alcaligenes eutrophus (Ralstonia eutropha); B. japonicum; Burkholderia cepacia (Pseudomonas cepacia); Burkholderia kururiensis; Burkholderia pickettii (Pseudomonas pickettii); Comamonas testosteroni (Pseudomonas testosteroni); Gordonia sp. TY-5; Methylococcus capsulatus; Methylocystis sp. M; Methylocystis sp. WI14; Methylomonas sp. KSPIII; Methylomonas sp. KSWIII; Methylosinus trichosporium; Mycobacterium rhodesiae; Plasmodium yoelii yoelii; Pseudomonas butanovora; Pseudomonas mendocina; Pseudomonas putida; Pseudomonas sp; Pseudomonas sp. (strain CF600); Pseudomonas sp. KL28; Pseudomonas stutzeri (Pseudomonas perfectomarina); Pseudonocardia sp. K1; Ralstonia sp. E2; Ralstonia sp. KN1; Rhodococcus corallinus; Rhodococcus sp. AD45; Sulfolobus solfataricus; Xanthobacter sp. (strain Py2);	Methane monooxygenase component A beta chain (EC 1.14.13.25) (Methanehydroxylase). (P27354); Soluble methane monooxygenase hydroxylase component (MMOH) betasubunit. (Q7DJP6); Methane monooxygenase component a beta chain. (Q7R762); MmoY. (Q9RAM2); Methane monooxygenase component A beta chain (EC 1.14.13.25) (Methanehydroxylase). (P18798); Soluble methane monooxygenase hydroxylase component (MMOH) betasubunit. (Q9R2S6); Soluble methane monooxygenase protein A beta subunit. (O06117); Propane monooxygenase hydroxylase small subunit. (Q768T3); Putative alkene monooxygenase beta subunit. (Q6XBI8); Butane monooxygenase hydroxylase BMOH beta subunit. (Q8KQE9); Blr3679 protein. (Q89P04); Toluene-4-monooxygenase system protein A. amino end (TmoA) (EC1.14.13.-). (Q97YT1); Epoxidase subunit. (Q53025); Toluene-4-monooxygenase system protein E. (TmoE) (EC 1.14.13.-). (Q97YS6); Propane monooxygenase hydroxylase large subunit. (Q768T5); Alpha-subunit of multicomponent tetrahydrofuran monooxygenase. (Q9F3V6); Blr3677 protein. (Q89P06); DMS oxygenase component. (O32429); Phenolhydroxylase component. (O43979); Phenol hydroxylase component. (Q9RAF8); Soluble methane monooxygenase hydroxylase component (MMOH) alphasubunit. (Q9R3H0); Soluble methane monooxygenase protein A alpha subunit. (O06116); Toluene-3-monooxygenase oxygenase subunit 2. (O07072); Phenol hydroxylase P1 protein (EC 1.14.13.7) (Phenol 2-monooxygenaseP1 component). (P19730); Phenol hydroxylase. (Q52171); Subunit of phenolhydroxylase. (Q52162); Butane monooxygenase hydroxylase BMOH alpha subunit. (Q8KQF0); Soluble methane monooxygenase hydroxylase component (MMOH) alphasubunit. (Q7DJP7); Methane monooxygenase component A alpha chain (EC 1.14.13.25) (Methanehydroxylase). (P22869); Methane monooxygenase component A alpha chain (EC 1.14.13.25) (Methanehydroxylase). (P27353); Phenol hydroxylase component. (O84959); Epoxidase subunit. (Q53027); Putative alkene monooxygenase alpha subunit. (Q6XBI6); LapL. (Q7WYF3); Phenol hydroxylase component. (Q9ZNP6); Oxygenase beta subunit. (Q9ZET3); CrpB. (O30592); Phenol hydroxylase subunit PhkB. (Q8VUU1); Putative isoprene monooxygenase beta subunit. (Q9RBN8); Phenol hydroxylase subunit. (Q9S148); Tbc1B monooxygenase. (Q9EZP4); Butylphenol hydroxylase component A2. (Q842G5); Toluene-4-monooxygenase system protein E (EC 1.14.13.-). (Q00460); TomA1. (Q9ANX4); Tbc2E monooxygenase. (Q9EZN7); Putative hydroxylase component. (O69182); Phenol hydroxylase component phL. (Q84AQ4); Toluene-3-monooxygenase oxygenase subunit (Beta hydroxylasesubunit). (Q51943); Beta hydroxylase. (Q6Q8Q3); Toluene, o-xylene monooxygenase oxygenase subunit (Toluene o-xylenemonooxygenase component). (O87802); TbmB protein. (Q52570); Beta-subunit of multicomponent tetrahydrofuran monooxygenase. (Q9F3V3); CrpD. (O30590);
Pilin	Neisseria gonorrhoeae; X. axonopodis (pv. citri); X. campestris (pv. campestris);	PilE1 L-pilin (MS11-D3a). (Q51022); PiLE1 L-pilin (MS11-D1). (Q51021); Fimbrial protein. (Q8PG16); Fimbrial protein. (Q8P4F5*);
Pkinase~TPR_4~TPR_2	X. axonopodis (pv. citri);	Serine/threonine kinase. (Q8PF58);
Poly_export~OEP	M . loti;	Exopolysaccharide production protein; ExoF. (Q98C90);
Polysacc_deac_1~Peptidase_C14~TPR_2	X. axonopodis (pv. citri); X. campestris (pv. campestris); X. fastidiosa (strain Temecula1 / ATCC 700964); X. fastidiosa;	Polysaccharide deacetylase. (Q8P339); Polysaccharide deacetylase. (Q8PEH8*); NodB-like protein. (Q8KQT5*); Hypothetical protein. (Q879S4); Hypothetical protein. (Q9P9U2*);
Pyr_redox~Fer4~Fer4	B. japonicum; Desulfotalea psychrophila LSv54;	Blr6742 protein. (Q89FF8); Probable glutamate synthase, small chain. (Q6APV2);
Pyr_redox~Pyr_redox~Fer2_BFD~NIR_SIR_ferr~NIR_SIR	E. carotovora subsp. atroseptica SCRI1043;	Nitrite reductase (EC 1.7.1.4). (Q6D2V3);
RHS_repeat~F5_F8_type_C	R. solanacearum;	PUTATIVE RHS-RELATED PROTEIN. (Q8XTM1);
RHS_repeat~F5_F8_type_C~F5_F8_type_C	R. solanacearum;	PUTATIVE RHS-RELATED TRANSMEMBRANE PROTEIN. (Q8XSJ7*);
RNA_pol_Rpb6~RNA_pol_Rpb6	M . loti;	DNA-directed RNA polymerase omega chain (EC 2.7.7.6) (RNAP omegasubunit) (Transcriptase omega chain) (RNA polymerase omega subunit). (Q985B3);

RVP	Avian myeloblastosis associated virus (MAV); Bovine leukemia virus (Japanese isolate BLV-1) (BLV); Caenorhabditis elegans; Homo sapiens (Human); Human T-cell leukemia virus type I (Caribbean isolate) (HTLV-I); Human T-cell leukemia virus type I (strain ATK) (HTLV-I); Human T-cell leukemia virus type II (HTLV-II); Human T-lymphotropic virus 1; Human immunodeficiency virus 1; Methanosarcina acetivorans; P. syringae (pv. tomato); Simian T-lymphotropic virus 1;	Proteinase p15 (EC 3.4.23.-). (P26315); Protease (EC 3.4.23.-). (P10270); Protease (EC 3.4.23.-). (P03353); Protease (EC 3.4.23.-). (P14074); Protease (EC 3.4.23.-). (P10274); Hypothetical protein. (Q87VH5); Hypothetical protein FLJ16592. (Q6ZMY2); Hypothetical protein K02E2.6. (Q9U3C4); Hypothetical protein FLJ45494. (Q6ZSI7); Predicted protein. (Q8TM77); HXB2=VIRAL protease. (Q9PXK6); Aspartic protease. (Q9PXQ0); HXB2=VIRAL protease. (Q9PXK7); Protease. (Q9WS59); HXB2=VIRAL protease. (Q9PXK5); HXB2=VIRAL protease. (Q9PXK4); HXB2=VIRAL protease. (Q9PXK3); Protease. (Q9WS54); Protease. (Q82323);
Radical_SAM~Gln-synt_N	M . loti;	L-lysine 2,3-aminomutase. (Q98AJ8);
Reg_prop~Y_Y_Y~HisKA_3~HATPase_c	X. axonopodis (pv. citri); X. campestris (pv. campestris);	Two-component system sensor protein. (Q8PKK4*); Two-component system sensor protein. (Q8P8R0*);
Reg_prop~Y_Y_Y~HisKA~HATPase_c~Response_reg	Myxococcus xanthus; X. axonopodis (pv. citri); X. campestris (pv. campestris); X. fastidiosa (strain Temecula1 / ATCC 700964); X. fastidiosa;	Histidine kinase-response regulator hybrid protein. (Q8PI70); Histidine kinase-response regulator hybrid protein. (Q8PI69*); Histidine kinase/response regulator hybrid protein. (Q8P6W9*); Hybrid sensor. (Q9AJP5); Histidine kinase/response regulator hybrid protein. (Q8P6W7*); Histidine kinase-response regulator hybrid protein. (Q8PI67*); Histidine kinase/response regulator hybrid protein. (Q8P6W8*); Histidine kinase/response regulator hybrid protein. (Q87DV4); Hypothetical protein. (Q9PDP9*);
RepA_C	Azospirillum brasilense; Bifidobacterium asteroides; Bifidobacterium longum biovar Longum; Bifidobacterium longum; Chlorobium limicola; Corynebacterium glutamicum (Brevibacterium flavum); Corynebacterium jeikeium; Corynebacterium striatum; Escherichia coli; Plasmid pBFp1; Plasmid pIPO2T; Plasmid pSB102; Plasmid pSa; P. aeruginosa; R. solanacearum; Rhodopseudomonas palustris; Rhodothermus marinus (Rhodothermus obamensis); Salmonella enterica subsp. enterica serovar Typhimurium; Sphingomonas aromaticivorans; X. axonopodis (pv. citri); X. campestris (pv. vesicatoria); Xanthomonas maltophilia (Pseudomonas maltophilia) (Stenotrophomonas maltophilia); uncultured bacterium;	PROBABLE REPA REPLICASE PROTEIN. (Q8XTV3); RepA. (Q9FB52); RepW. (Q93MJ1); RepA. (Q84GE3); RepA protein. (Q52874); Replication protein RepA. (Q9RBJ8); RepA. (Q6VRU0); Replicase. (Q8GN72); RepA replication protein. (O85860); RepB. (Q83YK3); RepA replicase. (P96957); RepA protein. (Q6I6A6); Replication protein A. (Q8PRH6); Putative replication protein. (Q7WZM5); RepA. (Q04559); Replication protein. (Q9ADU6); Replication protein. (Q9RHL0); RepA protein. (Q91UU1); Replication initiation protein. (Q8VVB0); RepA protein. (Q91UU9); RepA homolog. (P94651); Plasmid replication protein. (Q6QW75); Plasmid replication protein. (Q9L449); RepA. (Q6X3G6); Replication protein. (Q8PJT9);
RepB	A. tumefaciens ; P. syringae (pv. tomato); M . loti; S. meliloti;	Msr9757 protein. (Q98P91); Mll8115 protein. (Q983Y2); Hypothetical protein. (Q88BH6); Hypothetical protein Atu5040. (Q8UKR0); AGR_pAT_52p. (Q7D423); Hypothetical protein. (Q92XS2); Hypothetical protein. (Q930E6); Hypothetical protein. (Q930E5);
Response_reg~Guanylate_cyc	B. japonicum; Stigmatella aurantiaca;	Adenylate cyclase 2 (EC 4.6.1.1) (ATP pyrophosphate-lyase 2) (Adenylylcyclase 2) (AC2). (P40138); Two-component response regulator. (Q89RA8);
Response_reg~HWE_HK	A. tumefaciens ; Rhodospirillum centenum (Rhodocista centenaria);	CstS3. (Q7X4X1); Two component sensor kinase. (Q8UDX8); AGR_C_3616p. (Q7CY47);
Response_reg~PAC~HWE_HK	B. japonicum;	Two-component response regulator. (Q89S14);
Response_reg~PAC~HisKA~HATPase_c~Response_reg	M . loti;	Probable sensor/response regulator hybrid protein. (Q98II8);
Response_reg~PAS~HisKA~HATPase_c~Response_reg	X. axonopodis (pv. citri); X. campestris (pv. campestris);	Histidine kinase-response regulator hybrid protein. (Q8PN01); Histidine kinase/response regulator hybrid protein. (Q8PBE6);
Response_reg~Sigma54_activat~HisKA~HATPase_c	X. axonopodis (pv. citri);	Histidine kinase-response regulator hybrid protein. (Q8PGH3);
RgpF~Hexapep	A. tumefaciens ;	Hypothetical protein Atu4606. (Q8U749); AGR_L_543p. (Q7CVD9);

RgpF~RgpF	M . loti; Rhizobium sp. (strain NGR234); X. axonopodis (pv. citri); X. campestris (pv. campestris);	Mll4799 protein. (Q98D97); Hypothetical protein XAC3576. (Q8PGP0); Hypothetical protein wxcX. (O34262); Hypothetical 45.0 kDa protein y4gN. (P55470);
Rhamnogal_lyase	Arabidopsis thaliana (Mouse-ear cress); E. carotovora subsp. atroseptica SCRI1043; Erwinia chrysanthemi; Oryza sativa (japonica cultivar-group);	F21M12.30 protein. (O04512); Hypothetical protein At4g37950. (Q84W85); Hypothetical protein At1g09910/F21M12_30. (Q8GX61); At2g22620. (Q6NKW8); Hypothetical protein At2g22620. (Q9ZQ51*); LG127/30 like gene. (Q9SZK3*); F21M12.28 protein. (O04511); Putative MYST1. (Q6Z3F5); LG27/30-like gene. (Q9STV1); Rhamnogalacturonate lyase. (Q6D915); Rhamnogalacturonate lyase precursor (EC 4.2.2.-)(Rhamnogalacturonase). (Q8RJP2*); F21M12.27 protein. (O04510); Hypothetical protein F20D10.70 (Hypothetical protein AT4g37950). (Q9SZJ7*); T23K8.12 protein. (Q9S9K1); AT4g24430/T22A6_260. (Q93Z90);
Rhodanese~Cys_Met_Meta_PP	P. syringae (pv. tomato); Zymomonas mobilis;	Cystathionine-gamma-lyase. (Q9RNJ8); Rhodanese domain protein/cystathionine beta-lyase. (Q882J5);
Rhodanese~Rhodanese~PS_Dcarbxylase	P. syringae (pv. tomato);	Rhodanese domain protein/phosphatidylserine decarboxylase. (Q87VI1);
Ribosomal_60s~Ribosomal_L12	R. solanacearum; uncultured marine gamma proteobacterium EBAC20E09;	Predicted ribosomal protein L7/L12. (Q6Q8Z5); 50S ribosomal protein L7/L12. (Q8XUZ7);
Rieske~FAD_binding_6~NAD_binding_1~Fer2	S. meliloti;	Putative oxidoreductase. (Q92XT7);
Rieske~NAD_binding_1~Fer2	S. meliloti;	Putative oxidoreductase/oxygenase. (Q92YP7);
RolB_RolC	Agrobacterium rhizogenes; A. tumefaciens (strain Ach5), and A. tumefaciens (strain 15955); A. tumefaciens (strain Ach5); A. tumefaciens ; A. tumefaciens; Agrobacterium vitis (Rhizobium vitis); Nicotiana glauca (Glaucous tobacco) (Tree tobacco); Nicotiana tabacum (Common tobacco); Plasmid Ti;	Protein-tyrosine phosphatase rolB (EC 3.1.3.48) (ROL B protein). (P49409); Protein-tyrosine phosphatase rolB (EC 3.1.3.48) (ROL B protein). (P20402); ORF11 (Hypotethical protein, homologous to ORF11 of pRiA4). (Q44182); ORF11. (Q44193); Riorf15 protein. (Q9F5H3); ROL B(TR) protein. (P15397); Hypothetical protein. (Q9ADZ8); Tiorf179 protein. (Q9R698); E protein. (Q44409); E protein. (Q7CNY2); AGR_pTi_45p. (Q7D2R8); Hypothetical protein torf11. (Q9R475); Protein-tyrosine phosphatase rolB (EC 3.1.3.48) (ROL B protein). (P09178); Hypothetical protein torf9. (Q9WWE5); AGR_pTi_42p. (Q7D2S0); AGR_pTi_43p. (Q7D2R9); C' protein. (Q7CNY3); D protein. (Q8U6A7); D protein. (Q9R716); C' protein. (Q9R715); Tiorf178 protein. (Q9R699); A6b protein. (Q57530); Hypothetical protein torf10. (Q9WWE4); Tiorf177 protein. (Q9R6A0); Hypothetical protein. (Q9ADZ9); AGR_pTi_bx99p. (Q7D2R1); 6B protein. (P25019); Protein 6b. (Q8U6A2); 6B. (P97020); 6B protein. (P04031); Tml. (Q7BLS3); 6b protein. (Q52605); Tiorf186 protein. (Q7DKB7); 6b protein. (P94208); Protein 6b. (Q9R470); 6b protein. (Q44322); 6B protein. (Q04551); Ti plasmid pTi15955 T-DNA region (Gene 3' protein). (Q44400); 3' protein. (Q44523); 696 protein. (Q44442); AGR_pTi_bx89p. (Q7D2S2); 5 protein. (P94209); 6b protein. (Q44522); B protein. (Q9R714); 5 protein. (P94204); 5 protein. (Q7CNY4); 5 protein. (Q9X5L0); T-DNA oncoprotein. (O88163); Hypothetical protein torf7 (Tiorf173 protein). (Q9WWE6); Hypothetical protein torf14. (Q9WWE3); 5 protein. (Q9WWB4); AGR_pTi_bx83p. (Q7D2R5); Tiorf181 protein. (Q9R696); ORF13. (Q51966); RolD protein (Riorf17 protein). (Q44202); Hypotethical protein, homologous to ORF13 of pRiA4. (Q9R721); ORF13. (Q44195); Ngorf13R protein. (Q8S955); NgORF13 protein. (Q40393); Torf13-1 protein. (Q9ZS37); ORF14 (Riorf19 protein). (Q51968); Hypotethical protein, homologous to ORF14 of pRiA4. (Q9R719); NgORF14 protein. (Q40394); Ngorf14R protein. (Q8S953); ORF14. (Q44197); TrolC protein. (Q43592); ORF12. (Q44194); ORF12 (Hypotethical protein, homologous to ORF12 of pRiA4). (Q44183); Cytokinin-beta-glucosidase (EC 3.2.1.-) (ROL C protein). (P49408); Cytokinin-beta-glucosidase (EC 3.2.1.-) (ROL C protein). (P07051); Cytokinin-beta-glucosidase (EC 3.2.1.-) (ROL C protein). (P20403); Hypothetical protein 6. (P04030); Ons. (Q7BLS4); 6a protein. (Q52604); Hypothetical protein 1 (Gene 5 protein). (P04028); Tiorf185 protein (Protein 6a). (Q9R430); Protein 6a. (Q7CNX9); AGR_pTi_51p. (Q7D2R2); Gene 5 protein. (Q7CNY1);

RolB_RolC~Amino_oxidase	Agrobacterium rhizogenes; A. tumefaciens (strain Ach5), and A. tumefaciens; A. tumefaciens ; A. tumefaciens; Agrobacterium vitis (Rhizobium vitis);	Riorf13 protein. (Q9F5H4); Hypotethical protein, homologous to ORF8 of pRiA4. (Q9R722); Tryptophan 2-monooxygenase. (Q9ZHH0); Tryptophan 2-monooxygenase (EC 1.13.12.3). (P25017); Tryptophan 2-monooxygenase (EC 1.13.12.3). (P04029); Tryptophan 2-monooxygenase. (Q8U6A3); Tryptophan monooxygenase. (Q9WWA1); Ti plasmid pTi15955 T-DNA region (IaaM). (Q44388); Tryptophan monooxygenase. (Q9R717); Tiorf183 protein. (Q9R694); AGR_pTi_49p. (Q7D2R3); Tryptophan 2-monooxygenase (EC 1.13.12.3). (Q09109); Tryptophan 2-monooxygenase. (Q9R472); Tryptophan 2-monooxygenase (EC 1.13.12.3). (Q04564);
SAM_1~Guanylate_cyc	S. meliloti;	Putative adenylate cyclase. (Q92Y97);
SAM_1~Guanylate_cyc~TPR_2	B. japonicum; S. meliloti;	Putative adenylate cyclase. (Q92YL0); Bll6707 protein. (Q89FJ3);
SAM_1~Guanylate_cyc~TPR_2~TPR_4	B. japonicum;	Blr2238 protein. (Q89T12);
SBP56	Arabidopsis thaliana (Mouse-ear cress); Brachydanio rerio (Zebrafish) (Danio rerio); B. japonicum; Caenorhabditis elegans; Drosophila melanogaster (Fruit fly); Homo sapiens (Human); Lotus japonicus; Medicago sativa (Alfalfa); Mus musculus (Mouse); Oryza sativa (Rice); Oryza sativa (japonica cultivar-group); Pyrobaculum aerophilum; Rattus norvegicus (Rat); Sulfolobus solfataricus; Sulfolobus tokodaii; Thellungiella halophila (Salt cress); Xenopus laevis (African clawed frog);	CG7966-PA. (Q9VFZ4); Putative selenium binding protein. (Q8RZW7); Putative selenium-binding protein. (O23264); Selenium-binding protein-like protein. (Q8L7F3); Selenium-binding protein. (Q93WN0); AT4g14040/dl3061c. (Q9ASS5); Hypothetical protein. (Q6DCH7); Selenium binding protein. (Q93VA1); Selenium binding protein. (Q93WS1); Selenium-binding protein. (Q9LK38); Selenium binding protein 1 (SELENBP1 protein). (Q96GX7); Selenium-binding protein 2 (56 kDa acetaminophen-binding protein)(AP56). (Q63836); Selenium-binding protein 1 (56 kDa selenium-binding protein) (SP56). (P17563); Selenium-binding protein 1. (Q13228); Selenium binding protein 2. (Q8VIF7); Selenium binding protein 2. (Q8R1T6); Selenium binding protein 1. (Q91X87); GH14316p. (Q7K537); Hypothetical protein Y37A1B.5. (Q9XXF9); Bll7952 protein. (Q89C46); Hypothetical protein (Selenium-binding protein like). (O23265); Putative selenium binding protein. (Q9AVA6); Selenium binding protein, probable. (Q8ZUL2); 462aa long hypothetical selenium-binding protein. (Q976Y0); Selenium-binding protein. (Q97UY0); Hypothetical protein zgc:65844. (Q6PHD9); Putative selenium-binding protein. (Q21950); Putative selenium-binding protein. (Q8S2S4); Hypothetical protein FLJ13813. (Q9H8A8);
SBP_bac_3~PAS	P. syringae (pv. tomato);	Extracellular solute-binding protein/sensory box protein. (Q883M7);
SMP-30~Isochorismatase	B. japonicum;	Bll3369 protein. (Q89PW1);
ST7	Bos taurus (Bovine); Caenorhabditis elegans; Drosophila melanogaster (Fruit fly); Fugu rubripes (Japanese pufferfish) (Takifugu rubripes); Gallus gallus (Chicken); Homo sapiens (Human); Mus musculus (Mouse); M . loti; Xenopus laevis (African clawed frog);	ST7 protein form1 splice variant a. (Q99M96*); Lrp12 protein. (Q8R3T3*); FAM4A1 splice variant b (Suppression of tumorigenicity 7, isoformb). (Q9NRC1*); ST7 protein form3 splice variant a. (Q99M92); FAM4A1 splice variant a. (Q9NRC2*); Hypothetical protein. (Q6DDN4); ST7 protein form1 splice variant b. (Q99M95*); CG3634-PA (RE50559p). (Q9VPB1); ST7L isoform 1 (Suppression of tumorigenicity 7-like, isoform 1). (Q8TDW4); ST7 protein. (Q8NEJ8*); ST7 protein form4 splice variant a. (Q99M90); Suppression of tumorigenicity 7. (Q6Q9W3); Fam4a2 splice variant a. (Q9JID8); Hypothetical protein F11A10.5. (Q19337*); ST7 form 2 splice variant a. (Q9BWX4); ST7 protein form2 splice variant a. (Q99M94); Hypothetical protein HELG. (Q90YH8); ST7 protein form3 splice variant b. (Q99M91); Tumorsuppressor St7-like product (Suppression of tumorigenicity 7-like). (Q8K4P7); ST7L isoform 2. (Q8TDW3); ST7 protein. (Q98SK4); ST7L isoform 4. (Q8TDW1); Hypothetical protein DKFZp686N2430. (Q7Z3C2); ST7 protein form4 splice variant b. (Q99M89); ST7 protein form2 splice variant b. (Q99M93); Suppression of tumorigenicity 7 isoform. (Q6Q9W2); Fam4a2 splice variant b. (Q9JID7); ST7 form 2 splice variant b. (Q9BWX3); HELG protein. (Q9NXZ7); ST7L isoform 3. (Q8TDW2); Hypothetical protein FLJ35893. (Q8NA32); ST7 protein. (Q8UW40); Hypothetical protein FLJ40518. (Q8N7P8); ST7 protein. (Q9BDI1); Hypothetical protein FLJ20284. (Q9NXF3); Mlr6324 protein. (Q989Q8);

START	Anguilla japonica (Japanese eel); Arabidopsis thaliana (Mouse-ear cress); Bombyx mori (Silk moth); Bos taurus (Bovine); Brachydanio rerio (Zebrafish) (Danio rerio); Branchiostoma belcheri (Amphoxius); Branchiostoma belcheri tsingtaunese; Caenorhabditis elegans; Drosophila melanogaster (Fruit fly); Equus caballus (Horse); Gadus morhua (Atlantic cod); Gallus gallus (Chicken); Giardia lamblia ATCC 50803; Homo sapiens (Human); Mesocricetus auratus (Golden hamster); Mus musculus (Mouse); Oncorhynchus mykiss (Rainbow trout) (Salmo gairdneri); Oryza sativa (japonica cultivar-group); Ovis aries (Sheep); Poephila guttata (Zebra finch) (Taeniopygia guttata); Potamotrygon hystrix (porcupine river stingray); Potamotrygon motoro (South American freshwater stingray); P. aeruginosa; Rattus norvegicus (Rat); Salvelinus fontinalis (Brook trout) (Brook char); Sus scrofa (Pig); Xenopus laevis (African clawed frog); Xenopus tropicalis (Western clawed frog) (Silurana tropicalis);	Carotenoid-binding protein. (Q8MYA9); Hypothetical protein At3g03260/T17B22_5. (Q8GY47); Arabidopsis thaliana homeodomain protein AHDP (SP:P93041). (O65281); Zgc:56270 protein. (Q7ZV19); Zgc:77753 protein. (Q6P3I3); StAR-related lipid transfer protein 6 (StARD6) (START domain-containing protein 6). (P59095); Hypothetical protein. (Q6AYN5); MGC80895 protein. (Q6GNT3); Steroidogenic acute regulatory protein short form (Steroidogenic acuteregulatory protein long form). (Q6Q7W2); Steroidogenic acute regulatory protein. (Q6Q7W3); Steroidogenic acute regulatory protein. (Q7T3M6); Steroidogenic acute regulatory protein, mitochondrial precursor (StAR)(StARD1). (P79245); Steroidogenic acute regulatory protein, mitochondrial precursor (StAR)(StARD1). (Q9DE06); Steroidogenic acute regulatory protein. (Q6RCE2); Steroidogenic acute regulatory protein, mitochondrial precursor (StAR) (StARD1). (P97826); Steroidogenic acute regulatory protein, mitochondrial precursor (StAR)(StARD1). (Q28996); Steroidogenic acute regulatory protein, mitochondrial precursor (StAR)(StARD1). (Q9DEB4); Steroidogenic acute regulatory protein, mitochondrial precursor (StAR)(StARD1) (Luteinizing hormone-induced protein). (P51557); Steroidogenic acute regulatory protein, mitochondrial precursor (StAR) (StARD1). (P70114); Steroidogenic acute regulatory protein, mitochondrial precursor (StAR)(StARD1). (P49675); Steroidogenic acute regulatory protein, mitochondrial precursor (StAR)(StARD1). (O46689); Steroidogenic acute regulatory protein, mitochondrial precursor (StAR)(StARD1). (Q9DG09); Steroidogenic acute regulatory protein, mitochondrial precursor (StAR)(StARD1). (Q9DG10); Steroidogenic acute regulatory protein, mitochondrial precursor (StAR)(StARD1). (Q28918); Steroidogenic acute regulatory protein. (Q7ZT79); MLN 64 protein (StAR-related lipid transfer protein 3) (StARD3) (STARTdomain-containing protein 3) (ES 64 protein). (Q61542); MLN 64 protein (StAR-related lipid transfer protein 3) (StARD3) (STARTdomain-containing protein 3) (CAB1 protein). (Q14849); Hypothetical protein. (Q6DFR7); START domain containing 3. (Q6PH03); MGC68989 protein. (Q6PF40); Phosphatidylcholine transfer protein. (Q6WN56); Steroidogenic acute regulatory protein. (Q7T3M5); StAR-related lipid transfer protein 7 (StARD7) (START domain-containing protein 7). (Q8R1R3); StAR-related lipid transfer protein 7 (StARD7) (START domain-containing protein 7) (GTT1 protein). (Q9NQZ5); Mus musculus adult male tongue cDNA, RIKEN full-length enrichedlibrary, clone:2310058G22 product:STAR-RELATED PROTEIN 1-4E, fullinsert sequence. (Q9CQY0); Hypothetical protein F52F12.7 in chromosome I. (O17883); PCTP-like protein (PCTP-L) (StAR-related lipid transfer protein 10)(StARD10) (START domain-containing protein 10) (CGI-52) (Serologicallydefined colon cancer antigen 28) (Antigen NY-CO-28). (Q9Y365); Phosphatidylcholine transfer protein (PC-TP) (StAR-related lipidtransfer protein 2) (StARD2) (START domain-containing protein 2). (P53809); StAR-related lipid transfer protein 5 (StARD5) (START domain-containing protein 5). (Q9NSY2); Phosphatidylcholine transfer protein (PC-TP) (StAR-related lipidtransfer protein 2) (StARD2) (START domain-containing protein 2). (P02720); Phosphatidylcholine transfer protein (PC-TP) (StAR-related lipidtransfer protein 2) (StARD2) (START domain-containing protein 2). (Q9UKL6); Phosphatidylcholine transfer protein (PC-TP) (StAR-related lipidtransfer protein 2) (StARD2) (START domain-containing protein 2). (P53808); Hypothetical protein zgc:77147. (Q6P0C0); MGC81120 protein. (Q6NU97); StAR-related lipid transfer protein 5 (StARD5) (START domain-containing protein 5). (Q9EPQ7); Hypothetical serologically defined colon cancer antigen 28. (Q86LQ0); PCTP-like protein (PCTP-L) (StAR-related lipid transfer protein 10)(StARD10) (START domain-containing protein 10) (Serologically definedcolon cancer antigen 28 homolog). (Q9JMD3); Hypothetical protein T28D6.7. (O18155); StAR-related lipid transfer protein 6 (StARD6) (START domain-containing protein 6). (P59096); Mus musculus adult male testis cDNA, RIKEN full-length enrichedlibrary, clone:4933429L05 product:RIKEN cDNA 4833424I06 (START domaincontaining protein 6). (Q9CPT8); Mus musculus 3 days neonate thymus cDNA, RIKEN full-length enrichedlibrary, clone:A630084D14 product:CDNA FLJ30004 FIS, CLONE3NB691000116, WEAKLY SIMILAR TO STEROIDOGENIC ACUTE REGULATORY PROTEINhomolog (Mus musculus 7 days neonate cerebellum cDNA, RIKEN full-length enriched library, clone:A730016F08 product:CDNA FLJ30004 FIS,CLONE 3NB691000116, WEAKLY SIMILAR TO

SecIII_HopPtoS	P. syringae (pv. tomato);	Type III effector HopPtoS3. (Q87WF6); Hypothetical protein. (Q87WF9); Type III effector HopPtoS1. (Q88BP8);
Sigma54_DBD	S. meliloti;	Hypothetical protein. (Q92ZL5);
Sigma54_activat~HTH_8~TPR_4	P. syringae (pv. tomato);	Sigma-54 dependent transcriptional regulator. (Q883U1);
Sigma54_activat~HTH_AraC~HTH_AraC	X. axonopodis (pv. citri);	Transcriptional regulator. (Q8PI72);
Sigma70_r1_1	B. japonicum;	Bsr8137 protein. (Q89BL1);
Succ_DH_flav_C	Coxiella burnetii; Mycobacterium leprae; Pyrobaculum aerophilum; M . loti; Rhodococcus ruber;	Succinate dehydrogenase. (Q8KLT8); Adenylylsulfate reductase alpha subunit part 2, authenticframeshift. (Q8ZUX3); Lepb1170_C2_203. (Q49623); Succinate dehydrogenase flavoprotein subunit homolog. (Q9R493); Msl6271 protein. (Q989V0);
Sulfate_transp~DUF1458	B. japonicum;	Bll5352 protein. (Q89JD1);
Sulfate_transp~STAS~cNMP_binding	B. japonicum; Kluyveromyces lactis NRRL Y-1140; Neurospora crassa; Saccharomyces cerevisiae (Baker's yeast); Schizosaccharomyces pombe (Fission yeast); Yarrowia lipolytica CLIB99;	Hypothetical 117.0 kDa protein in ASN2-PHB1 intergenic region. (P53273); Similar to sp|P53273 Saccharomyces cerevisiae YGR125w. (Q6CPI0); Hypothetical protein C24H6.11c in chromosome I. (Q09764); Hypothetical protein. (Q7SDH8); Similar to ca|CA0686|IPF162 Candida albicans. (Q6C0P1); Bll0811 protein. (Q89W82);
TIG~He_PIG~He_PIG~He_PIG~He_PIG~He_PIG~He_PIG~He_PIG~He_PIG~Autotransporter	R. solanacearum;	PUTATIVE HEMAGGLUTININ-RELATED PROTEIN. (Q8Y366);
TM_helix~TM_helix	P. syringae (pv. tomato); Pyrobaculum aerophilum; Sulfolobus solfataricus; Sulfolobus tokodaii;	Hypothetical protein ST2163. (Q96YK9*); Membrane protein, putative. (Q883Q3); Conserved within P. aerophilum. (Q8ZWH2*); Hypothetical protein. (Q97WM7);
TPR_1~TPR_2~Peptidase_C14	B. japonicum;	Blr5947 protein. (Q89HP3);
TPR_1~TPR_2~TPR_1~TPR_2~Peptidase_C14	B. japonicum;	Bll7042 protein. (Q89EN1);
TPR_1~TPR_2~TPR_1~TPR_2~TPR_4~TPR_2~Sulfotransfer_1	R. solanacearum;	PROBABLE TPR DOMAIN PROTEIN. (Q8XUM4);
TPR_2~BCSC_C	E. carotovora subsp. atroseptica SCRI1043; Pseudomonas fluorescens;	Cellulose synthase operon protein C precursor. (P58937*); Cellulose synthase protein C. (Q6CYY1);
TPR_2~STN~Secretin_N~Secretin~Cohesin	R. solanacearum;	PUTATIVE GSPD-RELATED PROTEIN. (Q8XTG8*);
TPR_2~TPR_1~Sulfotransfer_1	M . loti; X. axonopodis (pv. citri); uncultured bacterium 560;	TPR domain/sulfotransferase domain protein. (Q6SGF7); Mlr4028 protein. (Q98EY4); Hypothetical protein XAC3051. (Q8PI47);
TPR_2~TPR_1~TPR_2~TonB_dep_Rec	S. meliloti;	Hypothetical exported protein, TonB-dependent receptor protein. (Q92TP0*);
TPR_2~TPR_4~TPR_1~GerE	X. axonopodis (pv. citri);	Transcriptional regulator. (Q8PH39);
TPR_2~TPR_4~TPR_1~TPR_4	X. fastidiosa;	Hypothetical protein. (Q9PBH5);
TPR_2~TPR_4~TPR_2~TPR_4~TPR_2~BCSC_C	P. syringae (pv. tomato); X. axonopodis (pv. citri);	Cellulose synthase operon protein C precursor. (P58938*); Cellulose synthase operon protein C. (Q888J4);
TPR_2~TPR_4~TPR_2~TPR_4~TPR_2~Sulfotransfer_1	M . loti;	Mll2645 protein. (Q98HZ1);
TPR_3~Peptidase_M61~PDZ	X. axonopodis (pv. citri); X. campestris (pv. campestris);	Hypothetical protein XAC1262. (Q8PN12); Hypothetical protein XCC1163. (Q8PBF9);
TPR_4~TPR_2~SLT	B. japonicum;	Blr4770 protein. (Q89KX8);
TPR_4~TPR_2~TPR_1	B. japonicum;	Blr0663 protein. (Q89WL5);
TerD~Trypsin	P. syringae (pv. tomato);	Trypsin domain protein. (Q888T2);
Thioesterase~ACPS	R. solanacearum;	Hypothetical protein RSc1804. (Q8XYF4);

TniB	Alcaligenes eutrophus (Ralstonia eutropha); Comamonas acidovorans (Pseudomonas acidovorans); Enterobacter aerogenes (Aerobacter aerogenes); Escherichia coli; Klebsiella aerogenes; Klebsiella pneumoniae; Plasmid R100; Plasmid pSB102; P. aeruginosa; Pseudomonas sp. ED23-33; P. syringae (pv. tomato); M . loti; Ruegeria sp. PR1b; Shigella flexneri; Xanthomonas sp. W17;	TniB protein. (Q91UM6); Putative ATP-binding protein. (Q56452); TniB, putative NTP-binding protein. (Q9F3W0); TniB. (Q79BN4); TniB. (Q76M24); NTP-binding protein. (Q48383); Transposition helper protein. (Q88BF9); NTP-binding protein. (Q989U8); TniBdelta1. (Q8GEE8); NTP-binding protein. (Q7AKA2); TniBDelta1. (Q7BT56); TniBdelta1. (Q79CC9); Putative TniB-like transposition protein. (Q7WWT4); TniBdelta1. (Q57461); TniBdelta2. (O07818); TniB delta2 protein. (Q9R817); RC226. (Q8KVW4);
TraB_2	Agrobacterium rhizogenes; A. tumefaciens (strain 15955); A. tumefaciens ; A. tumefaciens; Oligotropha carboxidovorans (Pseudomonas carboxydovorans); Rhizobium etli; Rhizobium sp. (strain NGR234);	TraB. (Q44365); Conjugal transfer protein traB. (Q44351*); Tiorf111 protein. (Q9R6E8*); Probable conjugal transfer protein traB. (P55416*); Riorf114 protein. (Q9F5D8*); TraB protein. (Q93UY2*); Probable conjugal transfer protein TraB. (Q84HT5); Probable TraB. (Q6LB48); Hypothetical 16.6 kDa protein outside the virF region (ORF3). (P15596); TraB-like protein. (Q9F448);
TraC	Agrobacterium rhizogenes; A. tumefaciens ; A. tumefaciens; Brucella suis; Oligotropha carboxidovorans (Pseudomonas carboxydovorans); P. aeruginosa; Rhizobium etli; S. meliloti; Rhizobium sp. (strain NGR234);	Probable conjugal transfer protein traC. (P55419); Conjugal transfer protein traC. (Q44362); Conjugal transfer protein traC. (Q44348); Probable conjugal transfer protein TraC. (Q84HT8); Tiorf108 protein. (Q9R6F1); Riorf111 protein. (Q9F5E1); TraC protein. (Q93UY5); Probable TraC conjugal transfer protein. (Q92ZI1); Conjugal transfer protein. (Q8UKJ2); Conjugation transfer protein. (Q8KL70); AGR_pAT_162p. (Q7D3W3); Conjugal transfer protein TraC. (Q6LB51); TraC. (Q6X3G1); TraC protein. (Q8FWS4);
TraD	Agrobacterium rhizogenes; A. tumefaciens ; A. tumefaciens; Oligotropha carboxidovorans (Pseudomonas carboxydovorans); P. aeruginosa; Rhizobium etli; M . loti; S. meliloti; Rhizobium sp. (strain NGR234);	Probable conjugal transfer protein; TraD. (Q98LM5); Probable conjugal transfer protein traD. (P55420); Conjugal transfer protein traD. (Q44361); Conjugal transfer protein traD. (Q44347); Probable TraD. (Q6LB52); Probable conjugal transfer protein TraD. (Q84HT9); Tiorf107 protein. (Q9R6F2); Riorf110 protein. (Q9F5E2); TraD protein. (Q93UY6); TraD. (Q6X3G2); Conjugal transfer protein. (Q8UKJ3); Probable TraD conjugal transfer protein. (Q92ZI2); AGR_pAT_161p. (Q7D3W4); Conjugation transfer protein. (Q8KL69);
TraH_2	Agrobacterium rhizogenes; A. tumefaciens ; A. tumefaciens; Oligotropha carboxidovorans (Pseudomonas carboxydovorans); Rhizobium sp. (strain NGR234);	Hypothetical protein. (O68015); TraH. (Q44367); Conjugal transfer protein, Dtr system. (Q7CNW7); AGR_pTi_243p. (Q7D2G0); Tiorf112 protein. (Q9R6E7); TraH protein. (Q93UY1); Hypothetical protein riorf115. (Q9F5D7); Hypothetical 21.0 kDa protein y4dP. (P55415); TraH. (Q6LB47);
Trans_reg_C~HipA_N~TPR_2~TPR_4	M . loti;	Adenylate cyclase; Cya3. (Q98DL0);
Trans_reg_C~PD40	X. axonopodis (pv. citri); X. campestris (pv. campestris);	Hypothetical protein XCC3318. (Q8P5M0); Hypothetical protein XAC3446. (Q8PH14);
Trans_reg_C~TPR_1~TPR_2	B. japonicum;	Bll5196 protein. (Q89JS6); Bll1217 protein. (Q89V41); Blr7943 protein. (Q89C55);
Trans_reg_C~TPR_2~TPR_4~TPR_2~TPR_4	B. japonicum;	Transcriptional regulator. (Q89GS8);
Trans_reg_C~TPR_4	B. japonicum; M . loti; Ruegeria sp. PR1b;	Blr6255 protein. (Q89GU0); Putative transmembrane transcriptional regulator protein TtrR. (Q9JMX0); Transcriptional regulator. (Q89V61); Transcriptional regulator. (Q89XL4); RC156. (Q8KW34); Transcriptional regulator. (Q89XK9); Transcriptional regulator. (Q987X1); Blr4182 protein. (Q89ML1); Transcriptional regulator. (Q89K00); Transcriptional regulator. (Q89XL3);
Trans_reg_C~TPR_4~TPR_2	M . loti;	Transcriptional regulator. (Q988M2);
Transposase_8~Transposase_9~Transposase_20	B. japonicum;	Blr1657 protein. (Q89TW6);

TrbH	A. tumefaciens ; A. tumefaciens; Alcaligenes eutrophus (Ralstonia eutropha); Comamonas acidovorans (Pseudomonas acidovorans); Comamonas testosteroni (Pseudomonas testosteroni); Enterobacter aerogenes (Aerobacter aerogenes); Oligotropha carboxidovorans (Pseudomonas carboxydovorans); Plasmid RP4; Pseudomonas sp. (strain ADP); X. fastidiosa; uncultured bacterium;	TrbH. (Q76M34); Mating pair formation. (Q6UP57); TrbH protein. (Q7X3E6); TrbH. (P71183); TrbH protein. (Q79BQ1); Mating pair formation protein TrbH. (Q9AHH1); TrbH protein. (Q03542*); Conjugal transfer protein. (Q9PBT8*); Conjugal transfer protein. (Q9PHG6*); TrbH protein. (Q8RSJ0*); Tiorf8 protein. (Q9R6N9*); Conjugal transfer protein. (Q8U695); TrbH. (Q9R487); AGR_pTi_72p. (Q7D2Q0); Probable TrbH. (Q6LB41);
Trp_dioxygenase~Trp_dioxygenase	X. campestris (pv. campestris);	Tryptophan 2,3-dioxygenase. (Q8PBB3);
Trp_repressor~Sugar-bind	M . loti;	Transcriptional regulator. (Q98GB2);
UreE_N	Haloarcula marismortui (Halobacterium marismortui); P. syringae (pv. tomato);	Urease accessory protein UreE, putative. (Q883F1); Urease accessory protein UreE. (Q75ZQ1);
VWA~TPR_1	Alvinella pompejana epibiont 6C6; Desulfotalea psychrophila LSv54; X. campestris (pv. campestris);	Hypothetical membrane protein (BatB). (Q6AQK5); Hypothetical protein XCC3223. (Q8P5W2*); TPR domain protein. (Q6W3P8);
VirB7	Agrobacterium rhizogenes; A. tumefaciens ; A. tumefaciens, and A. tumefaciens (strain 15955); A. tumefaciens; M . loti;	Outer membrane lipoprotein virB7 precursor. (P09780); Outer membrane lipoprotein virB7 precursor. (P17797); PROBABLE VIRB7 TYPE IV SECRETION PROTEIN. (Q8KJL9*); VirB7. (O66287); Riorf159 protein. (Q9F596*); VirB7. (Q8VT96*); Tiorf147 protein. (Q7DKC7);
VirC1	Agrobacterium rhizogenes; A. tumefaciens ; A. tumefaciens; Rhizobium etli;	VirC1 protein. (P06665); VirC1 protein. (P07165); VirC1 protein. (P13459); Riorf166 protein. (Q9F589); VirC1. (Q8VT89); VirC1 protein. (Q9F451); VirC1. (Q9RP05); Tiorf154 protein. (Q7DKC2);
VirC2	Agrobacterium rhizogenes; A. tumefaciens ; A. tumefaciens; Rhizobium etli;	VirC2. (Q9RP06); VirC2 protein. (P06666); VirC2 protein. (P07166); VirC2 protein. (P13460); Tiorf153 protein. (Q7DKC3); Riorf165 protein. (Q9F590); VirC2. (Q8VT90);
VirD1	Agrobacterium rhizogenes; A. tumefaciens ; A. tumefaciens;	T-DNA border endonuclease virD1 (EC 3.1.-.-). (P06667); T-DNA border endonuclease virD1 (EC 3.1.-.-). (P18591); T-DNA border endonuclease virD1 (EC 3.1.-.-). (P13461); Tiorf155 protein. (Q7DKC1); Riorf167 protein. (Q9F588); VirD1. (Q8VT88);
VirE2	A. tumefaciens ; A. tumefaciens, and A. tumefaciens (strain 15955); A. tumefaciens; Rhizobium etli;	Single-strand DNA binding protein (63.5 kDa virulence protein). (P08062); Tiorf163 protein. (Q9R6B3); VirE2. (Q9RP03); Single-strand DNA binding protein. (P07544); VirE2. (Q8VT82);
VirE3	Agrobacterium rhizogenes; A. tumefaciens ; A. tumefaciens; Rhizobium etli;	Riorf173 protein. (Q9F582); VirE3. (Q8VT81); VirE3. (Q9RP02); VirA/G regulated protein. (Q8U5Y7); AGR_pTi_29p. (Q7D2S9); Tiorf164 protein. (Q9R6B2); VirE3. (Q44445); VirA/G regulated protein. (Q8U5Y8);
VirK	A. tumefaciens ; A. tumefaciens; B. japonicum; P. syringae (pv. tomato); R. solanacearum; Rhizobium sp. (strain NGR234); X. axonopodis (pv. citri); X. campestris (pv. campestris); X. fastidiosa (strain Temecula1 / ATCC 700964); X. fastidiosa;	VirK (Tiorf135 protein). (O50246*); VirA/G regulated gene. (Q7CNV8); Hypothetical 15.8 kDa protein in pinF2 3'region (ORF2). (Q44433*); Hypothetical 15.6 kDa protein y4WH. (P55686*); PUTATIVE SIGNAL PEPTIDE PROTEIN. (Q8XX33*); VirK protein. (Q8PDC2*); VirK protein. (Q8PQ93); ID299. (Q9ANE2*); Blr1847 protein. (Q79UP9); VirK protein. (Q87D31); VirK protein. (Q9PC40*); Hypothetical protein. (Q880Z8);
WD40~Cytochrom_C	B. japonicum;	Bll5485 protein. (Q89IZ9);
X_fast-SP_rel~HIM~HIM~X_fast-SP_rel~HIM~HIM~X_fast-SP_rel~HIM~HIM~X_fast-SP_rel~HIM~HIM~X_fast-SP_rel~HIM~HIM~X_fast-SP_rel~HIM~HIM~X_fast-SP_rel~HIM~HIM~HIM~Hep_Hag~HIM~Hep_Hag~HIM~YadA	X. fastidiosa (strain Temecula1 / ATCC 700964);	Surface protein. (Q87DE1);

XylR_N~V4R~Sigma54_activat~HTH_8	Acinetobacter calcoaceticus; Alcaligenes eutrophus (Ralstonia eutropha); Burkholderia kururiensis; Burkholderia pickettii (Pseudomonas pickettii); Burkholderia sp. RP007; Comamonas testosteroni (Pseudomonas testosteroni); Pseudomonas azelaica; Pseudomonas mendocina; Pseudomonas putida; Pseudomonas sp; Pseudomonas sp. KL28; Pseudomonas sp. OPS1; Pseudomonas sp. S-47; Pseudomonas stutzeri (Pseudomonas perfectomarina); P. syringae (pv. tomato); R. solanacearum; Ralstonia sp. E2; Ralstonia sp. JMP134; Sphingomonas aromaticivorans; Thauera aromatica;	Putative transcriptional activator HbpR. (O06645); Sigma-54 dependent transcriptional regulator. (Q881E3); PUTATIVE PHENOL-DEGRADATIVE GENE REGULATOR TRANSCRIPTION REGULATORPROTEIN. (Q8XT87); Transcriptional regulator. (Q8KP44); EugR. (Q8L387); Transcriptional activator TbuT. (P72311); MopR. (Q43965); LapR. (Q7WYF6); Putative transcriptional activator. (O69184); AphR protein. (Q9ZNN9); Regulator aromatic degradative pathways. (O85963); Positive regulator of phenol-degradative genes. (Q9S150); Positive phenol-degradative gene regulator. (O84957); Hypothetical protein. (Q9F2C0); Transcriptional regulator PhnR. (Q9ZHH9); Regulator protein PhkR. (Q8VUU3); DmpR protein. (Q06573); Activator. (Q52200); PhhR protein. (Q52177); TmbR. (Q51994); PheR protein. (Q52169); Positive regulator CapR. (Q7WSM9); Sigma 54-dependent transcriptional activator. (Q9R6Q4); Regulatory protein. (Q84I92); Transcriptional regulatory protein xylR (67 kDa protein). (P06519); Putative transcriptional activator. (Q84FP2);
YHS~YHS~E1-E2_ATPase~Hydrolase	Rhizobium leguminosarum (biovar viciae); M . loti;	Copper-transporting P-type ATPase (EC 3.6.3.4). (Q9X5V3); Cation transporting P-type ATPase. (Q98C24);
YceI~OmpA	M . loti;	Mll2374 protein. (Q98IJ5*);
adh_short~SEC-C	S. meliloti;	PUTATIVE TRANSMEMBRANE OXIDOREDUCTASE PROTEIN (EC 1.-.-.-). (Q92PY2);
cNMP_binding~CBS~DUF294	P. aeruginosa;	Hypothetical protein. (Q9HZ07);
cNMP_binding~Guanylate_cyc	B. japonicum;	Blr1670 protein. (Q89TV4);
ketoacyl-synt~Ketoacyl-synt_C~Acyl_transf_1~Thioesterase	M . loti;	Polyketide synthase. (Q982I5);
ketoacyl-synt~Ketoacyl-synt_C~PP-binding~PP-binding~Condensation	R. solanacearum;	PROBABLE POLYKETIDE SYNTHASE PROTEIN. (Q8XYE8);

OSJNBa0070D17.14 protein. (Q7XLT4); CAA303713.1 protein. (Q9ST84*); Putative peroxidase 40. (Q6ZHY7); Putative peroxidase. (Q6AVZ7); Putative peroxidase. (Q9FYP5); Putative peroxidase. (Q69QL4); Putative peroxidase. (Q9FYP0); Putative peroxidase. (Q6AVZ8); Putative peroxidase. (Q94H66*); Putative peroxidase. (Q8GVP1); Putative peroxidase. (Q6AUW9); Ascorbate peroxidase (EC 1.11.1.11). (O49822); Putative peroxidase. (Q8W2X2*); Putative Peroxidase 40. (Q6UU25); Peroxidase 20 precursor (EC 1.11.1.7) (Atperox P20) (ATP28a). (Q9SLH7*); Peroxidase. (Q75IS1); Putative peroxidase. (Q8GVN8); Putative peroxidase. (Q69XZ1); Peroxidase 11 precursor (EC 1.11.1.7) (Atperox P11) (ATP23a/ATP23b). (O96519*); Peroxidase. (Q9FEQ9); Putative peroxidase. (Q8GVG7); Putative peroxidase. (Q8LMR4); Peroxidase precursor (EC 1.11.1.7). (Q6PQF2); Peroxidase 58 precursor (EC 1.11.1.7) (Atperox P58) (ATP42). (P59120); Putative peroxidase. (Q8S5Y4*); Peroxidase 34 precursor (EC 1.11.1.7) (Atperox P34) (ATPCb). (Q9SMU8*); Peroxidase 19 precursor (EC 1.11.1.7) (Atperox P19) (ATP51). (O22959*); Peroxidase C1A precursor (EC 1.11.1.7). (P00433*); Peroxidase (EC 1.11.1.7). (Q43100*); Peroxidase 38 precursor (EC 1.11.1.7) (Atperox P38). (Q9LDA4*); Peroxidase 37 precursor (EC 1.11.1.7) (Atperox P37) (ATP38). (Q9LDN9*); Peroxidase (EC 1.11.1.7). (Q43101*); Peroxidase C1B precursor (EC 1.11.1.7). (P15232*); Peroxidase. (Q40950*); Peroxidase 32 precursor (EC 1.11.1.7) (Atperox P32) (PRXR3) (ATP16a). (Q9LHB9*); Peroxidase (EC 1.11.1.7). (Q43099*); Peroxidase 33 precursor (EC 1.11.1.7) (Atperox P33) (ATPCa) (Neutralperoxidase C) (PERC). (P24101*); Peroxidase (EC 1.11.1.7). (Q43050*); Peroxidase. (Q40949*); OSJNBa0035I04.3 protein (OSJNBb0088C09.13 protein). (Q7X766*); Peroxidase precursor (EC 1.11.1.7). (Q43158*); Putative thylakoid-bound ascorbate peroxidase. (Q6Z780); SPI2 protein (EC 1.11.1.7). (Q9SC55*); Peroxidase C2 precursor (EC 1.11.1.7). (P17179*); Peroxidase 24 precursor (EC 1.11.1.7) (Atperox P24) (ATP47). (Q9ZV04*); Peroxidase. (Q8S3U4*); Putative peroxidase. (Q948Z3*); Peroxidase 23 precursor (EC 1.11.1.7) (Atperox P23) (ATP34). (O80912*); Peroxidase 5. (Q9XFL6*); Putative peroxidase. (Q8S7Y4); Putative peroxidase. (Q8GVF7); Peroxidase precursor (EC 1.11.1.7). (Q6EVD0); Peroxidase C3 precursor (EC 1.11.1.7). (P17180*); Putative peroxidase. (Q6Z7S2); Putative peroxidase. (Q7XHB3); Peroxidase E5 (EC 1.11.1.7). (P59121); OSJNBa0060P14.16 protein (OSJNBb0048E02.7 protein). (Q7X8H7*); Peroxidase (EC 1.11.1.7). (Q43102*); Peroxidase precursor (EC 1.11.1.7). (Q07446*); Putative peroxidase. (Q6AVZ3); Peroxidase 35 precursor (EC 1.11.1.7) (Atperox P35) (ATP21a). (Q96510*); Peroxidase 10 precursor (EC 1.11.1.7) (Atperox P10) (ATP5a). (Q9FX85*); Putative peroxidase. (Q9FP11*); Similar to Arabidopsis thaliana peroxidase ATP19a (Putativeperoxidase). (Q9LGU0*); Putative peroxidase. (Q9FYP3); Peroxidase 1. (Q84UA9); Peroxidase precursor (EC 1.11.1.7). (Q9LEH3*); Peroxidase 50 precursor (EC 1.11.1.7) (Atperox P50) (PRXR2) (ATP9a). (Q43731*); Peroxidase 73 precursor (EC 1.11.1.7) (Atperox P73) (PRXR11) (ATP10a). (Q43873*); Peroxidase 22 precursor (EC 1.11.1.7) (Atperox P22) (ATPEa) (Basicperoxidase E). (P24102*); Peroxidase precursor (EC 1.11.1.7). (Q42905*); Peroxidase 51 precursor (EC 1.11.1.7) (Atperox P51) (ATP37). (Q9SZE7*); Peroidase (EC 1.11.1.7). (Q43049*); Peroxidase precursor (EC 1.11.1.7). (Q9ZP15*); Peroxidase1B precursor (EC 1.11.1.7). (Q43790*); Putative peroxidase. (Q9FYP1*); Extensin peroxidase. (Q8GZS1); Putative bacterial-induced peroxidase. (Q6EQJ7); Peroxidase 9 precursor (EC 1.11.1.7) (Atperox P9) (ATP18a). (Q96512*); Seed coat peroxidase precursor (EC 1.11.1.7). (O22443*); Peroxidase1C precursor (EC 1.11.1.7). (Q43791*); Putative peroxidase. (Q8RYP0*); ESTs AU081576 (R0541). (Q9LDY1*); Peroxidase precursor. (Q40366*); Peroxidase A2 (EC 1.11.1.7). (P80679); Peroxidase precursor (EC 1.11.1.7). (Q07445*); Peroxidase 29 precursor (EC 1.11.1.7) (Atperox P29) (ATP40). (Q9LSP0); Peroxidase 54 precursor (EC 1.11.1.7) (Atperox P54) (ATP29a). (Q9FG34*); Peroxidase (EC 1.11.1.7). (Q43774*); Putative peroxidase. (Q9FRD8*); Peroxidase 53 precursor (EC 1.11.1.7) (Atperox P53) (ATPA2). (Q42578); Apoplastic anionic gaiacol peroxidase (EC 1.11.1.7). (Q8RVP3*); Peroxidase. (Q94IQ0*); Peroxidase1A precursor (EC 1.11.1.7). (Q93XK6*); OSJNBa0071I13.13 protein. (Q7XPY0*); Peroxidase. (Q9LWA2*); Peroxidase1A precursor (EC 1.11.1.7). (O24081*); Peroxidase prx15 precursor (EC 1.11.1.7). (Q9M4Z2*); Peroxidase. (Q6AUX0); Peroxidase 72 precursor (EC 1.11.1.7) (Atperox P72) (PRXR8) (ATP6a). (Q9FJZ9*); Peroxidase 14 precursor (EC 1.11.1.7) (Atperox P14). (Q9SI17*); Peroxidase

rve~Transposase_11	P. syringae (pv. tomato);	ISPsy13, transposase OrfB. (Q87W63);
zf-C4_Topoisom~zf-C4_Topoisom~zf-C4_Topoisom~Sua5_yciO_yrdC	E. carotovora subsp. atroseptica SCRI1043;	Putative DNA topoisomerase. (Q6D005);

Table 5. 459 of the 3,774 distinct protein domain architectures found in R. solanacearum, P. aeruginosa, E. carotovora, P. syringae, B. japonicum, S. meliloti, M . loti, A. tumefaciens, X. fastidiosa, X. campestris, X. axonopodis that are absent from other fully-sequenced proteobacterial genomes.

Key:
2-Hacid_dh: D-isomer specific 2-hydroxyacid dehydrogenase, catalytic domain (PF00389); 3HCDH: 3-hydroxyacyl-CoA dehydrogenase, C-terminal domain (PF00725); 3HCDH_N: 3-hydroxyacyl-CoA dehydrogenase, NAD binding domain (PF02737); 4HBT: Thioesterase superfamily (PF03061); A2M_N: Alpha-2-macroglobulin family N-terminal region (PF01835); A2M_N_2: Alpha-2-macroglobulin family N-terminal region (PF07703); AA_kinase: Amino acid kinase family (PF00696); ABC2_membrane: ABC-2 type transporter (PF01061); ABC_tran: ABC transporter (PF00005); ABM: Antibiotic biosynthesis monooxygenase (PF03992); ACPS: 4'-phosphopantetheinyl transferase superfamily (PF01648); ACT: ACT domain (PF01842); ADH_zinc_N: Zinc-binding dehydrogenase (PF00107); AMP-binding: AMP-binding enzyme (PF00501); ANTAR: ANTAR domain (PF03861); AP_endonuc_2: Xylose isomerase-like TIM barrel (PF01261); ATP-grasp: ATP-grasp domain (PF02222); Abhydrolase_1: alpha/beta hydrolase fold (PF00561); Abhydrolase_2: Phospholipase/Carboxylesterase (PF02230); Acetyltransf_1: Acetyltransferase (GNAT) family (PF00583); Acyl-CoA_dh: Acyl-CoA dehydrogenase, C-terminal domain (PF00441); Acyl-CoA_dh_M: Acyl-CoA dehydrogenase, middle domain (PF02770); Acyl-CoA_dh_N: Acyl-CoA dehydrogenase, N-terminal domain (PF02771); Acyl_transf_1: Acyl transferase domain (PF00698); Acylphosphatase: Acylphosphatase (PF00708); Aegerolysin: Aegerolysin (PF06355); Agro_virD5: Agrobacterium VirD5 protein (PF04730); Ala_racemase_N: Alanine racemase, N-terminal domain (PF01168); Ald_Xan_dh_C: Aldehyde oxidase and xanthine dehydrogenase, a/b hammerhead domain (PF01315); Ald_Xan_dh_C2: Aldehyde oxidase and xanthine dehydrogenase, molybdopterin binding domain (PF02738); Aldedh: Aldehyde dehydrogenase family (PF00171); Aldo_ket_red: Aldo/keto reductase family (PF00248); Amino_oxidase: Flavin containing amine oxidoreductase (PF01593); Aminotran_3: Aminotransferase class-III (PF00202); Anthrax_toxA: Anthrax toxin LF subunit (PF03497); Antifreeze: Antifreeze-like domain (PF01354); Arg_tRNA_synt_N: Arginyl tRNA synthetase N terminal domain (PF03485); Arginase: Arginase family (PF00491); Asp_Arg_Hydrox: Aspartyl/Asparaginyl beta-hydroxylase (PF05118); Autoind_bind: Autoinducer binding domain (PF03472); Autotransporter: Autotransporter beta-domain (PF03797); Avidin: Avidin family (PF01382); Avirulence: Xanthomonas avirulence protein, Avr/PthA (PF03377); AvrB_AvrC: Avirulence protein (PF05394); BCSC_C: Cellulose synthase operon protein C C-terminus (BCSC_C) (PF05420); BNR: BNR/Asp-box repeat (PF02012); BPD_transp_2: Branched-chain amino acid transport system / permease component (PF02653); BTAD: Bacterial transcriptional activator domain (PF03704); B_lectin: D-mannose binding lectin (PF01453); Bac_luciferase: Luciferase-like monooxygenase (PF00296); Bcl-2: Apoptosis regulator proteins, Bcl-2 family (PF00452); Big_4: Bacterial Ig-like domain (group 4) (PF07532); Bro-N: BRO family, N-terminal domain (PF02498); CBM_14: Chitin binding Peritrophin-A domain (PF01607); CBM_2: Cellulose binding domain (PF00553); CBM_4_9: Carbohydrate binding domain (PF02018); CBM_5_12: Carbohydrate binding domain (PF02839); CBS: CBS domain (PF00571); CCG: Cysteine-rich domain (PF02754); CD225: Interferon-induced transmembrane protein (PF04505); CHASE: CHASE domain (PF03924); CHASE2: CHASE2 domain (PF05226); CHASE3: CHASE3 domain (PF05227); CHASE4: CHASE4 domain (PF05228); CMD: Carboxymuconolactone decarboxylase family (PF02627); COXG: Carbon monoxide dehydrogenase subunit G (CoxG) (PF06240); COesterase: Carboxylesterase (PF00135); Cache: Cache domain (PF02743); Cadherin: Cadherin domain (PF00028); Calx-beta: Calx-beta domain (PF03160); Cellulase: Cellulase (glycosyl hydrolase family 5) (PF00150); CheB_methylest: CheB methylesterase (PF01339); CheR: CheR methyltransferase, SAM binding domain (PF01739); CheR_N: CheR methyltransferase, all-alpha domain (PF03705); CheW: CheW-like domain (PF01584); Chitin_synth_1: Chitin synthase (PF01644); Chitin_synth_2: Chitin synthase (PF03142); Chorismate_bind: chorismate binding enzyme (PF00425); CoA_binding: CoA binding domain (PF02629); Cohesin: Cohesin domain (PF00963); Colicin: Colicin pore forming domain (PF01024); Collar: Phage Tail Collar Domain (PF07484); Condensation: Condensation domain (PF00668); Cu-oxidase: Multicopper oxidase (PF00394); Cu-oxidase_2: Multicopper oxidase (PF07731); Cu-oxidase_3: Multicopper oxidase (PF07732); Cupin_2: Cupin domain (PF07883); Cupin_3: Protein of unknown function (DUF861) (PF05899); Cys_Met_Meta_PP: Cys/Met metabolism PLP-dependent enzyme (PF01053); Cytochrom_C: Cytochrome c (PF00034); Cytochrom_D1: Cytochrome D1 heme domain (PF02239); DPBB_1: Rare lipoprotein A (RlpA)-like double-psi beta-barrel (PF03330); DUF1020: Protein of unknown function (DUF1020) (PF06255); DUF1036: Protein of unknown function (DUF1036) (PF06282); DUF1078: Domain of unknown function (DUF1078) (PF06429); DUF1205: Protein of unknown function (DUF1205) (PF06722); DUF1217: Protein of unknown function (DUF1217) (PF06748); DUF1236: Protein of unknown function (DUF1236) (PF06823); DUF1254: Protein of unknown function (DUF1254) (PF06863); DUF126: Protein of unknown function DUF126 (PF01989); DUF1289: Protein of unknown function (DUF1289) (PF06945); DUF1332: Protein of unknown function (DUF1332) (PF07049); DUF1403: Protein of unknown function (DUF1403) (PF07183); DUF1419: Protein of unknown function (DUF1419) (PF07215); DUF1427: Protein of unknown function (DUF1427) (PF07235); DUF1428: Protein of unknown function (DUF1428) (PF07237); DUF1458: Protein of unknown function (DUF1458) (PF07311); DUF1484: Protein of unknown function (DUF1484) (PF07363); DUF1486: Protein of unknown function (DUF1486) (PF07366); DUF1501: Protein of unknown function (DUF1501) (PF07394); DUF1515: Protein of unknown function (DUF1515) (PF07439); DUF1520: Domain of Unknown Function (DUF1520) (PF07480); DUF1521: Domain of Unknown Function (DUF1521) (PF07481); DUF1534: Protein of unknown function (DUF1534) (PF07551); DUF1629: Protein of unknown function (DUF1621) (PF07791); DUF239: Arabidopsis proteins of unknown function (PF03080); DUF294: Putative nucleotidyltransferase DUF294 (PF03445); DUF323: Domain of unknown function (DUF323) (PF03781); DUF336: Domain of unknown function (DUF336) (PF03928); DUF35: Domain of unknown function DUF35 (PF01796); DUF419: Protein of unknown function (DUF419) (PF04237); DUF427: Domain of unknown function (DUF427) (PF04248); DUF442: Protein of unknown function (DUF442) (PF04273); DUF521: Protein of unknown function (DUF521) (PF04412); DUF619: Protein of unknown function (DUF619) (PF04768); DUF637: Possible hemagglutinin (DUF637) (PF04830); DUF680: Protein of unknown function (DUF680) (PF05079); DUF726: Protein of unknown function (DUF726) (PF05277); DUF746: Domain of Unknown Function (DUF746) (PF05344); DUF748: Domain of Unknown Function (DUF748) (PF05359); DUF763: Protein of unknown function (DUF763) (PF05559); DUF768: Protein of unknown function (DUF768) (PF05589); DUF769: X. fastidiosa protein of unknown function (DUF769) (PF05590); DUF802: Domain of unknown function (DUF802) (PF05650); DUF811: Domain of unknown function (DUF811) (PF05665); DUF892: Protein of unknown function (DUF892) (PF05974); DUF982: Protein of unknown function (DUF982) (PF06169); DctM: TRAP C4-dicarboxylate transport (Dct) system permease DctM subunit (PF06808); DedA: DedA family (PF00597); DegT_DnrJ_EryC1: DegT/DnrJ/EryC1/StrS aminotransferase family (PF01041); Dehydratase_LU: Dehydratase large subunit (PF02286); Dehydratase_MU: Dehydratase medium subunit (PF02288); DnaJ: DnaJ domain (PF00226); DspF: DspF/AvrF protein (PF06704); E1-E2_ATPase: E1-E2 ATPase (PF00122); EAL: EAL domain (PF00563); Epimerase_2: UDP-N-acetylglucosamine 2-epimerase (PF02350); Erythro_esteras: Erythromycin esterase (PF05139); Exo_endo_phos: Endonuclease/Exonuclease/phosphatase family (PF03372); F-box: F-box domain (PF00646); F5_F8_type_C: F5/8 type C domain (PF00754); FAD-oxidase_C: FAD linked oxidases, C-terminal domain (PF02913); FAD_binding_4: FAD binding domain (PF01565); FAD_binding_6: Oxidoreductase FAD-binding domain (PF00970); FA_desaturase: Fatty acid desaturase (PF00487); FCD: FCD domain (PF07729); FdtA: WxcM-like, C-terminal (PF05523); Fer2: 2Fe-2S iron-sulfur cluster binding domain (PF00111); Fer2_2: [2Fe-2S] binding domain (PF01799); Fer2_BFD: BFD-like [2Fe-2S] binding domain (PF04324); Fer4: 4Fe-4S binding domain (PF00037); Fic: Fic protein family (PF02661); Fil_haemagg: Haemagglutinin repeat (PF05594); FlaE: Flagellar basal body protein FlaE (PF07559); Flagellin_IN: Flagellin hook IN motif (PF07196); Flavodoxin_1: Flavodoxin (PF00258); Flg_bb_rod: Flagella basal body rod protein (PF00460); FliD_C: Flagellar hook-associated protein 2 C-terminus (PF07195); FliD_N: Flagellar hook-associated protein 2 C-terminus (PF02465); FmdA_AmdA: Acetamidase/Formamidase family (PF03069); GAF: GAF domain (PF01590); GATase: Glutamine amidotransferase class-I (PF00117); GDA1_CD39: GDA1/CD39 (nucleoside phosphatase) family (PF01150); GFO_IDH_MocA: Oxidoreductase family, NAD-binding Rossmann fold (PF01408); GFO_IDH_MocA_C: Oxidoreductase family, C-terminal alpha/beta domain (PF02894); GGDEF: GGDEF domain (PF00990); GHMP_kinases: GHMP kinases putative ATP-binding protein (PF00288); GLF: UDP-galactopyranose mutase (PF03275); GMC_oxred_N: GMC oxidoreductase (PF00732); GXGXG: GXGXG motif (PF01493); Gamma-BBH: Gamma-butyrobetaine hydroxylase (PF03322); GerE: Bacterial regulatory proteins, luxR family (PF00196); GlcNAc_2-epim: N-acylglucosamine 2-epimerase (GlcNAc 2-epimerase) (PF07221); Gln-synt_N: Glutamine synthetase, beta-Grasp domain (PF03951); Glug: The GLUG motif (PF07581); Glutaminase: Glutaminase (PF04960); Glutaredoxin: Glutaredoxin (PF00462); Glyco_hydro_16: Glycosyl hydrolases family 16 (PF00722); Glyco_hydro_18: Glycosyl hydrolases family 18 (PF00704); Glyco_hydro_53: Glycosyl hydrolase family 53 (PF07745); Glyco_hydro_6: Glycosyl hydrolases family 6 (PF01341); Glyco_transf_25: Glycosyltransferase family 25 (LPS biosynthesis protein) (PF01755); Glyco_transf_28: Glycosyltransferase family 28 N-terminal domain (PF03033); Glyco_transf_36: Glycosyltransferase family 36 (PF06165); Glycos_trans_3N: Glycosyl transferase family, helical bundle domain (PF02885); Glycos_transf_1: Glycosyl transferases group 1 (PF00534); Glycos_transf_2: Glycosyl transferase (PF00535); Glycos_transf_3: Glycosyl transferase family, a/b domain (PF00591); Glyoxalase: Glyoxalase/Bleomycin resistance protein/Dioxygenase superfamily (PF00903); Glyphos_transf: CDP-Glycerol:Poly(glycerophosphate) glycerophosphotransferase (PF04464); GntR: Bacterial regulatory proteins, gntR family (PF00392); Guanylate_cyc: Adenylate and Guanylate cyclase catalytic domain (PF00211); H-kinase_dim: Signal transducing histidine kinase, homodimeric domain (PF02895); HA2: Helicase associated domain (HA2) (PF04408); HAMP: HAMP domain (PF00672); HATPase_c: Histidine kinase-, DNA gyrase B-, and HSP90-like ATPase (PF02518); HCBP_related: Haemolysin-type calcium binding protein related domain (PF06594); HD: HD domain (PF01966); HEAT: HEAT repeat (PF02985); HEM4: Uroporphyrinogen-III synthase HemD (PF02602); HIM: Haemagglutinin (PF05662); HSDR_N: Type I restriction enzyme R protein N terminus (HSDR_N) (PF04313); HTH_1: Bacterial regulatory helix-turn-helix protein, lysR family (PF00126); HTH_3: Helix-turn-helix (PF01381); HTH_8: Bacterial regulatory protein, Fis family (PF02954); HTH_AraC: Bacterial regulatory helix-turn-helix proteins, AraC family (PF00165); HWE_HK: HWE histidine kinase (PF07536); Haemagg_act: haemagglutination activity domain (PF05860); Harpin: Harpin protein (HrpN) (PF07132); He_PIG: Putative Ig domain (PF05345); Helicase_C: Helicase conserved C-terminal domain (PF00271); HemX: HemX (PF04375); HemY_N: HemY protein N-terminus (PF07219); HemolysinCabind: Hemolysin-type calcium-binding repeat (2 copies) (PF00353); Hep_Hag: Hep_Hag (PF05658); Het-C: Heterokaryon incompatibility protein Het-C (PF07217); Hexapep: Bacterial transferase hexapeptide (three repeats) (PF00132); HipA_N: HipA-like N-terminal domain (PF07805); HisKA: His Kinase A (phosphoacceptor) domain (PF00512); HisKA_2: Histidine kinase (PF07568); HisKA_3: Histidine kinase (PF07730); Hpt: Hpt domain (PF01627); HrpE: HrpE protein (PF06188); HrpF: HrpF protein (PF06266); HrpZ: HrpZ (PF04877); HupF_HypC: HupF/HypC family (PF01455); Hydrolase: haloacid dehalogenase-like hydrolase (PF00702); Ice_nucleation: Ice nucleation protein repeat (PF00818); IclR: Bacterial transcriptional regulator (PF01614); Ion_trans_2: Ion channel (PF07885); Isochorismatase: Isochorismatase family (PF00857); KAP_NTPase: KAP family P-loop domain (PF07693); Kazal_2: Kazal-type serine protease inhibitor domain (PF07648); Kelch_1: Kelch motif (PF01344); Kelch_2: Kelch motif (PF07646); Ketoacyl-synt_C: Beta-ketoacyl synthase, C-terminal domain (PF02801); LRR_1: Leucine Rich Repeat (PF00560); LacI: Bacterial regulatory proteins, lacI family (PF00356); Lactamase_B: Metallo-beta-lactamase superfamily (PF00753); Lipase_GDSL: GDSL-like Lipase/Acylhydrolase (PF00657); Lipoprotein_5: Transferrin binding protein-like solute binding protein (PF01298); Lipoprotein_9: NLPA lipoprotein (PF03180); MAAL_N: Methylaspartate ammonia-lyase N-terminus (PF05034); MASE1: MASE1 (PF05231); MASE2: MASE2 domain (PF05230); MCD:

Malonyl-CoA decarboxylase (MCD) (PF05292); MCE: mce related protein (PF02470); MCPsignal: Methyl-accepting chemotaxis protein (MCP) signaling domain (PF00015); MHYT: Bacterial signalling protein N terminal repeat (PF03707); MLTD_N: MLTD_N (PF06474); MOSC: MOSC domain (PF03473); MR_MLE: Mandelate racemase / muconate lactonizing enzyme, C-terminal domain (PF01188); MR_MLE_N: Mandelate racemase / muconate lactonizing enzyme, N-terminal domain (PF02746); MTTB: Trimethylamine methyltransferase (MTTB) (PF06253); MannoseP_isomer: Mannose-6-phosphate isomerase (PF01050); Met_synt_B12: Vitamin B12 dependent methionine synthase, activation domain (PF02965); Metallophos: Calcineurin-like phosphoesterase (PF00149); Microcin: Colicin E1 (microcin) immunity protein (PF03526); MmoB_DmpM: MmoB/DmpM family (PF02406); Mn_catalase: Manganese containing catalase (PF05067); MobA_MobL: MobA/MobL family (PF03389); Molybdop_Fe4S4: Molybdopterin oxidoreductase Fe4S4 domain (PF04879); Molybdopterin: Molybdopterin oxidoreductase (PF00384); Molydop_binding: Molydopterin dinucleotide binding domain (PF01568); NAD_binding_1: Oxidoreductase NAD-binding domain (PF00175); NHL: NHL repeat (PF01436); NIR_SIR: Nitrite and sulphite reductase 4Fe-4S domain (PF01077); NIR_SIR_ferr: Nitrite/Sulfite reductase ferredoxin-like half domain (PF03460); NTP_transf_2: Nucleotidyltransferase domain (PF01909); NTP_transferase: Nucleotidyl transferase (PF00483); NUDIX: NUDIX domain (PF00293); NeuB: NeuB family (PF03102); Ni_hydr_CYTB: Cytochrome b561 family (PF01292); NodA: Nodulation protein A (NodA) (PF02474); NodS: Nodulation protein S (NodS) (PF05401); NodZ: Nodulation protein Z (NodZ) (PF05830); NolV: Nodulation protein NolV (PF06635); NolX: NolX protein (PF05819); OCD_Mu_crystall: Ornithine cyclodeaminase/mu-crystallin family (PF02423); OEP: Outer membrane efflux protein (PF02321); OmpA: OmpA family (PF00691); Orn_Arg_deC_N: Pyridoxal-dependent decarboxylase, pyridoxal binding domain (PF02784); Orn_DAP_Arg_deC: Pyridoxal-dependent decarboxylase, C-terminal sheet domain (PF00278); Oxidored_nitro: Nitrogenase component 1 type Oxidoreductase (PF00148); PAC: PAC motif (PF00785); PALP: Pyridoxal-phosphate dependent enzyme (PF00291); PAN: PAN domain (PF00024); PAP2: PAP2 superfamily (PF01569); PAPS_reduct: Phosphoadenosine phosphosulfate reductase family (PF01507); PAS: PAS domain (PF00989); PAX: 'Paired box' domain (PF00292); PCMT: Protein-L-isoaspartate(D-aspartate) O-methyltransferase (PCMT) (PF01135); PD40: WD40-like Beta Propeller Repeat (PF07676); PDZ: PDZ domain (Also known as DHR or GLGF) (PF00595); PEP-utilisers_N: PEP-utilising enzyme, N-terminal (PF05524); PEP-utilizers: PEP-utilising enzyme, mobile domain (PF00391); PEP-utilizers_C: PEP-utilising enzyme, TIM barrel domain (PF02896); PGI: Phosphoglucose isomerase (PF00342); PG_binding_1: Putative peptidoglycan binding domain (PF01471); PHZA_PHZB: Phenazine biosynthesis protein A/B (PF03284); PP-binding: Phosphopantetheine attachment site (PF00550); PPC: Bacterial pre-peptidase C-terminal domain (PF04151); PPR: PPR repeat (PF01535); PRC: PRC-barrel domain (PF05239); PS_Dcarbxylase: Phosphatidylserine decarboxylase (PF02666); PTS-HPr: PTS HPr component phosphorylation site (PF00381); PTS_EIIA_2: Phosphoenolpyruvate-dependent sugar phosphotransferase system, EIIA 2 (PF00359); PepSY_TM: PepSY-associated TM helix (PF03929); Peptidase_C14: Caspase domain (PF00656); Peptidase_C39: Peptidase C39 family (PF03412); Peptidase_C48: Ulp1 protease family, C-terminal catalytic domain (PF02902); Peptidase_M10: Matrixin (PF00413); Peptidase_M13: Peptidase family M13 (PF01431); Peptidase_M22: Glycoprotease family (PF00814); Peptidase_M23: Peptidase family M23 (PF01551); Peptidase_M28: Peptidase family M28 (PF04389); Peptidase_M61: M61 glycyl aminopeptidase (PF05299); Peptidase_S8: Subtilase family (PF00082); Peripla_BP_1: Periplasmic binding proteins and sugar binding domain of the LacI family (PF00532); PhaC_N: Poly-beta-hydroxybutyrate polymerase (PhaC) N-terminus (PF07167); Phage_Coat_B: Phage Coat protein B (PF05356); Phage_DNA_bind: Helix-destabilising protein (PF02303); Phage_T7_tail: Phage T7 tail fibre protein (PF03906); Phage_holin_2: Phage holin family 2 (PF04550); Phage_integr_N: Phage integrase, N-terminal SAM-like domain (PF02899); Phage_integrase: Phage integrase family (PF00589); Phenol_Hydrox: Methane/Phenol/Toluene Hydroxylase (PF02332); Phytochrome: Phytochrome region (PF00360); Pilin: Pilin (bacterial filament) (PF00114); Pkinase: Protein kinase domain (PF00069); Pkinase_Tyr: Protein tyrosine kinase (PF07714); PolyA_pol: Poly A polymerase family (PF01743); Poly_export: Polysaccharide biosynthesis/export protein (PF02563); Polysacc_deac_1: Polysaccharide deacetylase (PF01522); Pro_3_hydrox_C: L-proline 3-hydroxylase, C-terminal (PF05373); Pyr_redox: Pyridine nucleotide-disulphide oxidoreductase (PF00070); RHS_repeat: RHS Repeat (PF05593); RNA_pol_Rpb6: RNA polymerase Rpb6 (PF01192); RVP: Retroviral aspartyl protease (PF00077); Radical_SAM: Radical SAM superfamily (PF04055); Reg_prop: Two component regulator propeller (PF07494); RepA_C: Plasmid encoded RepA protein (PF04796); RepB: RepB plasmid partitioning protein (PF07506); Response_reg: Response regulator receiver domain (PF00072); RgpF: Rhamnan synthesis protein F (PF05045); Rhamnogal_lyase: Rhamnogalacturonate lyase family (PF06045); Rhodanese: Rhodanese-like domain (PF00581); Ribosomal_60s: 60s Acidic ribosomal protein (PF00428); Ribosomal_L12: Ribosomal protein L7/L12 C-terminal domain (PF00542); Ricin_B_lectin: QXW lectin repeat (PF00652); Rieske: Rieske [2Fe-2S] domain (PF00355); RolB_RolC: RolB/RolC glucosidase family (PF02027); SAM_1: SAM domain (Sterile alpha motif) (PF00536); SBP56: 56kDa selenium binding protein (SBP56) (PF05694); SBP_bac_3: Bacterial extracellular solute-binding proteins, family 3 (PF00497); SBP_bac_7: Bacterial extracellular solute-binding protein, family 7 (PF03480); SEC-C: SEC-C motif (PF02810); SLT: Transglycosylase SLT domain (PF01464); SMP-30: Senescence marker protein-30 (SMP-30) (PF03758); ST7: ST7 protein (PF04184); START: START domain (PF01852); STAS: STAS domain (PF01740); STN: Secretin and TonB N terminus short domain (PF07660); SdiA-regulated: SdiA-regulated (PF06977); SecIII_HopPtoS: Type III effector HopPtoS (PF07518); Secretin: Bacterial type II and III secretion system protein (PF00263); Secretin_N: Bacterial type II/III secretion system short domain (PF03958); Sigma54_DBD: Sigma-54, DNA binding domain (PF04552); Sigma54_activat: Sigma-54 interaction domain (PF00158); Sigma70_r1_1: Sigma-70 factor, region 1.1 (PF03979); SpoIIE: Stage II sporulation protein E (SpoIIE) (PF07228); Str_synth: Strictosidine synthase (PF03088); Sua5_yciO_yrdC: yrdC domain (PF01300); Succ_DH_flav_C: Fumarate reductase/succinate dehydrogenase flavoprotein C-terminal domain (PF02910); Sugar-bind: Putative sugar-binding domain (PF04198); Sulfate_transp: Sulfate transporter family (PF00916); Sulfotransfer_1: Sulfotransferase domain (PF00685); TFR_dimer: Transferrin receptor-like dimerisation domain (PF04253); TIG: IPT/TIG domain (PF01833); TMP-TENI: Thiamine monophosphate synthase/TENI (PF02581); TM_helix: Conserved TM helix (PF05552); TPR_1: Tetratricopeptide repeat (PF00515); TPR_2: Tetratricopeptide repeat (PF07719); TPR_3: Tetratricopeptide repeat (PF07720); TPR_4: Tetratricopeptide repeat (PF07721); TP_methylase: Tetrapyrrole (Corrin/Porphyrin) Methylases (PF00590); TerD: Bacterial stress protein (PF02342); Thioesterase: Thioesterase domain (PF00975); TniB: Bacterial TniB protein (PF05621); TonB_dep_Rec: TonB dependent receptor (PF00593); TraB_2: TraB protein (PF07186); TraC: TraC-like protein (PF07820); TraD: Conjugal transfer protein TraD (PF06412); TraH_2: TraH_2 (PF06871); Trans_reg_C: Transcriptional regulatory protein, C terminal (PF00486); Transposase_11: Transposase DDE domain (PF01609); Transposase_20: Transposase IS116/IS110/IS902 family (PF02371); Transposase_8: Transposase (PF01527); Transposase_9: Transposase (PF01548); TrbH: Conjugal transfer protein TrbH (PF07283); Trp_dioxygenase: Tryptophan 2,3-dioxygenase (PF03301); Trp_repressor: Trp repressor protein (PF01371); Trypsin: Trypsin (PF00089); UDPG_MGDP_dh: UDP-glucose/GDP-mannose dehydrogenase family, central domain (PF00984); UDPG_MGDP_dh_C: UDP-glucose/GDP-mannose dehydrogenase family, UDP binding domain (PF03720); UDPG_MGDP_dh_N: UDP-glucose/GDP-mannose dehydrogenase family, NAD binding domain (PF03721); UPF0261: Uncharacterised protein family (UPF0261) (PF06792); UreE_N: UreE urease accessory protein, N-terminal domain (PF02814); V4R: V4R domain (PF02830); VWA: von Willebrand factor type A domain (PF00092); VirB7: VirB7 protein (PF07269); VirC1: VirC1 protein (PF07015); VirC2: VirC2 protein (PF07181); VirD1: T-DNA border endonuclease VirD1 (PF07328); VirE2: VirE2 (PF07229); VirE3: VirE3 (PF06661); VirK: VirK protein (PF06903); Viral_helicase1: Viral (Superfamily 1) RNA helicase (PF01443); WD40: WD domain, G-beta repeat (PF00400); W_rich_C: Tryptophan-rich Synechocystis species C-terminal domain (PF07483); X_fast-SP_rel: X. fastidiosa surface protein related (PF06669); XylR_N: Activator of aromatic catabolism (PF06505); YHS: YHS domain (PF04945); Y_Y_Y: Two component regulator three Y motif (PF07495); YadA: YadA-like C-terminal region (PF03895); YceI: YceI like family (PF04264); adh_short: short chain dehydrogenase (PF00106); cNMP_binding: Cyclic nucleotide-binding domain (PF00027); fn3: Fibronectin type III domain (PF00041); ketoacyl-synt: Beta-ketoacyl synthase, N-terminal domain (PF00109); peroxidase: Peroxidase (PF00141); rve: Integrase core domain (PF00665); tRNA-synt_1d: tRNA synthetases class I (R) (PF00750); zf-C4_Topoisom: Topoisomerase DNA binding C4 zinc finger (PF01396);
