# Supplementary material for: Protein domains and architectural innovation in plant-associated Proteobacteria
Source: BMC Genomics. 2005 Feb 16;6:17. doi: 10.1186/1471-2164-6-17 (PMC554113; doi:10.1186/1471-2164-6-17)
Supplement: Additional File 2 — Prokaryotic genomes included in Pfam16.0 (and hence in this study). [file 1471-2164-6-17-S2.pdf]

Species	Number of proteins
Streptomyces coelicolor	5355
Acinetobacter sp. (strain ADP1)	2388
Aeropyrum pernix	1070
Agrobacterium tumefaciens (strain C58 / ATCC 33970 (Washington University))	4077
Agrobacterium tumefaciens (strain C58 / ATCC 33970)	4073
Anabaena sp. (strain PCC 7120)	3612
Aquifex aeolicus	1235
Archaeoglobus fulgidus	1723
Bacillus anthracis (strain Ames)	3344
Bacillus anthracis str. Ames 0581	3401
Bacillus anthracis str. Sterne	3544
Bacillus cereus (strain ATCC 10987)	3603
Bacillus cereus (strain ATCC 14579 / DSM 31)	3434
Bacillus halodurans	2927
Bacillus subtilis	2992
Bacillus thuringiensis (subsp. konkukian)	3511
Bacteroides thetaiotaomicron	2828
Bartonella henselae (Rochalimaea henselae)	1064
Bartonella quintana (Rochalimaea quintana) (Rochalimaea quintana)	931
Bdellovibrio bacteriovorus	1947
Bifidobacterium longum	1293
Bordetella bronchiseptica (Alcaligenes bronchisepticus)	3988
Bordetella parapertussis	3455
Bordetella pertussis	2711
Borrelia burgdorferi (Lyme disease spirochete) (Lyme disease spirochete)	985
Bradyrhizobium japonicum	5652
Brucella melitensis	2331
Buchnera aphidicola (subsp. Acyrthosiphon pisum) (Acyrthosiphon pisum symbiotic bacterium)	547
Buchnera aphidicola (subsp. Baizongia pistaciae)	483
Buchnera aphidicola (subsp. Schizaphis graminum)	541
Campylobacter jejuni	1208

Species	Number of proteins
Candidatus Blochmannia floridanus	546
Caulobacter crescentus	2693
Chlamydia muridarum	648
Chlamydia pneumoniae strain AR39	712
Chlamydia pneumoniae strain CWL029	715
Chlamydia pneumoniae strain J138	724
Chlamydia trachomatis	641
Chlamydophila caviae	687
Chlamydophila pneumoniae TW-183	719
Chlorobium tepidum	1438
Chromobacterium violaceum	3105
Clostridium acetobutylicum	2652
Clostridium perfringens	2001
Clostridium tetani	1796
Corynebacterium diphtheriae	1506
Corynebacterium efficiens	1967
Corynebacterium glutamicum (Brevibacterium flavum)	1992
Corynebacterium glutamicum (Brevibacterium flavum) IS fingerprint type 4-5	1962
Coxiella burnetii	1171
Deinococcus radiodurans	2057
Desulfovibrio vulgaris (strain Hildenborough / ATCC 29579 / NCIMB 8303)	2186
Enterococcus faecalis (Streptococcus faecalis) (Streptococcus faecalis)	2117
Erwinia carotovora (subsp. atroseptica) (Pectobacterium atrosepticum) (Pectobacterium atrosepticum)	3482
Escherichia coli	3513
Escherichia coli O157:H7	4016
Escherichia coli O157:H7 strain EDL933	4011
Escherichia coli O6	3766
Fusobacterium nucleatum (subsp. nucleatum)	1437
Geobacter sulfurreducens	2375
Gloeobacter violaceus	2809
Haemophilus ducreyi	1176

**Species**	**Number of proteins**
Haemophilus influenzae	1447
Halobacterium sp. (strain NRC-1 / ATCC 700922 / JCM 11081)	1472
Helicobacter hepaticus	1173
Helicobacter pylori (Campylobacter pylori) (Campylobacter pylori)	1058
Helicobacter pylori J99 (Campylobacter pylori J99)	1063
Lactobacillus johnsonii	1329
Lactobacillus plantarum	2208
Lactococcus lactis (subsp. lactis) (Streptococcus lactis) (Streptococcus lactis)	1694
Leifsonia xyli (subsp. xyli)	1379
Leptospira interrogans	2261
Leptospira interrogans (serogroup Icterohaemorrhagiae / serovar Copenhageni)	2076
Listeria innocua	2253
Listeria monocytogenes	2215
Listeria monocytogenes (serotype 4b / strain F2365)	2173
Mesoplasma florum (Acholeplasma florum) (Acholeplasma florum)	494
Methanobacterium thermoautotrophicum	1330
Methanococcus jannaschii	1267
Methanococcus maripaludis	1267
Methanopyrus kandleri	1071
Methanosarcina acetivorans	2682
Methanosarcina mazei (Methanosarcina frisia) (Methanosarcina frisia)	2144
Mycobacterium bovis	2199
Mycobacterium leprae	1144
Mycobacterium paratuberculosis	3070
Mycobacterium tuberculosis	2230
Mycobacterium tuberculosis CDC1551	2150
Mycoplasma gallisepticum	479
Mycoplasma genitalium	376
Mycoplasma mobile	430
Mycoplasma mycoides (subsp. mycoides SC)	590
Mycoplasma penetrans	666
Mycoplasma pneumoniae	510

Species	Number of proteins
Mycoplasma pulmonis	501
Nanoarchaeum equitans	318
Neisseria meningitidis (serogroup A)	1371
Neisseria meningitidis (serogroup B)	1332
Nitrosomonas europaea	1761
Oceanobacillus iheyensis	2592
Onion yellows phytoplasma	430
Parachlamydia sp. (strain UWE25) (subsp. Acanthamoeba sp.)	1143
Pasteurella multocida	1712
Photobacterium profundum (Photobacterium sp. (strain SS9))	3744
Photorhabdus luminescens (subsp. laumondii)	3077
Picrophilus torridus	1094
Porphyromonas gingivalis (Bacteroides gingivalis) (Bacteroides gingivalis)	1160
Prochlorococcus marinus	1154
Prochlorococcus marinus (strain MIT 9313)	1425
Prochlorococcus marinus subsp. pastoris (strain CCMP 1378 / MED4)	1129
Propionibacterium acnes	1627
Pseudomonas aeruginosa	4324
Pseudomonas putida (strain KT2440)	3982
Pseudomonas syringae (pv. tomato)	3863
Pyrobaculum aerophilum	1416
Pyrococcus abyssi	1409
Pyrococcus furiosus	1480
Pyrococcus horikoshii	1315
Ralstonia solanacearum (Pseudomonas solanacearum) (Pseudomonas solanacearum)	3534
Rhizobium loti (Mesorhizobium loti)	5126
Rhizobium meliloti (Sinorhizobium meliloti) (Sinorhizobium meliloti)	4694
Rhodopirellula baltica	3174
Rhodopseudomonas palustris	3502
Rickettsia conorii	774
Rickettsia prowazekii	677
Salmonella enterica subsp. enterica serovar Typhi Ty2	3235

Species	Number of proteins
Salmonella typhi	3381
Salmonella typhimurium	3566
Shewanella oneidensis	2925
Shigella flexneri	3308
Shigella flexneri 2a str. 2457T	3122
Staphylococcus aureus (strain MRSA252)	1969
Staphylococcus aureus (strain MSSA476)	1966
Staphylococcus aureus (strain Mu50 / ATCC 700699)	1680
Staphylococcus aureus (strain N315)	1619
Staphylococcus epidermidis	1784
Streptococcus agalactiae (serotype III)	1468
Streptococcus agalactiae (serotype V)	1489
Streptococcus mutans	1452
Streptococcus pneumoniae	1415
Streptococcus pneumoniae (strain ATCC BAA-255 / R6)	1451
Streptococcus pyogenes	1186
Streptococcus pyogenes (serotype M18)	1250
Streptococcus pyogenes (serotype M3)	1213
Streptococcus pyogenes SSI-1	1228
Streptomyces avermitilis	5041
Sulfolobus solfataricus	1929
Sulfolobus tokodaii	1660
Synechococcus elongatus (Thermosynechococcus elongatus)	1766
Synechococcus sp. (strain WH8102)	1522
Synechocystis sp. (strain PCC 6803)	2355
Thermoanaerobacter tengcongensis	1907
Thermoplasma acidophilum	1086
Thermoplasma volcanium	1080
Thermotoga maritima	1460
Thermus thermophilus (strain HB27 / ATCC BAA-163 / DSM 7039)	1649
Treponema denticola	1532
Treponema pallidum	696
Tropheryma whipplei (strain TW08/27) (Whipple's bacillus)	590

**Species**	**Number of proteins**
Tropheryma whipplei (strain Twist) (Whipple's bacillus)	590
Ureaplasma parvum (Ureaplasma urealyticum biotype 1) (Ureaplasma urealyticum biotype 1)	398
Vibrio cholerae	2737
Vibrio parahaemolyticus	3373
Vibrio vulnificus	3299
Vibrio vulnificus (strain YJ016)	3318
Wigglesworthia glossinidia brevipalpis	575
Wolbachia pipientis wMel	705
Wolinella succinogenes	1523
Xanthomonas axonopodis (pv. citri)	3074
Xanthomonas campestris (pv. campestris)	2990
Xylella fastidiosa	1553
Xylella fastidiosa (strain Temecula1 / ATCC 700964)	1442
Yersinia pestis	3031
Yersinia pestis biovar Mediaevails	3004
Yersinia pestis KIM	2978

Table 6. Completely sequenced prokaryotic genomes available in Pfam at the time of this study.
